# Enrichment of mutant calmodulin protein in a murine model of a human calmodulinopathy

**DOI:** 10.1172/jci.insight.185524

**Published:** 2025-07-24

**Authors:** Wen-Chin Tsai, Chiu-Fen Yang, Shu-Yu Lin, Suh-Yuen Liang, Wei-Chung Tsai, Shuai Guo, Xiaochun Li, Susan Ofner, Kai-Chien Yang, Tzu-Ching Meng, Peng-Sheng Chen, Michael Rubart

**Affiliations:** 1 Department of Cardiology, Cardiovascular Research Center, Hualien Tzu Chi Hospital, Buddhist Tzu Chi Medical Foundation, Hualien, Taiwan.; 2 Department of Pediatrics, Riley Heart Research Center, Indiana University School of Medicine, Indianapolis, Indiana, USA.; 3 Institute of Biological Chemistry, Academia Sinica, Taipei, Taiwan.; 4 Academia Sinica Common Mass Spectrometry Facilities for Proteomics and Protein Modification Analysis, Taipei, Taiwan.; 5 Bioinformatics Core Institute of Biological Chemistry, Academia Sinica, Taipei, Taiwan.; 6 Division of Cardiology, Department of Medicine, Kaohsiung Medical University Hospital, Kaohsiung Medical University, Kaohsiung, Taiwan.; 7 Department of Biostatistics and Health Data Science, Indiana University School of Medicine, Indianapolis, Indiana, USA.; 8 Graduate Institute and Department of Pharmacology, National Taiwan University College of Medicine, Taipei, Taiwan.; 9 Institute of Biomedical Sciences, Academia Sinica, Taipei, Taiwan.; 10 Department of Cardiology, Smidt Heart Institute, Cedars-Sinai Medical Center, Los Angeles, California, USA.

**Keywords:** Cardiology, Cell biology, Calmodulin

## Abstract

Heterozygosity for missense mutations in 1 of 3 seemingly redundant calmodulin-encoding (CALM-encoding) genes can cause life-threatening arrhythmias, suggesting that small fractions of mutant CALM protein suffice to cause a severe phenotype. However, the exact molar ratios of wild-type to mutant CALM protein in calmodulinopathy hearts remain unknown. The aim of the present study was to quantitate mutant versus wild-type CALM transcript and protein levels in hearts of knockin mice harboring the p.N98S mutation in the *Calm1* gene. We found that the transcripts from the mutant *Calm1* allele were the least abundantly expressed *Calm* transcripts in both hetero- and homozygous mutant hearts, while mutant hearts accumulated high levels of N98S-CALM protein in a *Calm1^N98S^* allele dosage-dependent manner, exceeding those of wild-type CALM protein. We further show that the severity of the electrophysiological phenotype incrementally increased with the graded increase in the mutant/wild-type CALM protein expression ratio seen in homozygous versus heterozygous mutant mice. We finally show a decrease in N98S-CALM protein degradation, suggesting that mutant CALM stabilization contributed to its enrichment in the heart. Our results support what we believe to be a novel mechanism by which a mutation in a single *Calm* gene can give rise to a severe phenotype.

## Abstract 2

The manuscript reports a new mechanim by which a human mutation in one ouf of 3 calmodulin genes can give rise to a severe cardiac phenotype.

## Graphical abstract

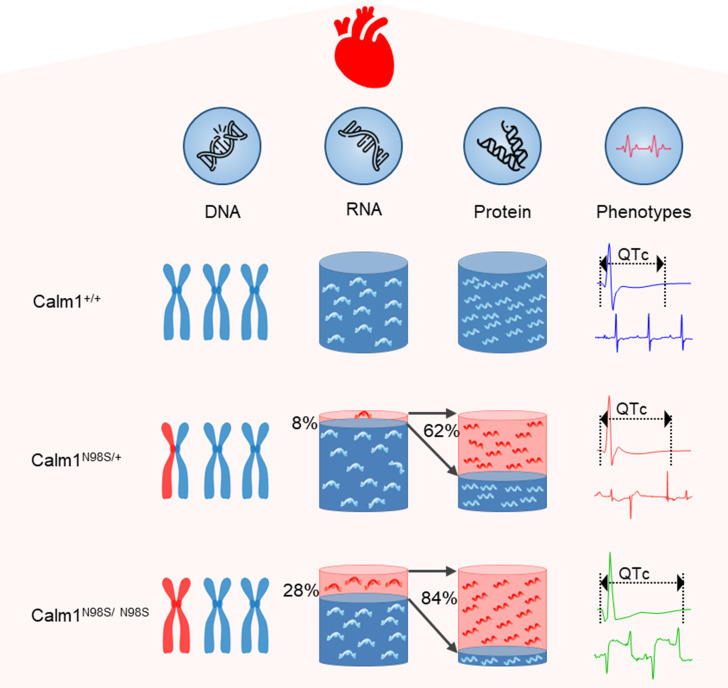

## Introduction

Missense mutations in calmodulin (CALM) can cause ventricular arrhythmia manifesting as long QT syndrome (LQTS), catecholaminergic polymorphic ventricular tachycardia (CPVT), and idiopathic ventricular fibrillation (1). CALM is a ubiquitously expressed Ca^2+^-binding protein, which decodes Ca^2+^ signals into downstream responses by undergoing conformational changes that in turn promote its binding to target proteins (2). There are 3 distinct *CALM* genes, *CALM1* (chr14q31), *CALM2* (chr2p21), and *CALM3* (chr19q13), that encode for completely identical 149–amino acid CALM proteins, though their coding sequences differ, predominately due to variability of the third base of codons (3). In all reported cases of arrhythmia-associated calmodulinopathies, the mutation occurs heterozygously in 1 of these 3 seemingly redundant *Calm* genes; i.e., only 1 out of 6 alleles harbors the mutation. The relative expression of *CALM1*, *CALM2*, and *CALM3* in adult human heart was reported, by quantitative PCR (qPCR) analyses, to be approximately 14%, 25%, and 61%, respectively (4). If one assumes that these numbers reflect the actual protein levels contributed by the translation of *CALM1*, *CALM2*, and *CALM3* mRNA, and wild-type and mutant alleles are transcribed at equal levels, relative abundances of mutant CALM proteins ranging from 7% to 31% should suffice to cause severe arrhythmias. Previous cell-based in vitro approaches have indeed demonstrated that low percentage abundances of certain LQTS- or CPVT-linked CALM mutants relative to those of wild-type CALM proteins were able to induce distinct phenotypes, suggesting a potent dominant-negative effect of mutant CALM molecules on their target proteins (5, 6). However, whether similarly low abundances of mutant CALM proteins also occur in intact hearts of heterozygous carriers remains to be determined. Here, we sought to directly quantitate mutant versus wild-type *Calm* transcript and protein levels in hearts of knockin mice harboring the p.N98S mutation in the *Calm1* gene (7, 8). Heterozygous mice exhibit a complex arrhythmia syndrome characterized by sinus bradycardia, adrenergic induced LQTS, and CPVT, mimicking the human calmodulinopathy associated with this mutation (1, 7, 8). Using RNA-sequencing and liquid chromatography-tandem mass spectrometry (LC-MS/MS) concurrently, we show that the transcripts from the mutant *Calm1* allele are the least abundantly expressed *Calm* transcripts in both heterozygous and homozygous mutant hearts, while, quite unexpectedly, mutant hearts accumulate high levels of N98S-CALM protein in a *Calm1^N98S^* allele dosage-dependent manner, exceeding those of wild-type CALM protein by ~1.5- to ~4-fold in heterozygous and homozygous hearts, respectively. We further show that phenotype severity incrementally increases with the graded increase in the mutant/wild-type CALM protein expression ratio seen in homozygous versus heterozygous mutant mice. We finally show a decrease in N98S-CALM protein degradation, suggesting that mutant CALM stabilization contributed to its excess accumulation in the heart. Previous in vitro studies have invoked high dominant-negative potency of pathogenic CALM mutants to explain the occurrence of severe arrhythmia even at very low molar ratios for mutant to wild-type CALM protein (5, 6, 9, 10). In contrast, our in vivo study reveals a preponderance of the mutant versus wild-type CALM protein in hearts from mice heterozygous for the *Calm1^N98S^* allele, suggesting that a high mutant–to–wild-type CALM ratio counterbalances the relatively low potency of N98S-CALM in inducing an arrhythmia phenotype. Overall, our results further help comprehend the complexity of calmodulinopathy mechanisms and develop targeted therapies.

## Results

**Figure 1:**
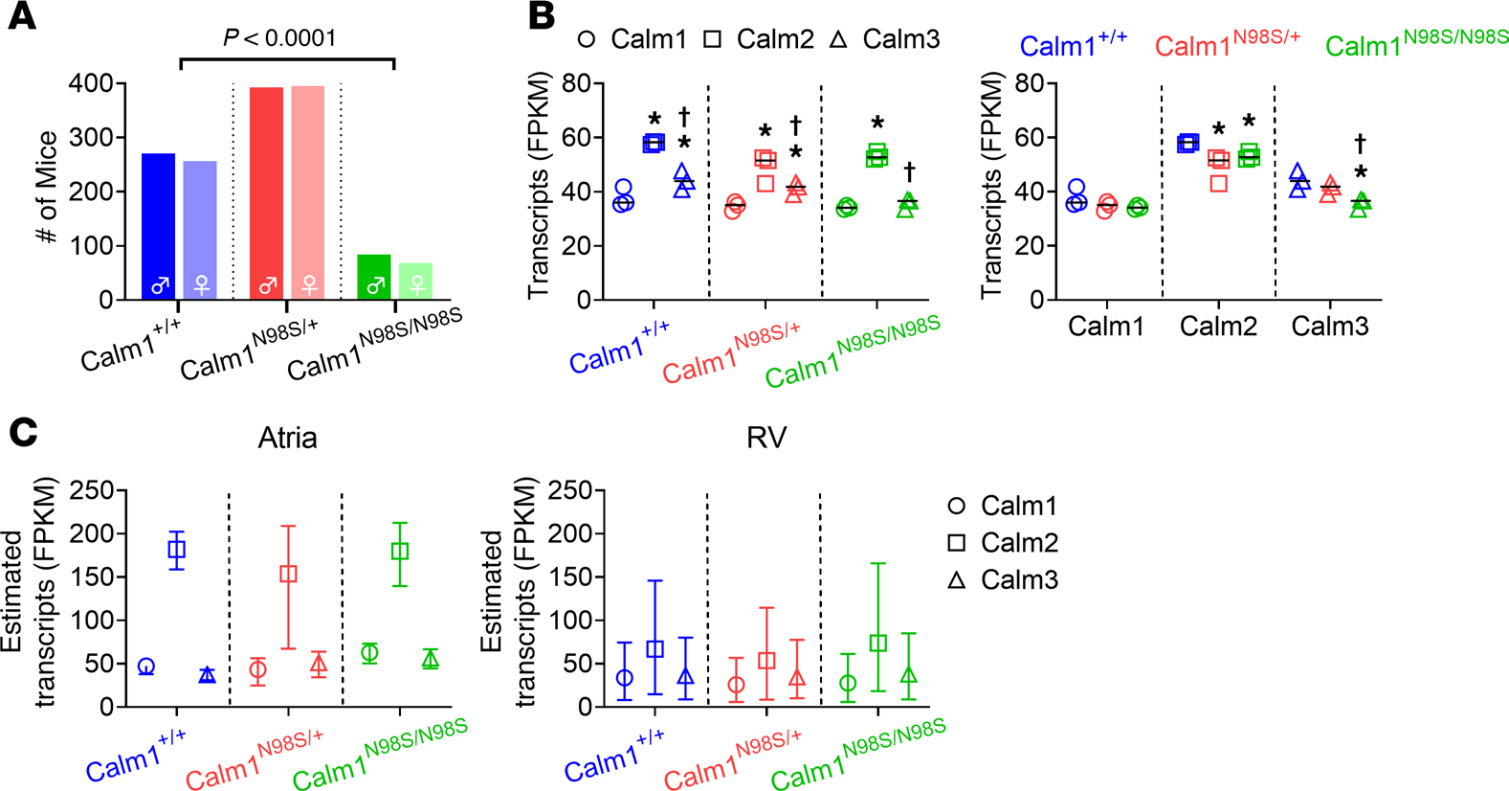
Genotype frequencies and *Calm* gene expression in adult offspring derived from *Calm1^N98S/+^* intercrosses. (**A**) Genotypes of postnatal day 10 (P10) offspring derived from *Calm1^N98S/+^* intercrosses. We used χ^2^ analysis for genotypic ratios. Observed genotypic proportions significantly deviate from those expected for Mendelian inheritance (*P* < 0.0001), irrespective of sex. (**B**) RNA-Seq was performed and the expression levels of the 3 *Calm* genes were quantitated in male hearts (left ventricles, LVs) from *Calm1^+/+^*, *Calm1^N98S/+^*, and *Calm1^N98S/N98S^* littermate mice. Data are displayed as scatter dot plots with the lines representing the medians from 3 biological replicates per genotype. A linear model of log(expression) as explained by genotype, *Calm* gene expression type, and the interaction of the two was used to compare mean *Calm* gene expression within each genotype (left) and among genotypes (right). *P ≤ 0.009 vs. *Calm1*, ^†^*P* ≤ 0.003 vs. *Calm2* in left panel; *P ≤ 0.043 vs. *Calm1^+/+^*, ^†^*P* ≤ 0.02 vs. *Calm1^N98S/+^* in right panel. FPKM, fragments per kilobase of transcript per million reads mapped. (**C**) RT-PCR–based estimates of absolute *Calm* gene transcript levels in atria and RV. Mean fold-changes in expression of each *Calm* gene in the atria and RV relative to their expression in LV were calculated by *2*^–ΔΔC^*T*, where ΔΔC_T_ = average(Δ*C_T,atria_* *_or_* *_RV,Calm_*) – average(Δ*C_T,LV,Calm_*), multiplied by the LV FPKM value for the corresponding *Calm* gene and genotype to obtain estimates of average absolute *Calm* transcript levels in atria and RV. Vertical bars denote error margins given by *2*^–ΔΔC^*T*^+SEM^ and *2*^–ΔΔC^*T*^–SEM^.

Mice heterozygous or homozygous for the p.N98S mutation in Calm1 (*Calm1^N98S/+^* and *Calm1^N98S/N98S^*, respectively) and their wild-type littermates (*Calm1^+/+^*) were generated by intercrossing *Calm1^N98S/+^* mice. Results of genotype analyses of P10 offspring are summarized in Figure 1A. Among a total of 1,465 pups (49.1% female), the observed frequencies of both *Calm1^N98S/+^* and *Calm1^N98S/N98S^* offspring were significantly lower than those expected for a 1:2:1 ratio among *Calm1^+/+^*, *Calm1^N98S/+^*, and *Calm1^N98S/N98S^* genotypes, with only ~75% and ~29%, respectively, of the expected number of *Calm1^N98S/+^* and *Calm1^N98S/N98S^* mice being alive. There were no significant differences in genotypic frequencies between male and female P10 mice (Figure 1A; *P* > 0.05 by Fisher’s exact test). The *Calm1^+/+^/Calm1^N98S/+^/Calm1^N98S/N98S^* genotypic ratio in embryos analyzed between E18 and E20 was found to be consistent with Mendelian inheritance (Supplemental Table 1; supplemental material available online with this article; https://doi.org/10.1172/jci.insight.185524DS1). The survival from P10 to P70 was significantly lower for *Calm1^N98S/N98S^* mice (91.4%) than for their *Calm1^N98S/+^* (99.0%) or *Calm1^+/+^* (99.7%) littermates (*P* < 0.0001 by Fisher’s exact test). These results suggest that the N98S variant resulted in late embryonic or early postnatal lethality, with the lethality being more pronounced in the homozygous versus heterozygous mutant mice, while long-term survival was impaired in homozygous mutant mice only. The survival of a portion of the homozygous mutant mice to adulthood opened the opportunity to assess gene dosage effects on phenotype.

### Calmodulin gene expression.

To assess the effect of hetero- and homozygosity for the *Calm1^N98S^* allele on *Calm* gene expression, we performed RNA sequencing (RNA-Seq) to quantify expression levels of *Calm1*, *Calm2*, and *Calm3* transcripts in male hearts (left ventricles, LVs) of *Calm1^+/+^*, *Calm1^N98S/+^*, and *Calm1^N98S/N98S^* littermate mice at 2 to 4 months of age. In wild-type tissue, mean RNA-Seq mRNA levels (scaled as FPKM, i.e., fragments per kilobase of transcript per million reads mapped) for *Calm2* were significantly higher compared with those for *Calm1* or *Calm3*, and mean transcript levels for *Calm3* were larger than those for *Calm1* (Figure 1B). These measurements yield average expressions from *Calm1*, *Calm2*, and *Calm3* in *Calm1^+/+^* LV of 27%, 41%, and 32%, respectively, which are in agreement with previously reported bulk RNA-Seq data for adult human heart (LV) (11) and mouse heart (10). In LVs of mutant mice, *Calm2* remained the most highly expressed *Calm* transcript, followed by *Calm3* and *Calm1*, respectively (Figure 1B). *Calm1^N98S/+^* mice are monoallelic for an A to G nucleotide substitution at position ch12:100206105 in exon 5 of *Calm1* (Supplemental Figure 1). This nucleotide change was used as a tag to estimate the frequencies of RNA-Seq mRNA reads that originate from each the mutant and the wild-type *Calm1* allele, by counting the number of mRNA-derived cDNA fragments containing the A or G nucleotide, respectively, at the ch12:100206105 position. The average count of nucleotide A–containing cDNA copies exceeded that of their nucleotide G–containing counterparts by 2.34-fold; i.e., the mutated allele is expressed at approximately 30% of the total *Calm1* transcript levels, which in turn amounts to approximately 8% of the total *Calm* transcripts in *Calm1^N98S/+^* hearts: 0.30 × FPKM_Calm1_ /(FPKM_Calm1_ + FPKM_Calm2_ + FPKM_Calm3_) = 0.30 × 34.8/(34.8 + 49 + 41.4) = 8.3%. In LVs of male *Calm1^+/+^* and *Calm1^N98S/N98S^* littermates, 100% of cDNA reads harbored an A or G nucleotide, respectively, at the ch12:100206105 position, as expected. Thus, the mutated allele accounts for approximately 28% (34.2 /(34.2 + 53.2 + 35.7 = 27.8%) of total *Calm* transcripts in LV of *Calm1^N98S/N98S^* male mice, which is almost identical to the 27% average expression from *Calm1* in *Calm1^+/+^* LV.

Results of the comparisons of left ventricular transcript levels for each of the 3 *Calm* genes among the 3 genotypes are summarized in the right panel of Figure 1B. Heterozygosity and homozygosity for the *Calm1^N98S^* allele modestly, yet significantly, reduced *Calm2* and *Calm3* transcript levels, respectively, but had no significant effect on *Calm1* transcript levels. Overall, these results indicate that the decreases in wild-type *Calm1* mRNA levels seen in mutant LVs did not lead to a compensatory upregulation of *Calm2* or *Calm3* gene expression.

While we did not directly measure absolute transcript levels of *Calm* genes in atria or right ventricle (RV), we leveraged a combination of quantitative reverse transcription PCR (RT-PCR) and the threshold cycle (*C_T_*) relative quantification (*2*^–ΔΔC^*T*) method (12) to be able to estimate cardiac chamber-specific *Calm* gene expression profiles. We reasoned that the product of mean fold-changes in atrial or right ventricular *Calm* gene expression relative to LV of the same genotype (*2*^–ΔΔC^*T*) and the corresponding LV FPKM value (obtained by RNA-Seq), *2*^–ΔΔC^*T* × FPKM, constitutes a good numerical proxy for absolute *Calm* gene transcript levels. RT-PCR–based estimates of FPKM values are listed in Supplemental Table 2. Figure 1C shows mean FPKM estimates for each *Calm* gene in atria and RV of wild-type and mutant mice. The results imply that mean mRNA levels for *Calm2* are higher than those for *Calm1* and *Calm3* in both RV and atria of all 3 genotypes, in concordance with our findings in the LV (Figure 1B), while *Calm1* and *Calm3* transcript levels are similar in both atria and RV within each genotype. The results further predict that average expression ratios of *Calm2* to *Calm1* (~3.4) or *Calm3* (~3.9) in the atria are larger than those in both RV (~2.3 and ~1.7) and LV (~1.5 and ~1.4). Overall, our numerical estimates suggest that *Calm2* is the most highly expressed of all 3 *Calm* genes within wild-type and mutant hearts, while chamber-specific differences in the magnitude of *Calm* gene expression ratios exist.

### CALM protein expression.

**Figure 2:**
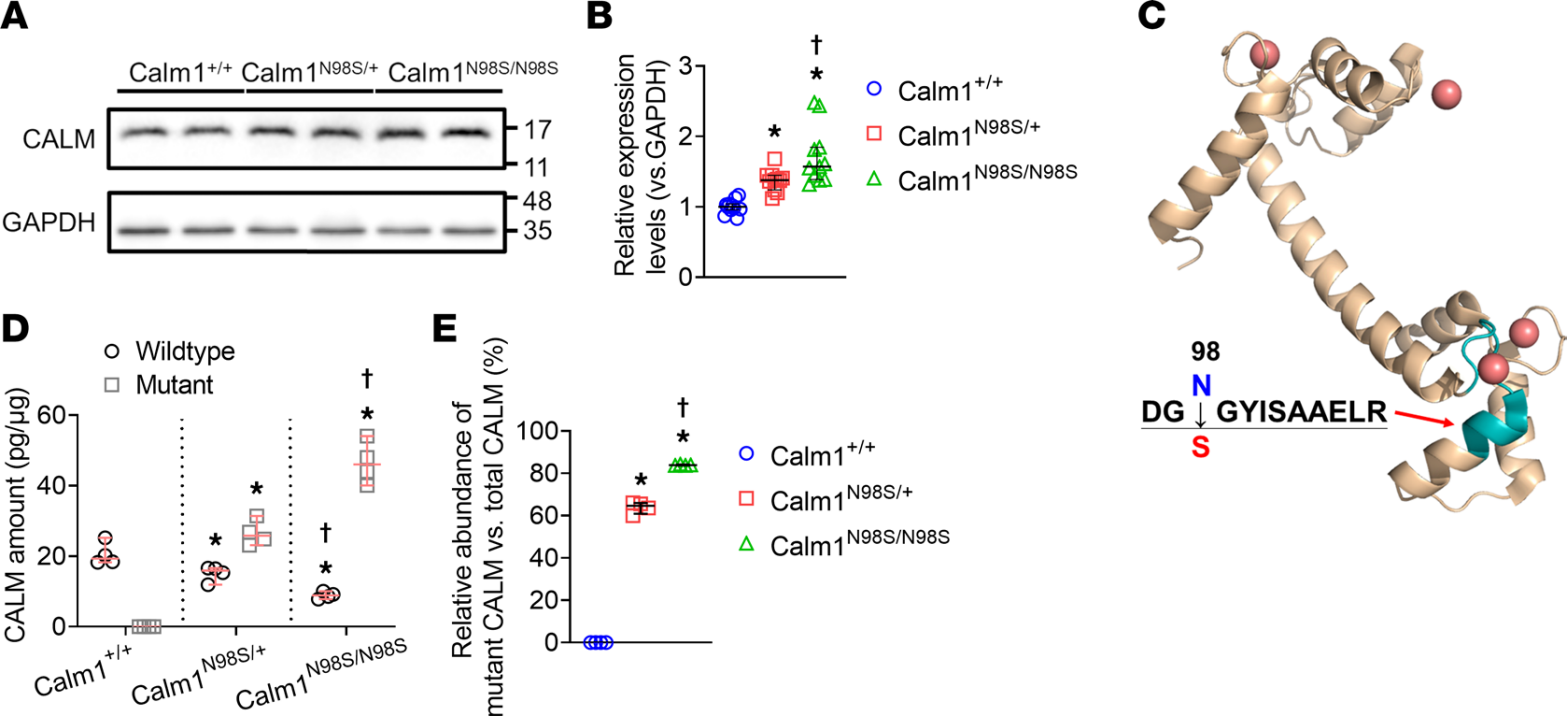
Assessment of total and mutant CALM protein expression. (**A**) Whole Western blot of lysates from LV of adult *Calm1^+/+^*, *Calm1^N98S/+^*, and *Calm1^N98S/N98S^* littermate mice probed with an antibody recognizing the C-terminal part of CALM (listed as binding within region aa 100). Values on the right are in kilodaltons. Samples were normalized to GAPDH protein expression. Densitometry with ImageJ (NIH) was used to calculate the intensity of the protein bands. (**B**) Normalized intensities of the CALM protein bands. Data are displayed as scatter dot plots with lines representing the median and interquartile ranges from 11 biological replicates per genotype. The Kruskal-Wallis test and the Wilcoxon rank sum test were used to compare normalized intensities among genotypes. *P ≤ 0.001 vs. *Calm1^+/+^*; ^†^*P* = 0.018 vs. *Calm1^N98S/+^*. (**C**) 3D model of Ca^2+^-bound CALM (Protein Data Bank code 1exr) highlighting the position of the amino acid sequence within the C-terminal region bracketing position 98 (green color). Shown are the amino acid compositions of the N98- and S98-harboring peptides that were obtained by enzymatic cleavage of wild-type or mutated CALM, respectively. Red spheres denote Ca^2+^ ions bound to EF-hand Ca^2+^-chelating loops within the C- and N-terminal lobes of CALM. (**D**) Wild-type and mutant CALM protein levels (normalized to the total protein amount per sample). *N* = 4 per genotype. (**E**) Relative abundance of mutant over total CALM protein in LV of adult *Calm1^+/+^*, *Calm1^N98S/+^*, and *Calm1^N98S/N98S^* male littermate mice. Data are displayed as scatter dot plots with horizontal lines denoting the median and interquartile ranges from 4 mice each per genotype. The Kruskal-Wallis test and the Wilcoxon rank sum test were used to compare CALM protein levels among genotypes. (**D** and **E**): *P = 0.030 vs. *Calm1^+/+^*; ^†^*P* = 0.030 vs. *Calm1^N98S/+^*.

Next, we sought to determine whether the mutated CALM causes changes in total CALM protein expression using Western blotting. Accordingly, lysates from LVs of adult *Calm1^+/+^*, *Calm1^N98S/+^*, and *Calm1^N98S/N98S^* male littermates were probed with an antibody recognizing the C-terminus of CALM. Representative results are shown in Figure 2A, revealing single CALM bands at the same expected molecular size in all 3 genotypes, indicating that the wild-type and mutant CALM protein have similar electrophoretic mobility. The normalized intensity of the CALM bands progressively increased from wild-type to *Calm1^N98S/+^* to *Calm1^N98S/N98S^* mice (Figure 2B). This result suggested augmented affinity of the mutated CALM to the antibody or increased total CALM protein expression in mutant hearts. To distinguish between these 2 possibilities, we next sought to quantitate total CALM protein levels as well as the relative abundances of wild-type and mutant CALM using LC-MS/MS.

We adapted a strategy for absolute quantification of wild-type and mutated CALM protein levels; left ventricular lysates from 4 adult mice per genotype were obtained for LC-MS/MS analysis. As shown in Figure 2C, profiling of enzymatically cleaved CALM peptides by LC-MS/MS verified that the asparagine-to-serine substitution at position 98 (N98S) was located between 2 cleavage sites. The synthetic peptide harboring a heavy leucine residue containing 1 ^15^N and 6 ^13^C isotopes produces a 7 Da mass shift of the peptides’ peaks in the mass spectrum (Supplemental Figure 2A). The isotope-labeled peptides were introduced into the samples as an internal standard, followed by the absolute quantification of wild-type and mutant peptides by LC-MS/MS (Supplemental Figure 2, B–D).

Median left ventricular levels of wild-type and mutant CALM protein progressively decreased and increased, respectively, from *Calm1^+/+^* to *Calm1^N98S/+^* to *Calm1^N98S/N98S^* male mice (Figure 2D). Increases in mutant protein levels more than outnumbered the decreases in wild-type levels, giving rise to elevations of total CALM levels in mutant hearts above those measured in *Calm1^+/+^* hearts. In *Calm1^N98S/+^* and *Calm1^N98S/N98S^* LVs, the median mutant CALM protein contents accounted for 61.8% (range: 59.9% to 66.1%) and 83.7% (range: 83.6% to 84.3%), respectively, of the total CALM protein content (Figure 2E), indicating that the p.N98S isoform was the predominant CALM isoform in mutants. Since *Calm1^N98S^* mRNA levels account for only ~8% and ~28% of the total *Calm* transcripts in *Calm1^N98S/+^* and *Calm1^N98S/N98S^* LV, respectively, the pronounced imbalance in the abundance of CALM proteins expressed from mutant versus the wild-type allele is unlikely to result from differences in *Calm1* transcript levels, invoking the existence of an alternative amplification mechanism underlying the mutant predominance.

**Figure 3:**
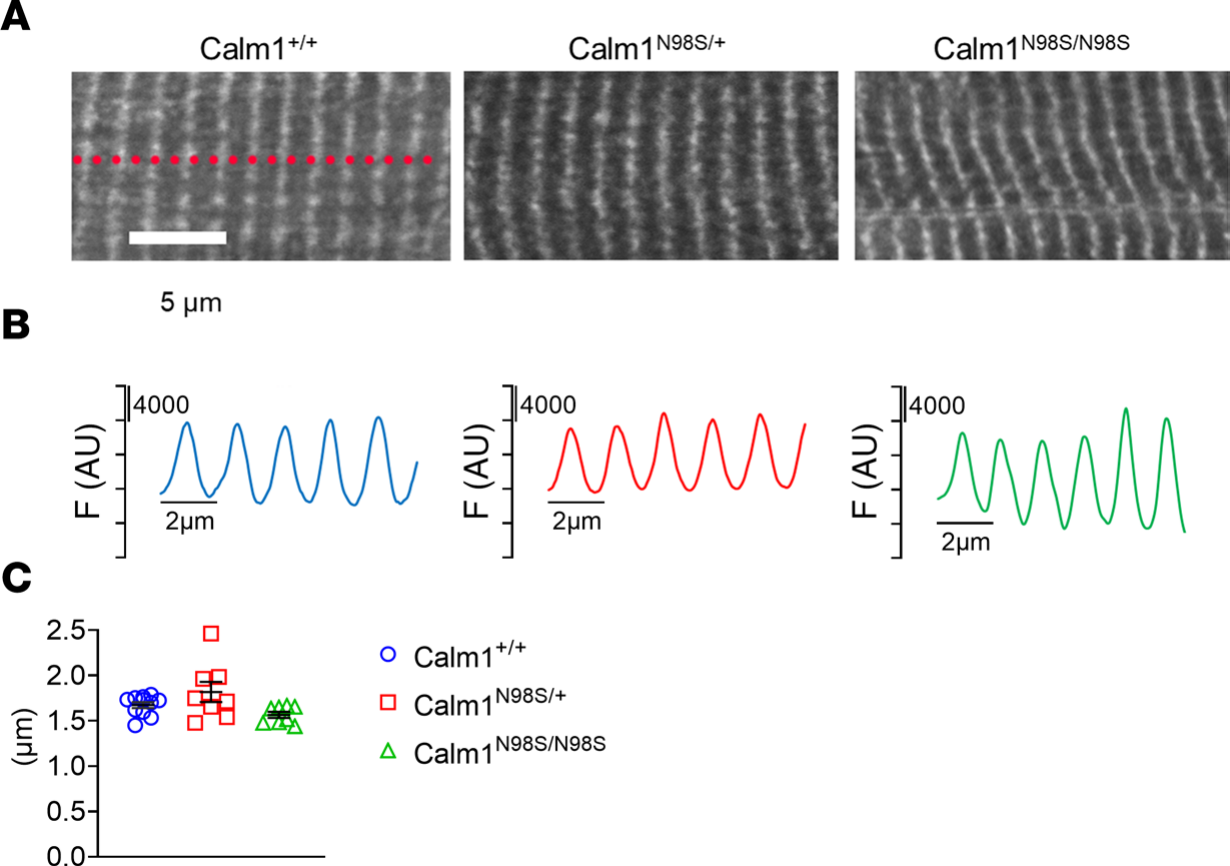
Intracellular distribution of CALM. (**A**) Representative confocal images of anti-CALM immunofluorescence in cryosections (10 mm) of adult *Calm1^+/+^*, *Calm1^N98S/+^*, and *Calm1^N98S/N98S^* hearts. Scale bar, 5 mm. (**B**) Plots of fluorescence signal intensity as a function of longitudinal distance. Lines were manually drawn perpendicular to the high-intensity fluorescence bands (see dotted line in left panel in **A**). Intensity profiles were generated by averaging the fluorescence intensity values of each 50-pixel high column along the lines from left to right. F, fluorescence intensity; AU, arbitrary units. (**C**) Dot plots of longitudinal distance between striations. Horizontal lines denote medians and interquartile ranges. *Calm1^+/+^*: 11 cells, *Calm1^N98S/+^*: 8 cells, *Calm1^N98S/N98S^*: 8 cells. There were no statistically significant differences among the 3 genotypes (*P* > 0.05 by Kruskal-Wallis 1-way analysis of variance on ranks).

Confocal fluorescence imaging of heart sections reacted with the same anti-CALM antibody that was used for Western blotting revealed a similar striated fluorescence pattern in left ventricular cardiomyocytes of all 3 genotypes (Figure 3A). The average longitudinal distance between striations was similar among the genotypes (Figure 3, B and C). The striated pattern is in agreement with that previously measured in isolated rodent and rabbit ventricular cardiomyocytes and is compatible with CALM enrichment along *Z*-lines (13, 14).

Median levels of wild-type and mutant CALM proteins were lower in right versus left ventricles of the same hearts (Supplemental Figure 3A and Figure 2D). The reciprocal changes in wild-type versus mutant CALM protein levels seen in LV were similarly observed in RV, with the mutant protein being the predominant isoform in mutant hearts.

Approximately one-fourth of *Calm1^N98S/+^* mice died perinatally. LC-MS/MS–based analyses of lysates from male neonatal hearts revealed gradual decreases in wild-type CALM protein content from *Calm1^+/+^* to *Calm1^N98S/+^* to *Calm1^N98S/N98S^* neonatal mice (Supplemental Figure 3B), which were more than offset by concomitant rises in mutant CALM protein levels, resulting in total CALM protein levels exceeding those measured in *Calm1^+/+^* neonatal hearts (Supplemental Figure 3B). Like in adult ventricles, the mutant CALM protein was found to be the most abundant isoform in neonatal *Calm1^N98S/N98S^* hearts (median 62.9%), whereas it accounted for 38.5% of the total CALM protein content in *Calm1^N98S/+^* neonatal hearts (Supplemental Figure 3B), compared with 56.2% and 63.8% in adult RV and LV, respectively (Supplemental Figure 3A and Figure 2E).

One possible mechanism explaining the imbalance in the abundance of CALM proteins expressed from mutant versus wild-type allele is a dysregulation of CALM protein turnover. Accordingly, we next sought to assess CALM protein degradation.

### Calm protein degradation.

**Figure 4:**
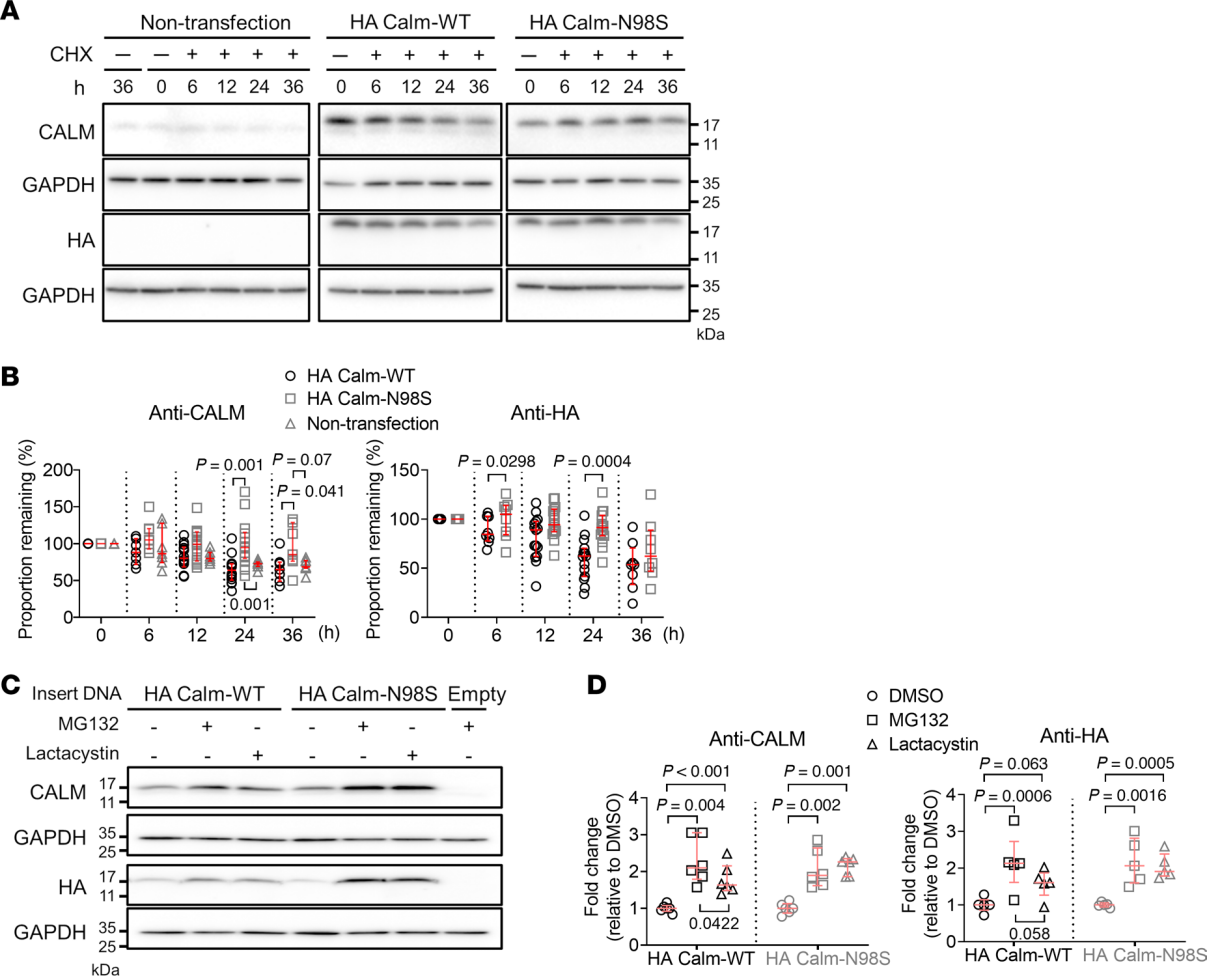
Quantification of CALM protein degradation. (**A**) Western blots of lysates from nontransfected HEK293T cells and HEK293T cells transfected with a vector encoding HA-tagged wild-type (HA CALM-WT) or mutant CALM (HA CALM-N98S). Cells were harvested before and after 6-hour, 12-hour, 24-hour, or 36-hour cycloheximide (CHX; 200 mg/mL) treatment. Control cells (labeled -) were harvested 36 hours after addition of the solvent (ethanol) only. Lysates were probed with an antibody recognizing the C-terminus of CALM or the HA-tag fused to the N-terminus of CALM. Samples were normalized to GAPDH protein expression. (**B**) Changes in normalized intensities of the endogenous CALM protein and HA-CALM protein bands expressed as percentages of their corresponding time 0 values and plotted as a function of CHX exposure time. Shown are dot plots with lines representing medians and interquartile ranges (*N* = 7–14 biological replicates per genotype). Each biological replicate is the average of 2 technical replicates. Numbers next to brackets denote *P* values by a repeated measures model with independent variables of plasmid, hour, and interaction of plasmid and hour. (**C**) Western blots of lysates from HEK293T cells transfected with HA CALM-WT, HA CALM-N98S, or control (labeled Empty) vectors. Lysates from cells following 24-hour exposure to MG132 (10 mmol/L) or lactacystin (10 mmol/L) were probed with the same anti-CALM or anti-HA antibodies as in **A**. Signal intensities were normalized to GAPDH protein expression. (**D**) Normalized intensities of HA-CALM protein bands expressed as fold-changes of normalized intensities in DMSO-treated cells. Lysates were probed with the anti-HA or anti-CALM antibody. Shown are dot plots with lines representing the median and interquartile ranges (*N* = 5 biological replicates per genotype per treatment). A linear model was used to compare mean CALM protein levels among treatment groups within the same genotype and treatment effects between genotypes.

In order to compare degradation of wild-type and mutant CALM protein, nontransfected HEK293T cells and HEK293T cells transfected with a plasmid encoding HA-tagged wild-type or mutant CALM were cultured in the presence of cycloheximide to inhibit translation. After treating cells with cycloheximide for 6, 12, 24, or 36 hours, we analyzed cell lysates by Western blotting. In parallel, lysates were probed with an antibody recognizing the C-terminus of CALM or the HA-tag fused to the N-terminus of CALM. Representative blots are presented in Figure 4A. Blots of protein extracted from transfected cells and probed with the anti-CALM antibody revealed bands at 17 kDa corresponding to exogenous HA-tagged CALM as well as bands at a slightly lower molecular weight. The lower weight bands appeared at the same molecular weight as the bands detected in lysates of control and nontransfected cells that were probed with the anti-CALM antibody, indicating that they represent endogenous CALM protein. Analyses of lysates probed with the anti-HA antibody exhibited single bands at 17 kDa for transfected cells, reflecting exogenous HA-CALM chimers, while HA-CALM bands were absent in lysates of nontransfected cells. Plots of relative changes in normalized intensities of endogenous and HA-tagged CALM protein bands detected by the anti-CALM antibody as a function of cycloheximide exposure time (Figure 4B) revealed significantly larger decreases for wild-type versus mutant HA-CALM at the 24-hour and 36-hour time points. Changes in the intensity of endogenous CALM protein bands of nontransfected cell lysates tracked those in the intensity of HA Calm-WT bands, although the difference between HA Calm-N98S and endogenous CALM following 36-hour cycloheximide exposure was of borderline significance only (*P* = 0.07). Analyses of Western blots of cell lysates that were reacted with the anti-HA antibody (Figure 4B) validated differences between HA CALM-WT– and HA CALM-N98S–expressing cells for both magnitude and time course of band intensity changes seen with the anti-CALM antibody, with the sole exception of the 36-hour time point, which showed similar percentage degradation of both HA-tagged CALM isoforms. This finding contrasts with the 36-hour results obtained with the anti-CALM antibody, revealing significant differences between HA Calm-WT– and HA Calm-N98S-CALM–expressing cells. The reason for this apparent discrepancy is unclear. Since the use of either antibody yielded largely concordant results at all earlier time points examined, one can speculate that time-dependent modifications of mutant CALM protein lower its affinity to the anti-HA, but not anti-CALM, antibody, giving rise to falsely low HA CALM-N98S protein levels. Posttranslational modifications such as ubiquitination and phosphorylation may create steric hindrance that specifically blocks access to the HA-tagged epitope. During prolonged culture conditions, the mutant CALM protein may also undergo conformational changes that differentially affect epitope accessibility. Finally, selective proteolytic cleavage events may occur near the HA-tag region, generating truncated proteins that retain the C-terminal CALM epitope but lose the N-terminal HA-tag. These considerations notwithstanding, our results suggest decreased degradation of mutant N98S-CALM protein in the experimental conditions employed here, resulting in an extension of its lifespan. On the other hand, 24-hour treatment with lactacystin or MG-132 to repress proteasome-mediated protein breakdown led to significant increases of both HA Calm-WT and HA Calm-N98S expression in HEK293T cells (Figure 4, C and D). These changes were indistinguishable in magnitude and occurred irrespective of the type of inhibitor used. Overall, these results are compatible with the notion that excess mutant CALM protein accumulation in mutant hearts results, at least partially, from attenuated degradation.

### Calm1^N98S^ allele burden determines cardiac phenotype in mice.

Mean body weight and mean heart weight/body weight ratios were significantly smaller and larger, respectively, in male *Calm1^N98S/N98S^* mice compared with both *Calm1^+/+^* and *Calm1^N98S/+^* mice, while heart weight/tibia length ratios showed no significant differences among genotypes (Supplemental Figure 4A). These results suggest that the larger heart weight–to–body weight ratio of homozygous mutant mice results from a lower body weight rather than from cardiac hypertrophy, in accordance with lack of echocardiographic evidence for myocardial hypertrophy (Supplemental Table 3). We did not detect increased fibrosis in mutant hearts (Supplemental Figure 4B). We performed echocardiography in 2D and M-mode to evaluate cardiac morphology and function. Representative M-mode images for each genotype are shown in Supplemental Figure 4C. Left ventricular systolic wall thickness, fractional shortening, and ejection fraction were found to be significantly decreased, while left ventricular end-systolic internal diameter and end-systolic volume were found to be significantly increased in *Calm1^N98S/N98S^* mice compared with both *Calm1^+/+^* and *Calm1^N98S/+^* mice (Supplemental Figure 4D and Supplemental Table 3). No differences in any echocardiographic parameter were seen between *Calm1^+/+^* and *Calm1^N98S/+^* littermates (Supplemental Table 3). The ability to exercise was significantly reduced in *Calm1^N98S/N98S^* versus both *Calm1^+/+^* and *Calm1^N98S/+^* mice but was retained in *Calm1^N98S/+^* mice (Supplemental Figure 4E). These results indicate that homozygosity, but not heterozygosity, for the *Calm1^N98S^* allele causes cardiomyopathy with contractile dysfunction of the LV.

**Figure 5:**
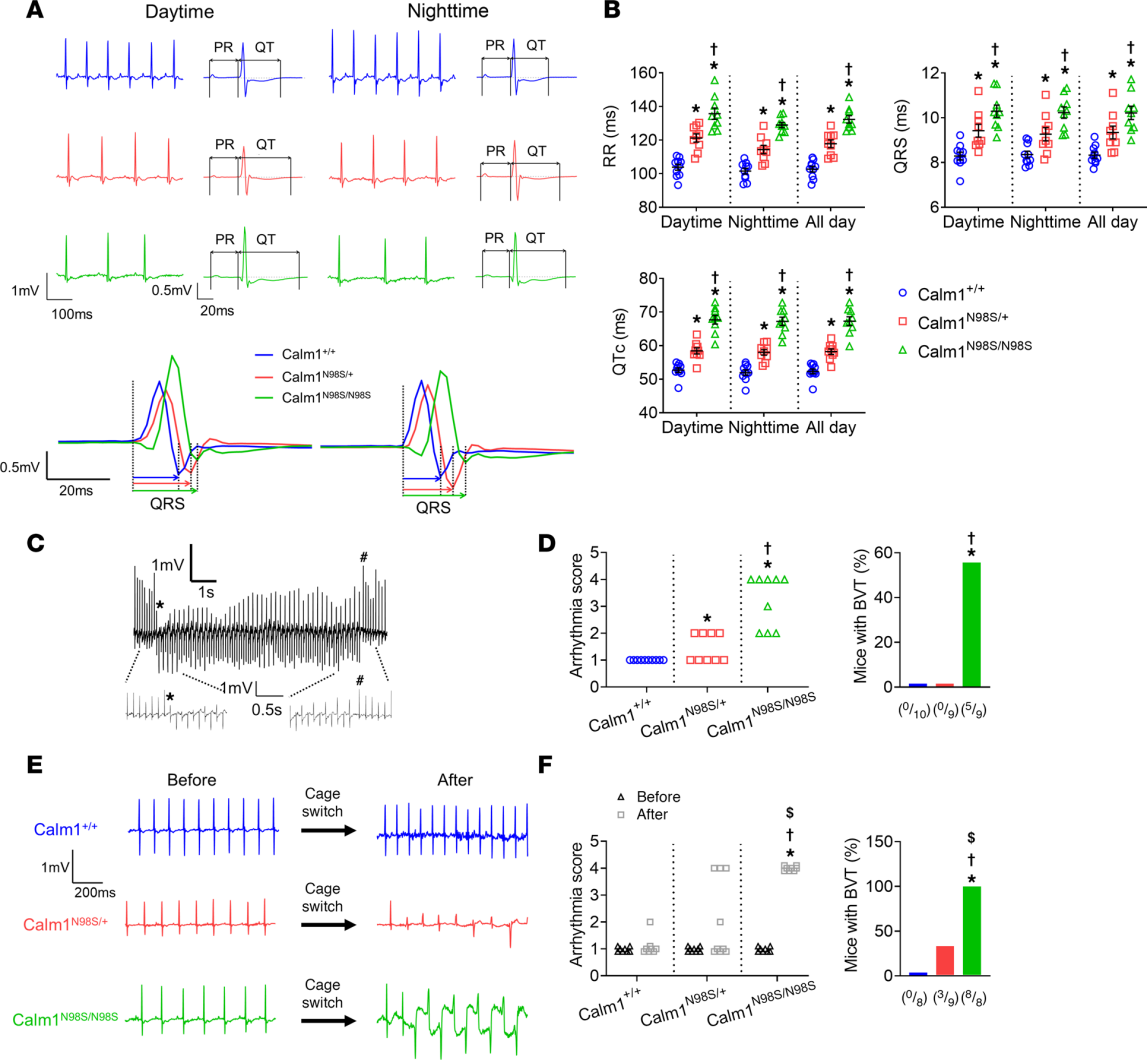
Dosage of *Calm1^N98S^* allele determines electrical phenotype in mice. (**A**) Left: Representative telemetered ECG traces recorded in ambulatory male mice. Right: Signal-averaged ECGs obtained by overlaying all consecutive cycles recorded within 5- to 10-second intervals, using QRS maximum or minimum for alignment. Lower rows show superimposed QRS complexes at expanded time scales. (**B**) Scatterplots of RR, QRS, and QT_c_ intervals. Each dot represents the 12-hour (dark or light cycle) or 24-hour (all day) average in a single animal. Data are from 10 *Calm1^+/+^*, 9 *Calm1^N98S/+^*, and 9 *Calm1^N98S/N98S^* mice. All populations were normally distributed. Horizontal lines superimposed on dots depict mean and SEM. *P ≤ 0.0118 vs. *Calm1^+/+^*; ^†^*P* ≤ 0.0144 vs. *Calm1^N98S/+^*. ECG measures were modeled by a repeated measures linear model with terms for genotype, time period, and the genotype by time period interaction. An unstructured covariance matrix was used. (**C**) Example of a spontaneous occurrence of a BVT in an ambulatory *Calm1^N98S/N98S^* mouse. (**D**) Ventricular arrhythmia scores in and percentage of ambulatory mice presenting with at least 1 episode of nonsustained (duration > 3 and < 15 s) or sustained (duration > 15 s) BVT within 24 hours of continuous ECG recording. *P ≤ 0.0325 vs. *Calm1^+/+^*; ^†^*P* ≤ 0.0294 vs. *Calm1^N98S/+^*. *P* values by Fisher’s exact and χ^2^ tests. (**E**) Examples of stress-induced electrocardiographic responses in ambulatory mice. (**F**) Ventricular arrhythmia scores and percentage of ambulatory mice developing BVT within 1 hour following a cage switch. Data are from 8 *Calm1^+/+^*, 9 *Calm1^N98S/+^*, and 8 *Calm1^N98S/N98S^* mice. *P = 0.0002 vs. *Calm1^+/+^*; ^†^*P* ≤ 0.016 vs. *Calm1^N98S/+^*; ^$^*P* = 0.0078 vs. before cage switch. *P* values for comparisons of proportions after cage switch between genotypes by Fisher’s exact test. *P* values for change in proportions by the Sign test.

To determine the gene dosage effects on basal electrocardiographic parameters and arrhythmia events, we performed 24-hour telemetric ECG monitoring in ambulatory mice. Representative 0.5-second ECG excerpts for each genotype are shown in Figure 5A. Mean 24-hour RR, QRS, and QT_C_ intervals gradually increased from *Calm1^+/+^* to *Calm1^N98S/+^* to *Calm1^N98S/N98S^* mice (Figure 5B and Supplemental Figure 5A), indicating that the extent of sinus bradycardia, intraventricular conduction deceleration, and ventricular repolarization delay seen in mutant mice aligned with the proportion of *Calm1^N98S^* alleles. Five out of 9 *Calm1^N98S/N98S^* mice but none of 10 *Calm1^+/+^* or 9 *Calm1^N98S/+^* mice developed at least one episode of bidirectional ventricular tachycardia (BVT) during 24 hours of continuous ECG recording (Figure 5, C and D). Major arrhythmia events in human carriers of *CALM* gene mutations are typically triggered by sympathetic stimulation (1). To examine whether arrhythmias can be induced by increasing sympathetic tone in our mutant mice, we recorded the heart rhythm in telemetered mice before and following a cage switch (15). While a cage switch significantly reduced average RR interval compared with baseline in all genotypes, RR interval differences between genotypes remained after the switch (Supplemental Figure 5B). The same maneuver significantly shortened QT_C_ interval in *Calm1*^+/+^ mice but significantly prolonged it in mutant mice (Supplemental Figure 5B). Cage switch triggered episodes of BVT in 9/9 homozygous and 3/9 heterozygous mutant mice but in none of the wild-type littermates (Figure 5, E and F). Similarly, treadmill exercise significantly increased both the arrhythmia score and the prevalence of inducible BVT in *Calm1^N98S/N98S^* mice but had no significant effect on arrhythmia score or incidence in heterozygous mutant or wild-type mice (Supplemental Figure 5, C and D). Overall, these results suggest a strong correlation between arrhythmia severity and the proportion of *Calm1^N98S^* mutant alleles.

**Figure 6:**
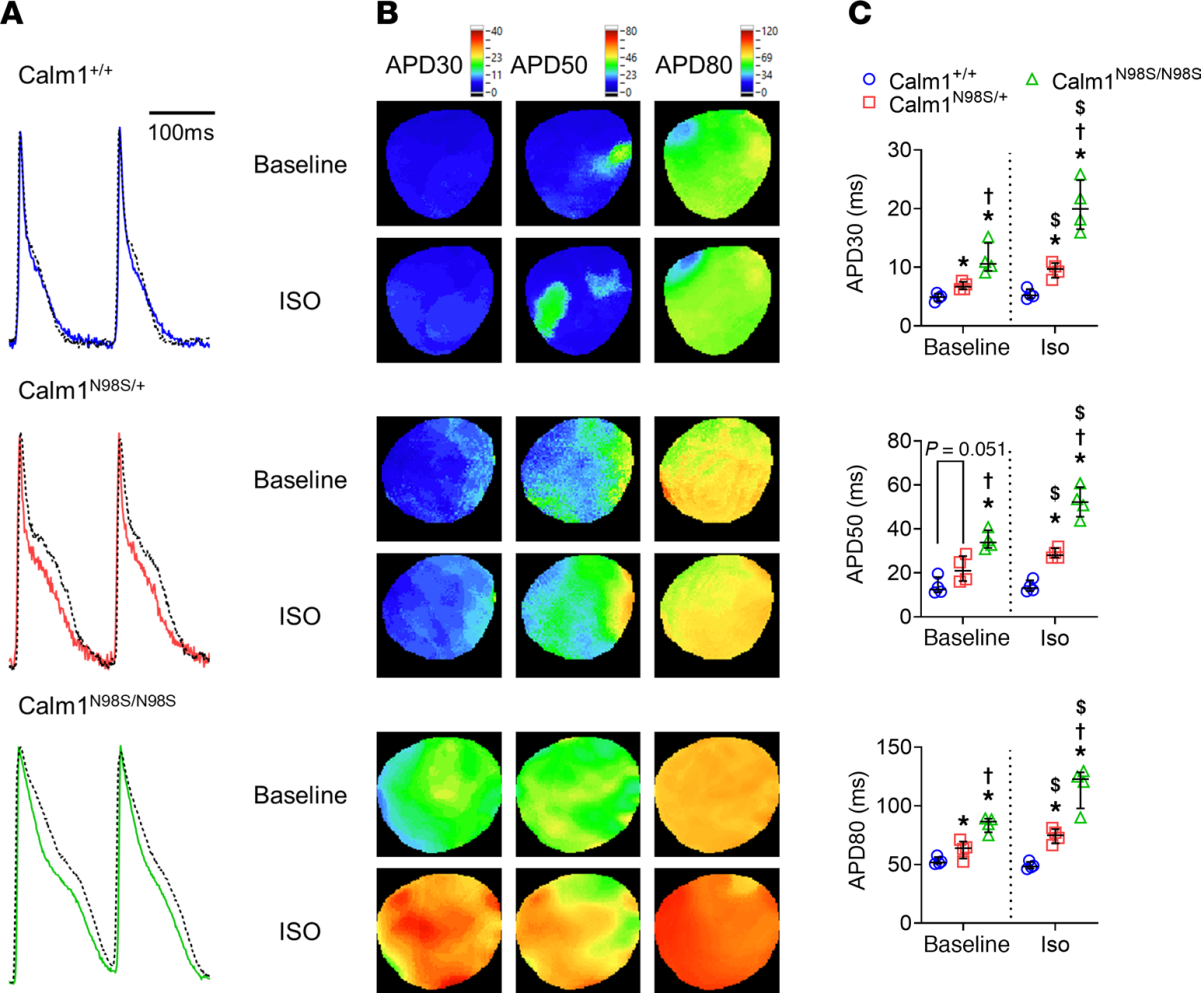
Magnitude of APD prolongation correlates with number of *Calm1^N98S^* alleles. (**A**) Superimposed representative optical action potentials recorded from anterior epicardium of LVs during atrial pacing at a cycle length of 120 ms before (solid lines) and during (dashed lines) isoproterenol (iso; 100 nM) treatment. (**B**) Exemplary maps of APD at 30%, 50%, and 80% repolarization (APD_30_, APD_50_, and APD_80_) in the absence and presence of isoproterenol. Colored scale bars are shown on the top of maps. (**C**) Dot plots showing APD_30_, APD_50_, and APD_80_. Horizontal lines superimposed on dots denote medians and interquartile ranges. *P ≤ 0.046 vs. *Calm1^+/+^*; ^†^*P* ≤ 0.004 vs. *Calm1^N98S/+^*; ^$^*P* ≤ 0.049 vs. before isoproterenol in the same genotype. *P* values by repeated measures linear model with independent terms for genotype, time period, and the genotype by time period interaction. An unstructured covariance matrix was used. The model assumptions of normality and homogeneity of variance of error terms were evaluated by residual plots. Since homogeneity of variance appeared to be violated, the data were transformed by a square root transformation.

We next performed fluorescence (“optical”) voltage mapping of Langendorff-perfused mouse hearts to determine whether N98S *Calm* allele dosage-dependent QT_C_ interval prolongations are mirrored by those in ventricular action potential duration (APD). All hearts were paced at a right atrial site at the same cycle length of 120 ms to avoid confounding effects of variable heart rates on ventricular repolarization duration. Typical optical action potentials and repolarization time isochron maps recorded from the anterior left ventricular epicardium of wild-type and mutant hearts at baseline and in the presence of the nonselective β-adrenergic receptor agonist isoproterenol are shown in Figure 6, A and B. The wild-type hearts had shorter epicardial APD at baseline and a blunted response to isoproterenol compared with the mutant hearts. Population data shown in Figure 6C demonstrate that APD_30_, APD_50_, and APD_80_ gradually increased from wild-type to heterozygous to homozygous hearts in the absence and presence of isoproterenol and that the magnitude of isoproterenol-induced APD prolongation was larger in *Calm1^N98S/N98S^* versus *Calm1^N98S/+^* hearts. Isoproterenol had no significant effect on APD in wild-type hearts. These results suggest that (a) delays in ventricular repolarization underlie the long QT phenotype seen in mutant mice, (b) the extent of APD prolongation both at baseline and during isoproterenol correlates with the proportion of mutant alleles, and (c) N98S-CALM potentiates the APD response to β-adrenergic receptor stimulation.

Moreover, we analyzed epicardial conduction patterns and velocities to assess whether conduction defects in the ventricular myocardium contribute to Calm1^N98S^ allele dosage-dependent QRS widening. Supplemental Figure 6A shows exemplary epicardial activation maps from the anterior surface of a *Calm1^+/+^*, *Calm1^N98S/+^*, and *Calm1^N98S/N98S^* heart during ventricular pacing at a cycle length of 120 ms, revealing an anisotropic conduction pattern in all 3 genotypes, enabling measurements of maximal and minimal conduction velocity (CV_max_ and CV_min_, respectively). Supplemental Figure 6B shows mean CV_max_ and CV_min_ for all hearts tested. Both CV_max_ and CV_min_ were significantly reduced in homozygous mutant hearts compared with wild-type and heterozygous mutant hearts, whereas no statistical differences were found between *Calm1^+/+^* and *Calm1^N98S/+^* hearts. These findings suggest that impairment of impulse propagation within the ventricular myocardium contributes to QRS widening observed in *Calm1^N98S/N98S^* mice.

**Figure 7:**
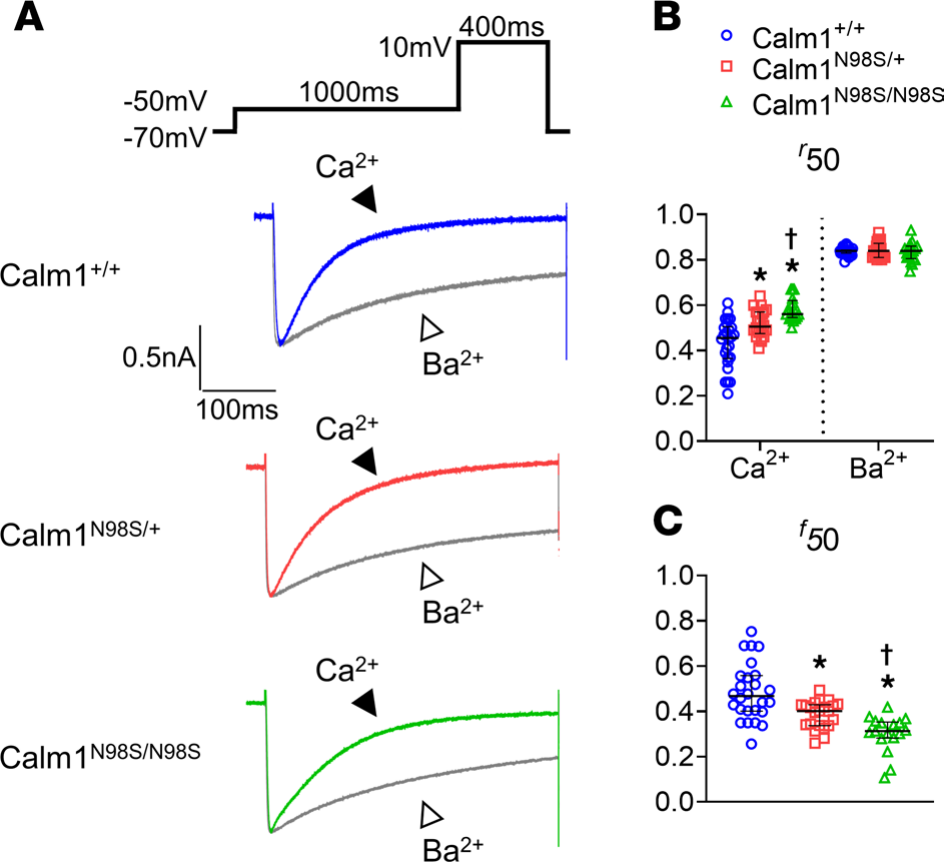
Progressive CDI decrement of cardiac I_Ca.L_ with increase in *Calm1^N98S^* allele number. (**A**) Exemplar traces of whole-cell L-type Ca^2+^ current (I_Ca.L_) recorded in left ventricular cardiomyocytes in response to 400 ms step depolarizations to +10 mV. Traces were normalized to their respective peaks to facilitate comparison of decay kinetics. Pipette solution contained 5 mM ryanodine and 10 mM BAPTA to restrict Ca^2+^ elevations to those in the nanodomains of individual L-type channels (6). CDI is evident as the accelerated decay in Ca^2+^ current as compared with Ba^2+^ in all 3 genotypes. Ca^2+^ currents exhibit progressively slower decays from *Calm1^+/+^* to *Calm1^N98S/+^* to *Calm1^N98S/N98S^* cardiomyocytes, while decay kinetics of Ba^2+^ currents are almost indistinguishable among genotypes. (**B** and **C**) Scatter dot plots of the fraction of peak I_Ca.L_ remaining at 50 ms after the peak (*r_50_*) using Ca^2+^ versus Ba^2+^ as charge carriers (**B**) and dot plots of *f_50_* = (*r_50,Ca_* – *r_50,Ba_*)/*r_50,Ba_*, a measure of CDI (**C**). Horizontal lines superimposed on dots represent the median and interquartile ranges; *n* = 21 cells from *N* = 4 mice (*Calm1^+/+^*); *n* = 16, *N* = 5 (*Calm1^N98S/+^*); *n* = 14, *N* = 4 (*Calm1^N98S/N98S^*); *P ≤ 0.001 vs. *Calm1^+/+^*; ^†^*P* = 0.004 vs. *Calm1^N98S/+^*. *P* values from a repeated measures linear model with independent terms of genotype. A compound symmetric covariance matrix was used, which assumes equal correlation from repeated measurements of the same experimental unit.

We and others have previously demonstrated that N98S-CALM attenuates Ca^2+^/CaM-dependent inactivation (CDI) of cardiac L-type current (6, 7). This attenuation adds Ca^2+^ entry during the plateau of the action potential, prolonging its duration as compared with cardiomyocytes expressing wild-type CALM. If reduced CDI underlies APD prolongation seen in mutant hearts (Figure 6), then one would expect an inverse relationship between the magnitude of CDI and the proportion of mutant *Calm1* alleles. Accordingly, we next assessed CDI of whole-cell L-type Ca^2+^ currents by applying the patch-clamp technique to single left ventricular myocytes isolated from adult male wild-type and mutant hearts. Ca^2+^ currents exhibited progressively slower decays in response to a step depolarization to +10 mV as the number of mutant alleles increased (Figure 7A). To dissect the contributions of CDI and voltage-dependent inactivation (VDI) to the current decay, Ba^2+^, which binds only poorly to CALM, was used as the charge carrier to measure the extent of VDI within the same cell. CDI was evident as the excess inactivation of the Ca^2+^ trace (Figure 7A). Substitution of Ca^2+^ with Ba^2+^ abrogated differences in inactivation kinetics between genotypes, validating their Ca^2+^ dependence (Figure 7, A and B). Median values for *f_50_*, a measure of CDI, were decreased by 23.1% and 42.3% in *Calm1^N98S/+^* and *Calm1^N98S/N98S^* ventricular myocytes, respectively, compared with *Calm1^+/+^* cells (Figure 7C), indicating progressive attenuation of CDI from *Calm1^+/+^* to *Calm1^N98S/+^* to *Calm1^N98S/N98S^* cardiomyocytes. Incremental decreases in CDI were associated with progressive prolongation of both APD and QT_C_ (Figures 5 and 6). These results support the notion that the mutant *Calm1* allele dosage regulates the extent of CDI reduction and further suggest a role of impaired CDI in determining the severity of the long QT phenotype.

### Transcriptomic and proteomic profiling.

Transcriptomic and proteomic analyses were performed on LV samples from the same group of *Calm1^+/+^*, *Calm1^N98S/+^*, and *Calm1^N98S/N98S^* male littermates (*N* = 3 per genotype) to examine molecular changes related to excitation-contraction coupling, adrenergic signaling, and contractility. RNA-Seq and LC-MS/MS analyses revealed no significant differences in expression levels of proteins and their corresponding mRNA for calcium-handling proteins, sarcomeric contractile proteins, or β-adrenergic signaling components (Supplemental Figure 7), suggesting that these canonical pathways remain largely unaffected at the transcriptional and translational levels.

An unbiased, label-free proteomic analysis using LC-MS/MS identified 1,787 proteins. Among these, 65 were significantly differentially expressed proteins (DEPs) each in *Calm1^+/+^* versus *Calm1^N98S/+^* or *Calm1^+/+^* versus *Calm1^N98S/N98S^* hearts, based on an absolute log_2_FC > 0.5 and *P* < 0.05. Compared with *Calm1^+/+^* hearts, *Calm1^N98S/+^* hearts exhibited 39 upregulated and 26 downregulated DEPs, while *Calm1^N98S/N98S^* hearts showed 45 upregulated and 20 downregulated DEPs. Protein-protein interaction analysis using the STRING database (https://string-db.org) (16), combined with functional enrichment and *k*-means clustering, revealed distinct genotype-dependent biological effects. In *Calm1^N98S/+^* hearts, DEPs were predominantly associated with mitochondrial translation and cellular component assembly, with key network clusters involving mitochondrial translation and type I hemidesmosome assembly — both essential for energy production and tissue integrity (Supplemental Figures 8 and 9). In contrast, *Calm1^N98S/N98S^* hearts showed enrichment in pathways related to muscle structure development, muscle cell differentiation, chromatin and nucleosome assembly, and muscle cell development, with major network clusters centered on the large ribosomal subunit and fascia adherens (Supplemental Figures 10 and 11). While the former plays a crucial role in protein synthesis and muscle function, the latter is a specialized cell-cell junction in cardiac muscle, essential for heart contraction. Thus, the heterozygosity and homozygosity of the *Calm1^N98S^* allele had distinct effects on biological processes by altering protein-protein interactions, ultimately contributing to the observed interactome changes. Heterozygosity for the *Calm1^N98S^* allele primarily disrupts cellular energy balance and tissue integrity, while homozygosity induces broader effects on muscle development, potentially contributing to reduced left ventricular systolic function and impaired exercise endurance observed in *Calm1^N98S/N98S^* mice. Therefore, while primary cardiac regulatory pathways remain unchanged, secondary proteomic alterations may significantly contribute to the functional deficits observed in mutant hearts.

## Discussion

### Mechanisms underlying predominance of N98S-CALM protein in mutant hearts.

Parallel analyses by Western blotting and LC-MS/MS document a progressive increase in cardiac CALM protein content as the proportion of mutant *Calm1^N98S^* allele increases. LC-MS/MS further revealed that the rises in total CALM protein content solely resulted from those in mutant isoform content, since wild-type CALM protein levels gradually decreased as *Calm1^N98S^* allele dosage increased. In hetero- and homozygous mutants, the relative contributions of mutated CALM protein to total protein were ~62% and ~84%, respectively, while relative mRNA expression from *Calm1^N98S^* accounted for only 8% and 28%, indicating markedly higher protein/mRNA ratios for the mutant versus wild-type allele. Increases in protein/mRNA ratios can result from increased translational efficiency of *Calm1^N98S^* mRNA or reduced mutant CALM protein turnover. Our in vitro measurements support a role of a decrease in N98S-CALM protein degradation in mediating mutant predominance. Whether other *Calm* mutations similarly give rise to excess mutant protein expression in vivo and whether a reduction in protein lifespan contributes to the mutant predominance remain to be investigated. Limpitikul et al. found no change in total CALM protein levels in human induced pluripotent stem cell–derived cardiomyocytes heterozygous for the D130G mutation in *CALM2* (9), whereas Rocchetti et al. reported an increase in total CALM level of borderline significance in human induced pluripotent stem cell–derived cardiomyocytes harboring the F142L mutation in *CALM1* (17). Western blot analyses of lysates from P19 cells homozygous for the same mutation revealed that the mutated CALM is expressed at approximately 30% of the total CALM protein (10), which matches the average expression from *Calm2* in adult mouse heart of 32%. Thus, these findings suggest that the increase in total CALM protein expression and the predominance of the mutant CALM isoform seen in our mouse model are unique and may not extend to other *Calm* mutations.

Our in vitro data demonstrate that proteasome activity regulates CALM protein breakdown. Ziegenhagen and coworkers demonstrated multiple ubiquitination of calmodulin in reticulocyte lysate (18). Another study demonstrated degradation of oxidized calmodulin by the 20S proteasome (19). Vice versa, it is well established that CALM regulates the ubiquitin-proteasome system to control protein degradation (20, 21). Our proteomics data are compatible with similar expression levels for proteins other than CALM in wild-type and mutant hearts, suggesting that N98S-CALM, rather than modulating proteasome-mediated protein breakdown in general, selectively catalyzes its own ubiquination or deubiquination. However, the sample sizes for proteomics analyses are too small to draw a definite conclusion. It also remains to be tested whether in addition to altered proteasome-mediated CALM protein degradation, changes in other degradative systems (e.g., lysosomal autophagy) contribute to predominance of the mutant CALM isoform in our model.

Although levels of HA-tagged wild-type and mutant CALM similarly increased in the presence of proteasome inhibitors, suggesting comparable efficiency of plasmid-derived mutant and wild-type mRNA in initiating HA-CALM protein translation, the possibility that enhanced translational efficiency of the mutant versus wild-type allele contributes to the high protein-to-mRNA ratio for *Calm1^N98S^* in mutant hearts cannot be discarded. In this context, Munk et al. recently predicted that differences in translational efficiency among the 3 *Calm* genes due to divergent codon usage could play a regulatory role as the 3 genes differ markedly in their use of synonymous codons (11). Thus, the nucleotide substitution in codon 78 of *Calm1* might impinge on the relative efficiency of *Calm1^WT^* versus *Calm1^N98S^* mRNA translation.

The mechanism underlying the significant reduction in wild-type CALM protein levels in the mutant hearts remains to be elucidated. These changes may reflect those in wild-type *Calm* transcript levels or increased wild-type CALM protein breakdown.

### Calm1^N98S^ allele dosage and phenotypic trait.

In cases of arrhythmia-associated calmodulinopathy, the mutation occurs heterozygously in 1 out of 3 seemingly redundant *Calm* genes, i.e., with only 1 out of 6 alleles harboring the mutation. Expression from *Calm1* was reported, by qPCR analyses, to be 14% in adult human heart (11). Assuming that this number reflects the actual CALM protein level contributed by the translation of *Calm1* mRNA, and the wild-type and mutant *Calm1* alleles are transcribed at equal levels, it had been predicted that a ratio of approximately 0.1 for mutant to wild-type CALM protein expression would be sufficient to cause a severe phenotype (5, 6, 9). Indeed, it was shown previously that dialysis of isolated, genetically naive ventricular cardiomyocytes with a solution supplemented with recombinant wild-type and N98S-CALM proteins at an 8:1 ratio was sufficient to induce arrhythmogenic Ca^2+^-handling aberrancies via augmenting ryanodine receptor activity (5). Limpitikul and coworkers previously demonstrated that a ~5:1 expression ratio for wild-type CALM to D96V CALM molecules yielded a ~25% decrement of CDI of L-type channels expressed in HEK293 cells, sufficient to appreciably prolong APD (6). For a similar extent of CDI reduction (23%) in *Calm1^N98S/+^* myocytes, we found a median value of 0.62 for the wild-type to mutant CALM expression ratio, and a median expression ratio of 0.2 seen in *Calm1^N98S/N98S^* myocytes was associated with a 42% CDI decrement (Figure 2F and Figure 7C), compared with approximately 90% for the D96 CALM mutant Limpitikul et al. reported (6). Like D96V CALM, small amounts of 2 additional CALM variants (D130G and F142L) were shown to bestow similarly strong dominant-negative effects on CDI of L-type channels (6), suggesting lower potency of the N98S versus D96V (D130G, F142L) CALM in attenuating CDI. Differences in inhibitory potency can result from several mechanisms. First and second, the effective [Ca^2+^] required to elicit half of the maximal CDI (*EC_50_*) can vary among CALM mutations (6, 22), and so can the CALM concentration required to obtain the *EC_50_*. Third, the ability of the CALM interaction to elicit enhancement of CDI may be impaired. Last, the rates of Ca^2+^ or CALM complex formation or conformational change mediating CDI could be altered. Low efficacy of N98S-CALM may shield the CDI machinery from arrhythmogenic effects resulting from small variations in mutant CALM protein levels.

*Calm1^N98S^* allele dosage-dependent changes in APD and QT_C_ interval duration correlated with those in CDI reduction, compatible with a causal role of the latter in mediating the long QT component seen in our mouse model. CDI attenuation has been implicated as a major mechanism underlying LQTS associated with a variety of CALM mutations. Our findings as well as those by others suggest the expression ratio of mutant to wild-type CALM molecules as a major determinant of phenotypical severity. However, besides changes in the wild-type to mutant ratio, we also observed gradual decreases and increases, respectively, of wild-type and total cardiac CALM protein levels as *Calm1^N98S^* allele dosage increased, making it difficult to unambiguously identify the factor(s) that control(s) phenotypic expression. Excess accumulation of low-Ca^2+^-affinity N98S-CALM concomitant with wild-type CALM depletion could considerably alter the Ca^2+^-buffering properties of myocytes in a concentration-dependent manner, specifically in regions of CALM protein enrichment (23), which in turn would significantly modulate the activity of Ca^2+^-regulated proteins. Also, CALM deficiency alone could alter the activity of those of its binding partners that do not or only very poorly bind N98S-CALM.

We did not see a correlation between *Calm1^N98S^* allele dosage and changes in left ventricular chamber geometry or systolic function. Rather, structural and functional remodeling was restricted to homozygous mice, suggesting the possibility that the mechanisms underlying cardiomyopathy in *Calm1^N98S/N98S^* mice required higher mutant or lower wild-type CALM protein levels than those mediating the electrophysiological phenotype. Intriguingly, our proteomic analyses indicate that homozygosity for the *Calm1^N98S^* allele impacts cardiac energy synthesis and morphogenesis, potentially contributing to the poor left ventricular function, reduced endurance, and perinatal lethality observed in *Calm1^N98S/N98S^* mice. Transgenic overexpression of wild-type CALM in the mouse heart caused left ventricular hypertrophy and systolic dysfunction via overactivation of the calcineurin pathway (24). Mice homozygous for the p.P1124L mutation in the *RyR2* gene developed left ventricular hypertrophy, which was mediated by cardiac CALM overexpression (25). Intriguingly, a 17-month-old boy with cardiac arrest and left ventricular hypertrophy reportedly harbored a p.N98I mutation in *Calm2* (26), but no information on cardiac CALM protein levels was provided. Overall, CALM appears to act as an in vivo regulator of cardiac growth, and excess accumulation of mutant N98S protein may have contributed to the cardiac remodeling seen in homozygous mutant mice. The threshold for changes in mutant or wild-type CALM protein levels that are necessary to induce a cardiomyopathy phenotype is higher than that for LQTS manifestation.

Homozygous mutant hearts exhibit pro-arrhythmic conduction slowing within the ventricular myocardium, suggesting impairment of voltage-gated, fast Na^+^ currents, though our previous measurements in *Calm1^N98S/+^* ventricular myocytes did not reveal Na^+^ current abnormalities (7). Thus, the mechanism underlying slowed conduction in *Calm1^N98S/N98S^* hearts remains to be determined.

Our numerical analyses predict that average *Calm2* transcript levels in the atria exceed those in the ventricles, suggesting the possibility that mutations in *Calm2* are more likely to give rise to an atrial phenotype and/or cause more severe atrial phenotypes than those in *Calm1* and *Calm3*. While sinus bradycardia has been documented in a number of patients carrying single mutant alleles in any of the 3 *Calm* genes (27), *Calm* mutations/atrial phenotype linkages have not been systematically studied to date.

Calmodulinopathy is frequently associated with extracardiac manifestations, e.g., neurological disorders (1, 27), which may contribute to perinatal mortality of the homozygous mutant mice. The reduced body weight seen in adult *Calm1^N98S/N98S^* mice further suggests that CALM function is critical for body weight homeostasis. Clearly, studies are warranted to elucidate the mechanisms underlying noncardiac manifestations of CALM mutations.

### Therapeutic implications.

Of the 140 patients in the International Calmodulinopathy Registry (1), 11 patients (7.8%) from 11 unrelated families are heterozygous for the p.N98S mutation in *Calm1*, making it the third most frequent mutation of the registry, following the p.N54I (14%) and p.N138K (14%) mutations in *Calm1* and *Calm3*, respectively. Therefore, our findings have important therapeutic implications for a substantive fraction of patients with calmodulinopathy. Our parallel measurements of cardiac *Calm1* transcript and CALM protein levels revealed that a less than 10% contribution from *Calm1* was sufficient to give rise to excess levels of the mutant protein in hearts from heterozygous mutant mice. Antisense oligonucleotide–mediated *Calm1* depletion has recently been demonstrated to suppress CPVT in *Calm1^N98S/+^* mice (28). Notably, successful therapy of *Calm1^N98S/+^* mice required more than 85% depletion of *Calm1* transcripts, confirming our observation that even low amounts of remaining *Calm1^N98S^* mRNA can give rise to disease-evoking N98S-CALM protein levels. It remains to be seen whether other *Calm* mutations require transcript reductions of similar magnitude to achieve therapeutic efficacy. CRISPR/Cas-mediated *Calm* gene ablation is an alternative therapeutic strategy (9). For either therapeutic approach, however, it will be important to ensure that levels of wild-type CALM protein remain largely unaffected to avoid undesired effects arising from CALM protein deficiency. Finally, promoting mutant CALM degradation may constitute an alternative approach for calmodulinopathy treatment.

### Conclusion.

Our results suggest that *Calm1^N98S^* mRNA levels are an inadequate proxy for N98S-CALM protein levels in the heart. Whether low abundances of transcript levels for other human CALM mutants can be similarly offset by changes to regulation of translation and/or protein degradation remains to be determined in future studies. The findings reveal the benefit of measuring mutant CALM mRNA and protein levels concurrently to disentangle complex calmodulinopathy mechanisms.

## Methods

### Sex as a biological variable.

Our study exclusively examined male mice. It is unknown whether the findings are relevant for female mice. However, we previously reported indistinguishable phenotypes of adult female and male knockin mice heterozygous for the human p.N98S mutation in the *Calm1* gene (7). We thus expect the findings of the present study to be equally relevant for female mutant mice.

### Mice.

We previously reported the generation of knockin mice heterozygous for the p.N98S mutation in *Calm1* (abbreviated *Calm1^N98S/+^*) using CRISPR/Cas9 genome editing (7). *Calm1^N98S/+^* mice harbor an AT to GC nucleotide substitution in codon 98, resulting in an asparagine (AAT) to serine (AGC) amino acid substitution and concomitantly introducing a *BsrB1* restriction site (CCGCTC). In the present study, *Calm1^N98S/+^* mice were intercrossed, genomic DNA was extracted from digit biopsies of P10 offspring, and the genotypes were determined using a PCR-based restriction fragment length polymorphism assay (Supplemental Figure 1). Mice were housed in a specific pathogen–free barrier facility that maintained a 12-hour light/12-hour dark cycle. All mice had access to food and water ad libitum. For all experiments, only male mice at 2 to 6 months of age were used. For harvesting hearts for histology, RNA-Seq, proteomics, optical mapping, and myocyte isolation, mice were euthanized by cervical dislocation.

### Mouse echocardiography.

2D and M-mode echocardiography were performed as described previously (7). In brief, transthoracic echocardiography was performed in isoflurane-anesthetized (administered from nose cones at a concentration of 1.5%) adult mice using a Prosect T1 ultrasonograph equipped with a 30–50 MHz linear-array probe (Scintica Instrumentation, Inc.). Heart rate was monitored and recorded throughout the study. Left ventricular chamber dimensions and wall thickness during systole and diastole were obtained from M-mode tracings based on measurements averaged from at least 3 separate cardiac cycles. Left ventricular fractional shortening and ejection fraction were calculated using standard formulas.

### Histology.

Hearts were immersion-fixed with 4% formaldehyde for 24 hours at 4°C, cryoprotected with 30% sucrose in PBS for 24 hours, and embedded in OCT (Sakura). Ten-micrometer-thick longitudinal sections were cut on a cryotome (Leica 3050S). The sections were stained with hematoxylin and eosin (Sigma-Aldrich) or Masson’s trichrome (ScyTek Laboratories) and examined under a light microscope. Images were captured with a digital camera (DS126311, Canon) using a 4× objective. The extent of myocardial fibrosis was quantified from histological images using a color threshold method developed in ImageJ (NIH). Pixels representing fibrotic tissue were extracted from the difference image of the red and blue channels of the original image, which provided the best contrast between normal and fibrotic tissue. Total myocardial tissue was extracted from the grayscale image. The percentage of tissue fibrosis was calculated as the ratio of the pixel numbers representing fibrotic and total myocardial tissue, respectively.

### Immunohistochemistry and confocal imaging.

Ten-millimeter-thick sections from fixed and cryoprotected hearts were incubated with 0.2% Triton X-100 (Sigma-Aldrich) in PBS for 1 hour, followed by 30 minutes of blocking with 2% BSA. Sections were then incubated for 12 hours with a polyclonal rabbit anti-CALM antibody (1:200; ab45689; Abcam) in PBS supplemented with 2% BSA and 10% goat serum and subsequently reacted with an Alexa Fluor 555–conjugated goat anti-rabbit IgG antibody for 1.5 hours (1:100; A21429; Invitrogen). All incubation steps were performed at room temperature, and between all incubation steps, the slides were thoroughly washed with PBS 3 times for 5 minutes each. Sections were mounted in ProLong Gold solution (P36930; Invitrogen). Sections that had been incubated with secondary antibody without having been labeled with anti-CALM antibody served as control. CALM signal was detected at excitation wavelength of 555 nm and emission wavelength of 570–620 nm. Images were acquired using a 60× 1.4 NA oil-immersion objective. Images were taken at a zoom of 3 (pixel size, 69 nm; 1,024 × 1,024 pixels per image), with a 16-bit dynamic range. A Kalman filter of 3 was used. Pixel dwell time was set to 8 ms.

### RNA isolation and sequencing.

Total RNA was extracted from the left ventricular tissues of *Calm1*^+/+^, *Calm1*^N98S/+^, and *Calm1^N98S/N98S^* (*N* = 3 per genotype) with TRIzol (Thermo Fisher Scientific) using previously described methods (29, 30). RNA 6000 Nano Kit and 2100 Bioanalyzer (Agilent Technologies) were utilized to assess the quality and integrity of the total RNA samples. Only RNA samples with an RNA integrity number of at least 8 were used for subsequent library construction and sequencing. Mouse left ventricular RNA-Seq (1 μg total RNA for each sample) libraries were constructed using a SureSelect Strand-Specific RNA library prep kit (Agilent Technologies) according to the manufacturer’s instructions. Barcoded libraries were quantitated using Qubit 3.0 and pooled for sequencing on a NovaSeq 6000 sequencer (Illumina). After separation of the multiplexed sequencing data, adapter sequences were removed, and the individual libraries were converted to FASTQ format. Sequence read pairs were aligned to the mouse genome (Mm10) with Hisat, followed by processing and sorting with Samtools (30, 31). The expression level of individual mRNA was quantified by Partek Genomic Suite 6.0 using the RefSeq annotation database (32). The raw fragment counts of individual mRNAs were normalized to the transcript length (in kilobase) and total mapped fragment counts (in million fragments) in the same sample and expressed as FPKM. Sequence reads mapped to different isoforms of individual genes were pooled together for subsequent comparative analyses.

### Quantitative RT-PCR.

For *Calm* mRNA expression (allele nonspecific), total RNA was isolated from hearts of 2- to 4-month-old male *Calm1^+/+^* (*N* = 8), *Calm1^N98S/+^* (*N* = 7), and *Calm1^N98S/N98S^* (*N* = 7) animals using the Total RNA Purification Kit (GeneMark), and mRNA was reverse-transcribed to cDNA using the ReverTra Ace qPCR RT Kit (TOYOBO). The expression levels of the transcripts encoding *Calm1*, *Calm2*, and *Calm3* were determined by SYBR quantitative RT-PCR (LabStar) and QuantStudio 5 (Thermo Fisher Scientific) using sequence-specific primers (Supplemental Table 4). Each RT-PCR was performed using the following conditions: 30 seconds at 94°C, 30 seconds at 55°C, and 1 minute at 72°C. *C_T_*s and melting curve measurements were performed with ABI software. Data were analyzed using the *2*^–ΔΔC^*T* method (12) using glyceraldehyde-3-phosphate dehydrogenase (*Gapdh*) as the endogenous control. Specifically, *C_T_* values were calculated for *Calm1*, *Calm2*, *Calm3*, and *Gapdh*, and the Δ*C_T_* values (i.e., *C_T,Calm_* – *C_T,Gapdh_*) were determined. The mean fold-change in expression of each *Calm* gene in the atria and RV, normalized to *Gapdh* and relative to the expression of the same *Calm* gene in the LV, was calculated for each tissue type (atria, RV, and LV) using the formula *2*^–ΔΔC^*T*, where ΔΔC_T_(atria) = average(Δ*C_T,atria,Calm_*) – average(Δ*C_T,LV,Calm_*) and ΔΔC_T_(RV) = average(Δ*C_T,RV,Calm_*) – average(Δ*C_T,LV,Calm_*). The *2*^–ΔΔC^*T* values for each *Calm* gene in the atria and the RV of all 3 genotypes were then multiplied by the LV FPKM values for the corresponding *Calm* gene and genotype (obtained from RNA-Seq analyses of LV samples) to obtain estimates of absolute *Calm* gene transcript levels.

### Western blot analysis.

Left ventricles were lysed in RIPA buffer (50 mM Tris-HCl at pH 8.0, 150 mM NaCl, 0.5% sodium deoxycholate, 1% NP-40, and 0.1% SDS) supplemented with 1% protease inhibitor cocktail (Thermo Fisher Scientific) and homogenized on ice. The homogenate was centrifuged at 20,000*g* for 10 minutes at 4°C. The supernatant was collected and total protein concentration was detected by BCA protein assay kit (Thermo Fisher Scientific) using bovine serum albumin as standard. Protein samples were mixed with 4× sample buffer and denatured for 10 minutes, at 100°C. Protein samples were loaded onto 15% polyacrylamide gel in 10% SDS-PAGE in Ca^2+^-free conditions. Separated proteins were transferred from the gel onto a PVDF membrane. Thereafter, the membrane was blocked using 5% skim milk for 1 hour at room temperature before incubation with desired primary antibodies of appropriate dilution (rabbit polyclonal anti-HA antibody [ab45689, Abcam], rabbit monoclonal anti-calmodulin 1/2/3 antibody [catalog ab45689, Abcam], and rabbit monoclonal anti-actin antibody [Cell Signaling Technology, catalog 4970S]) at 4°C overnight. After three 10-minute washes with phosphate-buffered saline containing 0.1% Tween 20, the membrane was probed using species-specific secondary antibodies (goat anti-rabbit IgG conjugated to horseradish peroxidase, Merck Millipore, catalog AP132P) for 1 hour at room temperature. For detection of protein bands, the membrane was treated with enhanced chemiluminescence substrate (MilliporeSigma) for 5 minutes, and the signals were captured by UVP ChemStudio PLUS (Thermo Fisher Scientific). Intensities of the protein bands were measured using ImageJ software.

### Single-pot solid-phase-enhanced sample preparation and LC-MS/MS analysis.

Description of the methods used for sample preparation and mass spectrometry analyses are provided in Supplemental Methods.

### Telemetric ECG recording.

Telemetric ECG recording was performed as previously described (7). In brief, ECG telemetry devices (F10, Data Science International) were implanted intra-abdominally under isoflurane anesthesia, and recordings were initiated 2–3 days after recovery from surgery. Mice were exposed to 12-hour light/12-hour dark cycles (light, 6 am to 6 pm). Mean RR intervals were calculated from 5- to 10-second samples evaluated at 20-minute steps throughout the 24-hour recording periods. Signal-averaged ECGs were obtained from the same 10-second samples, by superimposing the QRS maximum or minimum as fiduciary points, and PR, QRS, and QT intervals were measured. The QTc interval was calculated according to the formula by Mitchell et al. (33). Values for the exponent *n* in the correction formula were obtained by linear regression of ln(QT) on ln(RR/RR_average_), where RR_average_ corresponds to the mean of the average 24-hour RR intervals in ambulatory mice. Correction factors were 0.29, 0.575, and 0.606 for *Calm1^+/+^*, *Calm1^N98S/+^*, and *Calm1^N98S/N98S^* male mice, respectively.

### Treadmill and cage switch.

After an initial 24-hour recording period, telemetered mice were trained on a single-lane graded treadmill for 10 minutes on 3 consecutive days. At the day of treadmill test, mice initially ran at a speed of 10 m/min and slope of 15 degrees, and speed was increased by 2 m/min every minute until the mice reached exhaustion. Exhaustion was defined as the inability of the mouse to run on the treadmill for 10 seconds despite electrical shock (34).

Cage switch was routinely begun at 10 am by removing ECG transmitter–carrying mice from their original cage and placing them into a cage previously occupied by a different male mouse for approximately 3 hours. Ventricular arrhythmia scores during the 1-hour interval preceding and following the cage switch were defined by the worst ventricular arrhythmia (1 – no or isolated PVCs, 2 – ventricular bigeminy and/or frequent PVCs >10/min, 3 – ventricular couplet, 4 – non-sustained ventricular tachycardia lasting ≥ 3 s and ≤ 15 s) (35).

### Epicardial optical voltage mapping of Langendorff-perfused hearts.

High-resolution optical mapping experiments were performed on hearts from 4- to 6-month-old mice as described previously (7). Details are provided in Supplemental Methods.

### Isolation of single ventricular myocytes.

Mouse left ventricular myocytes were isolated enzymatically by a well-established protocol (36). Details are provided in the Supplemental Methods.

### Cellular electrophysiology.

Whole-cell L-type Ca^2+^ currents were recorded in single left ventricular myocytes as described previously (7).

### Cell culture and transient transfection.

HEK293T cells were obtained from iCell Bioscience Inc. The cells were cultured in DMEM/High Glucose (HyClone) supplemented with 10% FBS (HyClone), 100 U/mL penicillin, and 100 ng/mL streptomycin (Gibco) and incubated in a humidified incubator (5% CO_2_, 37°C). For transfection, HEK293T cells were cultured in 35 mm cell culture dishes until the monolayers reached 70%–80% confluence. Mouse cDNA sequences encoding for CALM-WT or CALM-N98S were subcloned into pcDNA3.1-N-HA vector (Genomics Inc.). A total of 4 μg pcDNA3.1-N-HA-mCalm-WT, pcDNA3.1-N-HA-mCalm-N98S, or pcDNA3.1 vector (empty control vector) was diluted separately in 250 μL Opti-MEM (Thermo Fisher Scientific). Then, 12 μL TransIT-X2 Transfection Reagent (Mirus) was added and mixed by pipetting, followed by incubation for 30 minutes at room temperature. The mixtures were then added to HEK293T cell cultures. After 24 hours of incubation, the DMEM containing transfected cells was replaced with 2 mL fresh culture medium, and the cells were incubated for another 12 hours before cycloheximide (200 mg/mL dissolved in ethanol) was added to assess protein degradation. Cells were harvested for Western blot analyses immediately before and following 6-hour, 12-hour, 24-hour, or 36-hour treatment with cycloheximide. In other experiments, transfected HEK293T cells were exposed for 24 hours to the proteasome inhibitors MG132 (10 mmol/L in DMSO) or β-lactacystin (10 mmol/L in DMSO) to examine a role of altered mutant *Calm1* mRNA translation in regulating N98S-CALM protein levels. In all experiments, HEK293T cell cultures were transfected with a mixture of 2 mg pcDNA3.1-N-HA-mCalm-N98S or 2 mg pcDNA3.1-N-HA-mCalm-WT, along with 2 mg pcDNA3.1-N-HA per dish.

### Statistics.

A linear model of natural logarithm of expression as explained by genotype, *Calm* gene, and the interaction of the 2 was used to compare *Calm* gene expression within each genotype. Residual plots were examined to assess model assumptions of normality and homogeneity of error (Figure 1).

Nonparametric Kruskal-Wallis test was used to compare normalized intensities of the CALM protein bands, total CALM protein levels, and relative abundance of mutant CALM protein between the 3 genotype groups. The Wilcoxon rank sum test was used to conduct pairwise comparisons of groups (Figure 2).

A repeated measures model with independent terms for plasmid, time, and the interaction of plasmid and time with an unstructured covariance matrix was used to compare mean CALM protein as a function of cycloheximide exposure time (Figure 4B). To compare changes in HA-CALM protein as a function of treatment, we used a linear model with terms for plasmid (genotype), treatment, and the interaction of the two. To compare responses to treatment between genotypes, we used a linear model with single independent term of plasmid (genotype; Figure 4D). Body weight, heart weight–to–body weight ratio, heart weight to tibial length, fractional shortening, left ventricular ejection fraction, treadmill time to exhaustion, and echocardiographic data were also compared between genotypes by a similar linear model (Supplemental Figure 4 and Supplemental Table 3).

ECG measures were modeled by a repeated measures linear model with terms for genotype, time period, and the genotype by time period interaction. An unstructured covariance matrix was used (Figure 5 and Supplemental Figure 5). Proportions of arrhythmia scores and BVT incidence after cage switch were compared between genotypes by Fisher’s exact test. The change in proportion within genotype was tested by the Sign test (Figure 5). Similar tests were used to test scores and proportions after treadmill exercise (Supplemental Figure 5). APD was modeled by repeated measures linear model with independent terms for genotype and time period and the genotype by time period interaction. An unstructured covariance matrix was used. The model assumptions of normality and homogeneity of variance of error terms were evaluated by residual plots. Since homogeneity of variance appeared to be violated, the data were transformed by a square root transformation (Figure 6).

Values for *r_50_* and *f_50_* were analyzed using a repeated measures linear model with independent terms of genotype. A compound symmetric covariance matrix was used, which assumes equal correlation from repeated measurements of the same experimental unit (Figure 7).

Wilcoxon rank sum test was used to compare gene and protein expression between genotypes using RNA-Seq and LC/MS-MS, respectively (Figure 2 and Supplemental Figure 3).

A *P* value less than 0.05 was considered significant. SAS/STAT Software version 9.4 (SAS Institute Inc.) was used for analysis.

### Study approval.

The study was approved by the Institutional Animal Care and Use Committee (IACUC) of the Tzu Chi Hospital, Hualien, Taiwan. All procedures were in accordance with the ethical standards of the responsible committee (IACUC Approval No. 106-51) on humane experimentation according to *Guide for the Care and Use of Laboratory Animals* (National Academies Press, 2011).

### Data availability.

All raw data from this study can be viewed in the Supporting Data Values file. RNA-Seq data are made publicly available via National Center for Biotechnology Information Gene Expression Omnibus at https://www.ncbi.nlm.nih.gov/geo/query/acc.cgi?acc=GSE301709

## Author contributions

Wen-Chin Tsai designed and conducted experiments, analyzed data, provided reagents, and wrote the manuscript. CFY designed and conducted experiments and analyzed data. SY Liang and SY Lin designed studies, conducted experiments, and analyzed data. Wei-Chung Tsai designed studies, conducted experiments, and analyzed data. SG conducted experiments. XL and SO analyzed data. KCY designed experiments, conducted experiments, provided reagents, and analyzed data. TCM designed and conducted experiments and analyzed data. PSC wrote and revised the manuscript. MR designed research studies and wrote and revised the manuscript.

## Supplementary Material

Supplemental data

Unedited blot and gel images

Supporting data values

## Version 1 07/24/2025

In-Press Preview

## Version 2 09/09/2025

Electronic publication

## Version 3 05/27/2026

"Tzu Chi University" corrected to "Tzu Chi Hospital"

## Version 4 05/27/2026

Tzu Chi University" corrected to "Tzu Chi Hospital"
