# Supplementary material for: Enrichment of mutant calmodulin protein in a murine model of a human calmodulinopathy
Source: JCI Insight. 2025 Jul 24;10(17):e185524. doi: 10.1172/jci.insight.185524 (PMC12487685; doi:10.1172/jci.insight.185524)
Supplement: Supplemental data [file jciinsight-10-185524-s008.pdf]

Cardiac Enrichment of Mutant Calmodulin Protein in a Murine Model of a Human Calmodulinopathy

Short Title: Calmodulin expression in calmodulinopathy hearts

Wen-Chin Tsai[1,2], Chiu-Fen Yang[1, 3], Shu-Yu Lin[4], Suh-Yuen Liang[5], Wei–Chung Tsai[6], Shuai Guo[2], Xiaochun Li[7], Susan Ofner[7], Kai-Chien Yang[8,9], Tzu-Ching Meng[3], Peng-Sheng Chen[10], Michael Rubart[2]

Supplemental Material

Methods

Single-pot solid-phase-enhanced sample preparation (SP3)

Left ventricles from adult male *Calm1*$^{+/+}$, *Calm1*$^{N98S/+}$, and *Calm1*$^{N98S/N98S}$ littermates were gently homogenized in ice-cold RIPA buffer [250 mM Tris-Cl pH 7.4, 750 mM NaCl, 5% NP-40, 2.5% Na-deoxycholate and 0.5% SDS], containing 1x protease inhibitor cocktail and phosphatase inhibitors (2 mM Na_3VO_4, 10 mM NaF, and 10 mM $Na_2P_2O_7$), and centrifuged for removal of debris. Protein concentration was determined by Bradford assay. The samples were reduced with 10 mM dithioerythritol in 8 M urea, 25 mM ammonium bicarbonate, pH 8.5, at 37 ºC for 1 h, and subsequently alkylated with 25 mM iodoacetamide at room temperature in the dark for 1 h. Then the reaction was quenched with 25 mM dithioerythritol. Two types of carboxylate-modified paramagnetic beads (SpeedBead Magnetic Carboxylate Modified Particles [E3] & [E7], Cytiva) were combined in a ratio of 1:1 (v/v), rinsed and reconstituted in DI water at a concentration of 25 µg/µL. The SP3 beads mixture was added into the samples with 1:50 protein-to-beads ratio. Five hundred uL of 100% ethanol and 180 uL of 80% ethanol were used for binding and rinsing process. The beads were re-suspended in 40 uL of 25 mM ammonium bicarbonate after ethanol had been removed. The samples were digested with 1:50 enzyme to protein ratio of Mass Spectrometry Grade Lys-C (catalog # 125-05061, Wako) at 37 ºC for 3 hr. Following Lys-C digestion, same amount of sequencing grade trypsin (Promega) was added and digested at 37 ºC for 16 hr. The samples were separated from the beads and the digestion reaction was quenched with 0.1% formic acid then dried in SpeedVac. The peptide mixture was aliquoted, desalted, concentrated on a C18-ZipTip (Millipore), and eluted with 50% acetonitrile in 0.1% formic acid, then dried in SpeedVac. The heavy isotope-labeled peptides (25 pg each) were spiked into the digested samples (0.5 ug) prior to the mass spectrometry analysis.

Liquid Chromatography - Mass Spectrometry Analysis

NanoLC−nanoESi-parallel reaction monitoring (PRM) analysis (37,38) was performed on a Thermo UltiMate 3000 RSLCnano system connected to an Thermo Orbitrap Fusion mass spectrometer (Thermo Fisher Scientific) equipped with a nanospray interface (New Objective) (37,38). Peptide mixtures were loaded onto a 75 μm ID, 25 cm length C_{18} BEH column (Waters, Milford, MA) packed with 1.7 μm particles with a pore width of 130 Å and were separated using a segmented gradient in 30 min from 5% to 35% solvent B (0.1% formic acid in acetonitrile) at a flow rate of 300 nl/min. Solvent A was 0.1% formic acid in water. The mass spectrometer was operated in the PRM mode. Briefly, the MS scans of peptide precursors from 350 to 1600 m/z were performed at 120K resolution with a 2×10^5 ion count target. The isolation list contains 2 target peptides from calmodulin with their heavy isotope-labeled versions (Supplemental Figure S2A and Supplemental Table S5). The PRM scans were performed by isolation window at 1.6 Da with the quadrupole, HCD fragmentation with normalized collision energy of 30, and analysis at 30K resolution in the orbitrap. The PRM scans ion count target was set to 1×10^6 and the max injection time was 54 ms. The PRM scans were triggered by time-scheduled targeting precursor ions that were selected for target peptides in ±2 min elution windows. The PRM data was processed using Skyline 22.2.0.255 (MacCoss Lab Software) to generate extracted ion chromatograms and perform peak integration. All peaks were manually inspected to confirm correct detection and peak boundaries. Peak integration and calculation of the ratios between light endogenous and the heavy-labeled peptide (L/H) were done in Skyline and result reports exported from the software. Light to heavy peptide peak area ratios were used for the target peptides quantitation analysis.

Epicardial optical voltage mapping of Langendorff-perfused hearts

High-resolution optical mapping experiments were performed on 4- to 6-month-old mice as described previously (7). In brief, hearts were isolated and retrogradely perfused as Langendorff preparations with warm (37°C) Krebs-Henseleit solution (pH 7.4 when gassed with a mixture of 95% O_2 and 5% CO_2) at an aortic pressure of 70 cm H_2O. After 10 minutes of stabilization, the hearts were stained with the voltage-sensitive dye RH237 (1.25mg/ml in DMSO; Invitrogen) in the presence of blebbistatin (10 µmol/L in DMSO; Tocris Bioscience). The stained hearts were excited with a laser at a 532-nm wavelength and the fluorescence was collected by a MiCAM Ultima cameras (MiCAM 05, BrainVision) through a 715-nm long-pass filter. A volume-conducted ECG was continuously recorded.

To assess repolarization, RH237-stained hearts were mapped in the presence of the excitation-contraction uncoupler blebbistatin (10 µmol/L). Optical maps were obtained from the anterior epicardial surface at baseline and in the presence of isoproterenol (100 nM dissolved in ddH_2O). Optical signals were processed with both signal (3x3 pixels Gaussian filter) and temporal (3 frames moving average) filtering. Then signals were averaged across the entire map and these spatially averaged signals were used to measure APD at 30%, 50% and 80% repolarization (APD_{30}, APD_{50} and APD_{80}). Maps of isochrone repolarization times were generated. The center of the left ventricular lateral wall was paced with a platinum unipolar electrode at a cycle length of 120 ms and the epicardial activation pattern was recorded. Conduction velocities were measured, as described previously (7).

Isolation of single ventricular myocytes

Mouse left ventricular myocytes were isolated enzymatically by a well-established protocol (36). Adult mice were anesthetized with i.p. urethane injection, followed by thoracotomy to expose the heart and cut down the inferior vena cava and descending aorta. The

heart was flushed with 7 ml EDTA buffer (5 mM) injected into the right ventricular chamber within 1-2 minutes. The ascending aorta was clamped and the heart was cut and immersed into fresh EDTA buffer in a 3.5 cm Petri dish. The left ventricular chamber was injected with 10 mL EDTA buffer, 3 mL EDTA-free perfusion buffer, and 30 to 50 mL digestion buffer sequentially. Next, the right ventricular free wall and the atria were removed, left ventricle was transferred to a dish filled with perfusion buffer containing 5% sterile FBS (stop buffer). The tissue was minced into small pieces, triturated, and filtered through a 100-µm Nylon mesh. Cell suspension underwent sequential rounds of gravity settling, using 3 intermediate calcium reintroduction buffers (0.1, 0.2 and 0.5 mM $CaCl_2$ Tyrode's solution) to gradually bring up calcium concentration. The resuspended cells were stored in 0.5 mmol/L $CaCl_2$ Tyrode's solution and used within 6-8 h. Only calcium tolerant, quiescent, and rod-shaped cells were studied.

Cellular electrophysiology

Voltage-clamp recordings were performed at room temperature using the patch-clamp technique in whole-cell ruptured-patch configuration. Microelectrodes were pulled from borosilicate glass capillaries (Sutter Instruments) using a Sutter P-97 puller. Currents were acquired using an Axoptach 200B and Clampex10 software (Molecular Devices). Whole-cell currents were low pass filtered at 1 kHz, and then digitally sampled at 5 kHz. P/4 leak subtraction protocol was used. The pipette potential was zeroed before seal formation, and voltages were not corrected for liquid junction potential. Capacitance transients were cancelled using the computer-controlled circuitry of the amplifier, and voltage errors were minimized using 60-70% series resistance compensation. Series resistance before compensation was typically 5-9 MΩ. After establishing the whole-cell configuration, the myocytes were allowed to equilibrate for 5 min with the internal solute before the data were collected. Bath solution for

$I_{Ca.L}$ recording contained (in mM) 140 TEA-MeSO$_3$, 10 HEPES (pH 7.4), and 5 $CaCl_2$ or 5 $BaCl_2$. Osmolarity was adjusted to 300 mOsm, using TEA-MeSO$_3$. Pipette solution for $I_{Ca.L}$ recording contained (in mM): 114 CsMeSO$_3$; 5 CsCl, 1 $MgCl_2$, 4 MgATP, 10 HEPES (pH 7.3), 10 BAPTA, and 0.005 ryanodine, at 295 mOsm adjusted with CsMeSO$_3$. To evoke $I_{Ca.L}$, cells were first depolarized for 1 s to -50 mV from a holding potential of -70 mV, followed by 400-ms pulses to +10 mV and repolarization to the holding potential. The interval between voltage steps was 5 s. The fraction of current remaining 50 ms after the peak (r_{50}) was measured. The extent of Ca^{2+}/CaM-dependent inactivation (CDI) was calculated as $f_{50} = (r_{50/Ba} - r_{50/Ca})/r_{50/Ba}$ (6).

Supplemental Figure S1

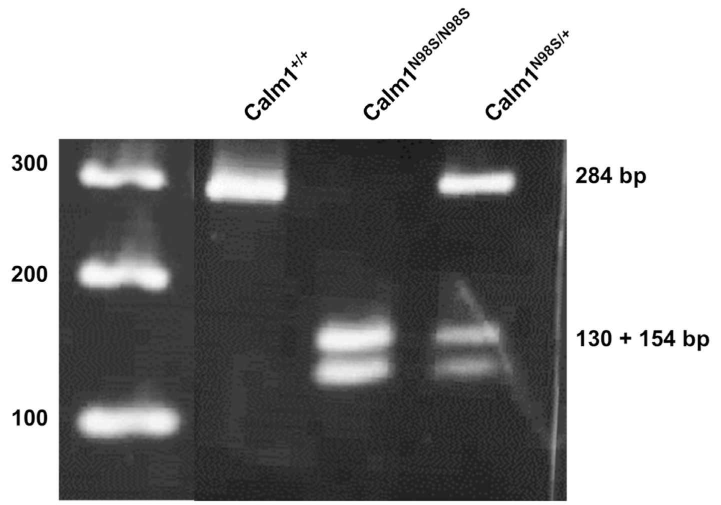

Supplemental Figure S1. Genotype analysis of offspring derived from *Calm1*$^{N98S/+}$ intercrosses by Restriction Fragment Length Polymorphism (RFLP). A 284-bp DNA segment spanning exon 5 of *Calm1* was amplified using PCR. The resulting PCR amplicons were digested with *BsrB1* and fractionated on a 2.3 % agarose gel by electrophoresis. Shown are PCR-RFLP products for wildtype (*Calm1*$^{+/+}$), heterozygous mutant (*Calm1*$^{N98S/+}$), and homozygous mutant (*Calm1*$^{N98S/N98S}$) mice.

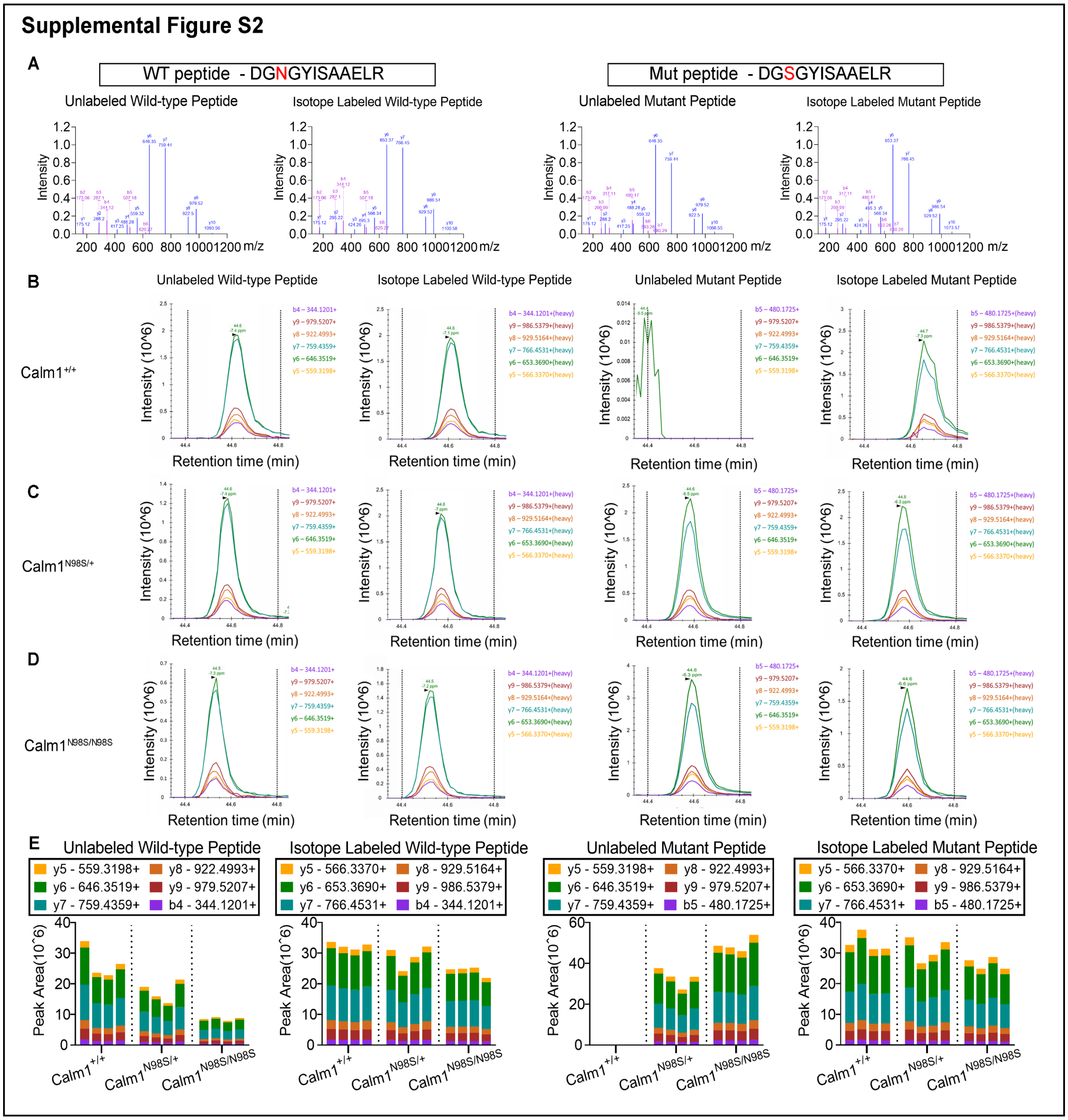

Supplemental Figure S2. Quantification of wildtype and N98S-CALM abundances using parallel reaction monitoring. (**A**) MS/MS spectra of the unlabeled (left panels) and the heavy-isotope labeled (right panels) target peptides for wildtype and N98S-CALM. Boxes show amino acid compositions of the N98- and S98-harboring peptides that are obtained by enzymatic cleavage of wildtype or mutated CALM, respectively. (**B - D**) MS/MS spectra shown in A were used for identification and extraction of fragment ion chromatograms that were obtained from left ventricular digests spiked with unlabeled and heavy isotope-labeled target peptides for wildtype and N98S CALM. Subset of fragment ions with highest intensities in the MS/MS spectrum were used for quantification via integration. Peak areas of fragment ions were extracted using <10 ppm mass window and integrated across the elution profile. Chromatograms display peak area contributions of the individual fragment ions of the unlabeled and heavy isotope-

labeled peptides. Mass measurement errors and retention times of the most intense transitions are annotated above the peaks. The vertical dashed lines on either side of the peaks indicate the integration boundaries for the peaks. (**E**) Total integrated fragment ion signals for the unlabeled and heavy isotope-labeled peptides in left ventricular digests of *Calm1*$^{+/+}$*, Calm1*$^{N98S/+}$, and *Calm1*$^{N98S/N98S}$ male mice (4 per genotype) are plotted as bar graphs; contribution from each individual fragment ion is displayed as a different color in the bars.

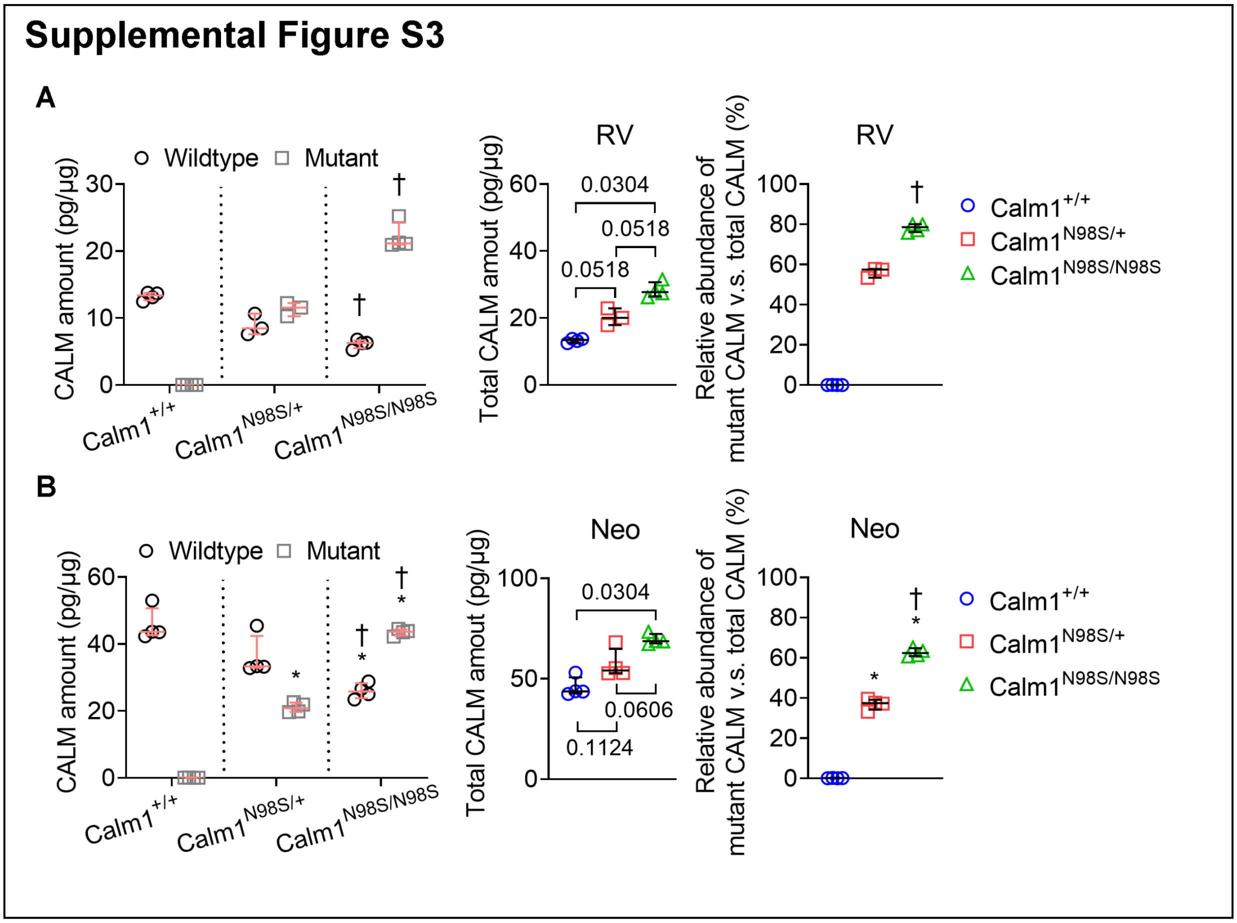

Supplemental Figure S3. Assessment of total and mutant CALM protein expression in adult right ventricle and in neonatal hearts using LC-MS/MS. (**A** and **B**) Wildtype and mutant CALM protein levels (normalized to the total protein amount per sample) (left panels), total CALM protein levels (middle panels), and mutant CALM protein levels expressed as percentage of total CALM protein levels (right panels) in adult right ventricle (A; RV) and in neonatal hearts (B; Neo) from *Calm1*$^{+/+}$, *Calm1*$^{N98S/+}$ and *Calm1*$^{N98S/N98S}$ male littermates. Data are displayed as scatter dot plots with horizontal lines denoting the median and interquartile ranges from 4 mice each per genotype. The Kruskal-Wallis test and the Wilcoxon-Rank-Sum test were used to compare CALM protein levels among genotypes. † $P = 0.030$ vs *Calm1*$^{+/+}$ in A; * $P = 0.03$ vs *Calm1*$^{+/+}$ and † $P = 0.03$ versus *Calm1*$^{N98S/+}$ in B. Middle panels: Numbers above brackets indicate P values for pairwise comparisons using the Wilcoxon Rank Sum test.

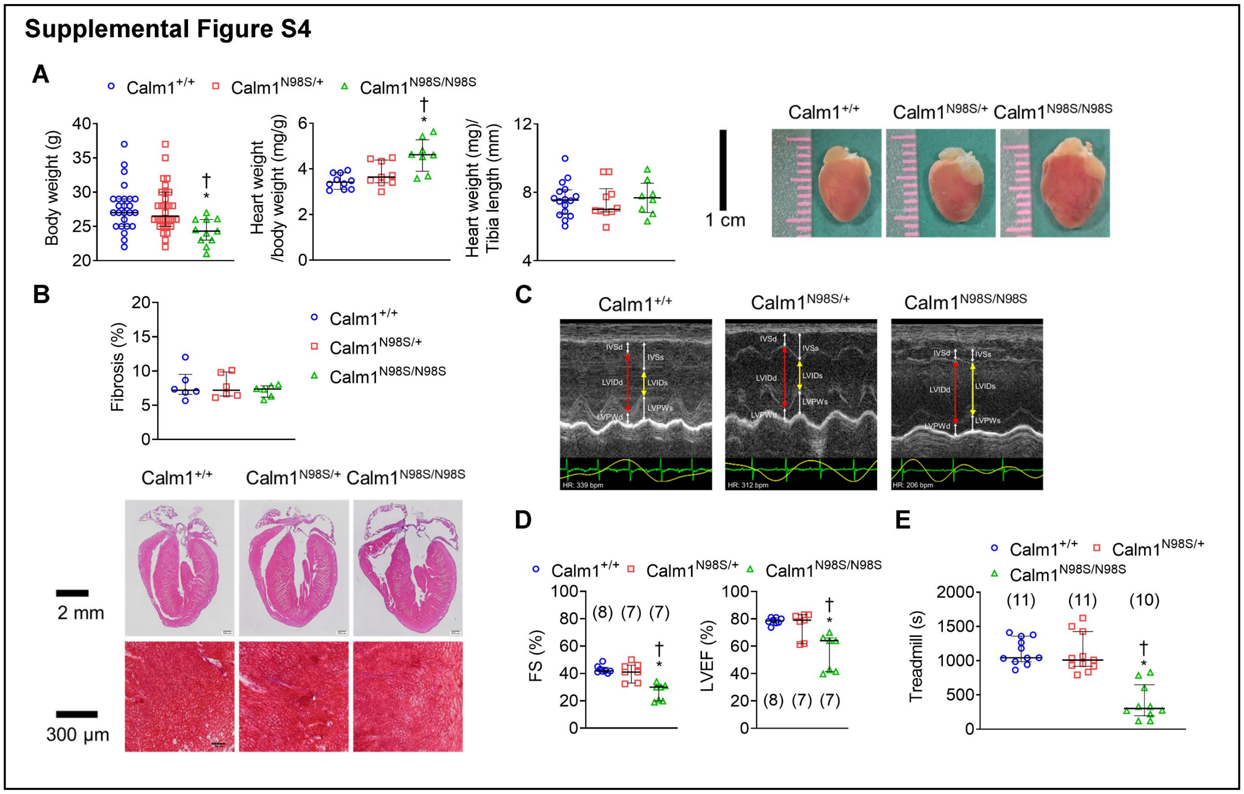

Supplemental Figure S4. **Genotype-phenotype correlations**. (**A**) Scatter dot plots of bodyweight, heart weight to body weight ratio, and heart weight to tibial length ratio. Horizontal lines denote mean ± SEM. Data are from 25 *Calm1*$^{+/+}$, 30 *Calm1*$^{N98S/+}$ and 11 *Calm1*$^{N98S/N98S}$ male mice. * $P \leq 0.004$ compared to *Calm1*$^{+/+}$; † $P \leq 0.006$ compared to *Calm1*$^{N98S/+}$ by a linear model with independent term for genotype for pairwise comparisons. Right panel shows representative images of whole hearts. (**B**) Upper panel: dot plots of percentage of cardiac fibrosis. Horizontal lines denote medians and interquartile ranges. Data are from 6 hearts per genotype. There were no significant differences among the genotypes ($P > 0.05$ by ANOVA); middle and lower panels: representative images of hematoxylin and eosin (H&E)-stained 4-chamber sections and Masson's trichrome-stained microscopic sections from left ventricles of adult mice. (**C**) Exemplary M-mode echocardiograms (short-axis views) recorded from the left ventricles of adult mice. Red and yellow arrows mark ventricular internal dimensions at end diastole and end systole, respectively. (**D** and **E**) Scatter dot plots of fractional shortening (FS), left ventricular ejection fraction (EF; panel D) and treadmill time to exhaustion (panel E) in adult mice. Numbers in parentheses demark number of animals. Horizontal lines depict median and interquartile range. *$P \leq 0.0001$ compared to *Calm1*$^{+/+}$; † $P \leq 0.0008$ compared to *Calm1*$^{N98S/+}$ by a linear model with independent term for genotype.

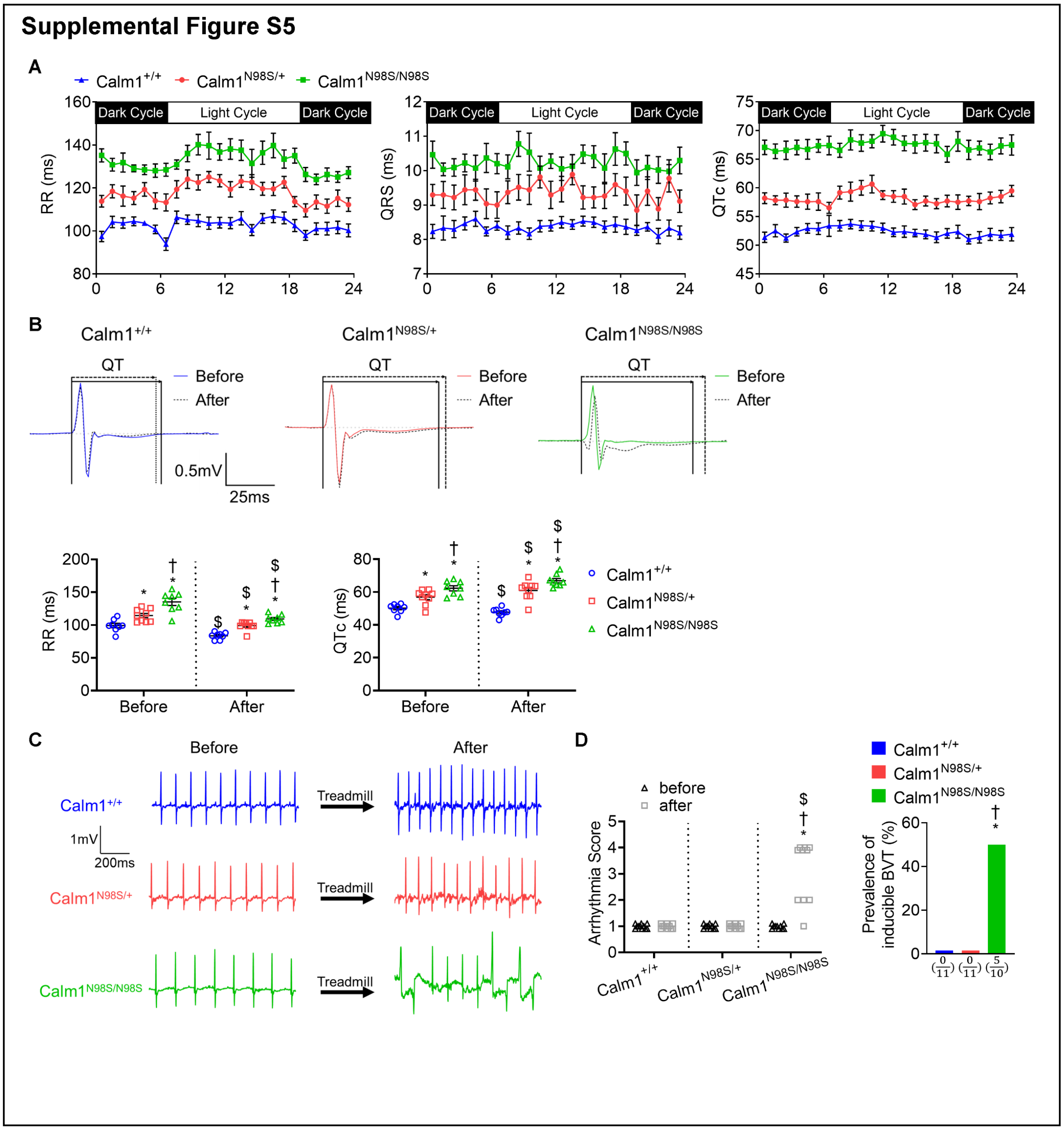

Supplemental Figure S5. Electrocardiographic phenotype-genotype correlations. (**A**) Diurnal variations in RR, QRS and QT_c intervals in ambulatory male littermates. Data are presented as mean ± SEM from 10 *Calm1*$^{+/+}$, 9 *Calm1*$^{N98S/+}$ and 9 *Calm1*$^{N98S/N98S}$ mice. (**B**) ECG changes in response to cage switch. Upper panels show superimpositions of ensemble averaged ECGs recorded before and after cage switch. Lower panels: Scatter dot plots of changes in RR (left) and QT_c intervals in response to a cage switch. All data were normally distributed. Horizontal lines superimposed on dots denote mean ± SEM from 8 *Calm1*$^{+/+}$, 9 *Calm1*$^{N98S/+}$ and 8 *Calm1*$^{N98S/N98S}$ mice. * $P \leq 0.015$ vs. *Calm1*$^{+/+}$; † $P \leq 0.011$vs *Calm1*$^{N98S/+}$; $ $P \leq 0.0123$ vs before cage switch of the same genotype. Data were modeled by a repeated measures linear model with independent terms for genotype, time period, and the interaction of the two. An unstructured covariance matrix was used. (**C**) Examples of treadmill exercise-provoked

electrocardiographic responses. (**D**) Ventricular arrhythmia scores (left panel) and percentage of mice developing BVT during treadmill exercise. * $P \leq 0.0124$ vs *Calm1*$^{+/+}$; † $P \leq 0.0124$ vs *Calm1*$^{N98S/+}$; $ $P = 0.0039$ vs before treadmill. *P*-values from comparison of measures after treadmill between genotypes by Fisher's exact test. *P*-values for comparison of change in measures after versus before within genotype by the Sign test.

Supplemental Figure S6

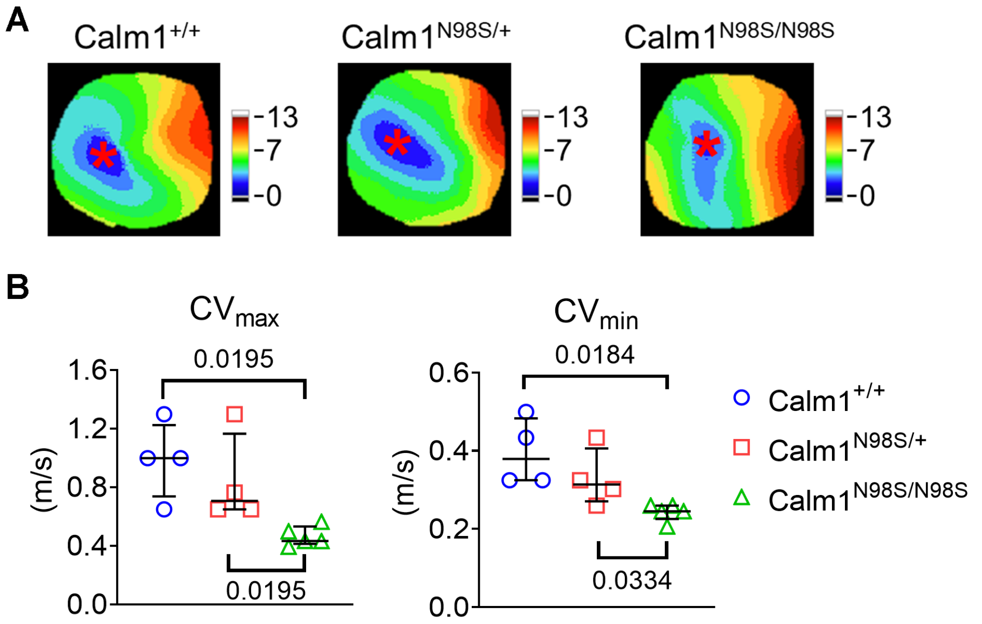

Supplemental Figure S6. **Epicardial ventricular conduction velocities.** (**A**) Ventricular activation isochrone maps of the anterior aspect of a *Calm1*$^{+/+}$, *Calm1*$^{N98S/+}$ and *Calm1*$^{N98S/N98S}$ heart. Asterisks denote positions of the pacing electrodes. The ventricles were paced at a cycle length of 120 ms. The anisotropic propagation pattern enables measurements of CV_{max} and CV_{min}. Note smaller isochrone width in the homozygous mutant heart suggesting conduction slowing. (**B**) Dot plots of conduction velocities. Horizontal lines denote medians and interquartile ranges. *Calm1*$^{+/+}$: N = 4; *Calm1*$^{N98S/+}$: N = 4; *Calm1*$^{N98S/N98S}$: N = 5. Numbers next to brackets denote *P* values by Kruskal Wallis test followed by the Wilcoxon Rank Sum test for pairwise comparisons.

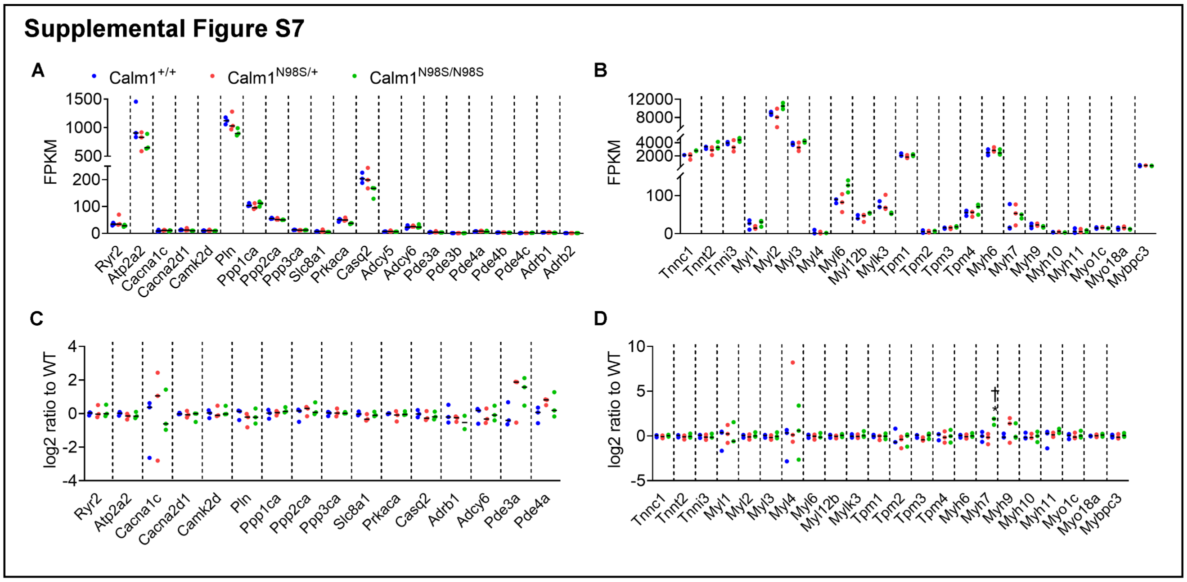

Supplemental Figure S7. **Cardiac transcriptomic and proteomic profiling using RNA-sequencing and LC-MS/MS.** (**A** and **B**) Cardiac (left ventricle) RNA-sequencing analyses of genes encoding proteins that are important for excitation-contraction coupling and adrenergic signaling. Data are shown as dot plots. Horizontal lines denote medians. There were no significant differences among the genotypes ($P > 0.05$ by Wilcoxon rank sum test; N = 3 per genotype). FPKM, Fragments Per Kilobase of transcript per Million mapped reads. For gene abbreviations see Supplemental Table S6. (**C** and **D**) Dot plots of relative expressions (log 2) of proteins mediating excitation-contraction coupling, contraction, and adrenergic signaling in mutant left ventricle relative to Calm1$^{+/+}$. There were no significant differences among the genotypes ($P > 0.05$ by empirical Bayes moderated t-test, while adjusted P was based on Benjamini-Hochberg method to adjust P values for multiple hypothesis testing (38); N = 3 per genotype). Abbreviations are the same as in (A) and (B).

Supplemental Figure S8

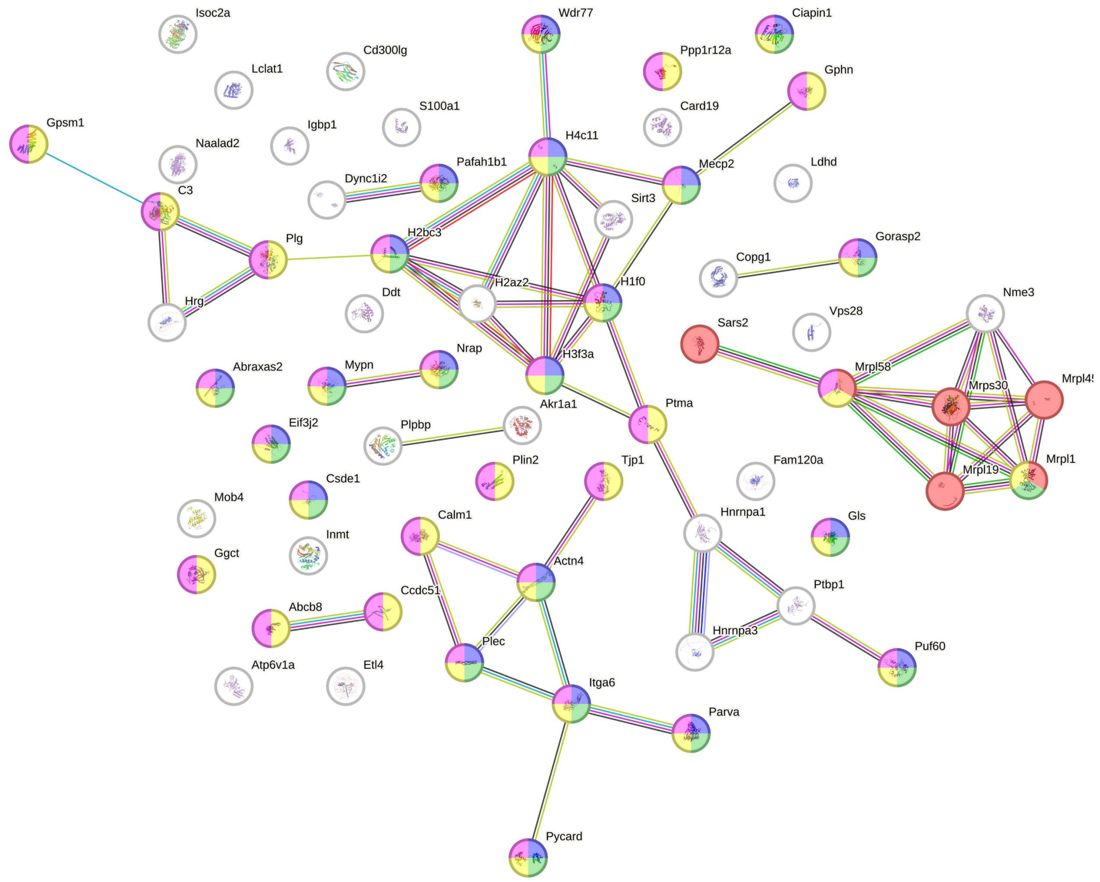

Color	Pathway name	Gene count in network	strength	False discovery rate
● (red)	Mitochondrial translation	6 of 109	1.27	0.0187
● (blue)	Cellular component assembly	21 of 2396	0.47	0.0187
● (yellow)	Cellular component biogenesis	22 of 2631	0.45	0.0187
● (green)	Cellular component organization or biogenesis	35 of 5681	0.32	0.0187
● (pink)	Cellular component organization	34 of 5482	0.32	0.0187

Supplemental Figure S8. **Functional enrichment analysis of biological processes using STRING and Gene Ontology database between *Calm1*$^{+/+}$ and *Calm1*$^{N98S/+}$ left ventricular samples.** The top 5 pathways of the significantly differentially expressed proteins from Calm1$^{+/+}$ vs. Calm1$^{N98S/+}$ LV samples were associated with mitochondria translation, cellular component assembly, biogenesis, and/or organization with 57 interactions among 65 proteins. The nodes represent proteins in the protein-protein interaction network, and the edges represent the interaction between proteins.

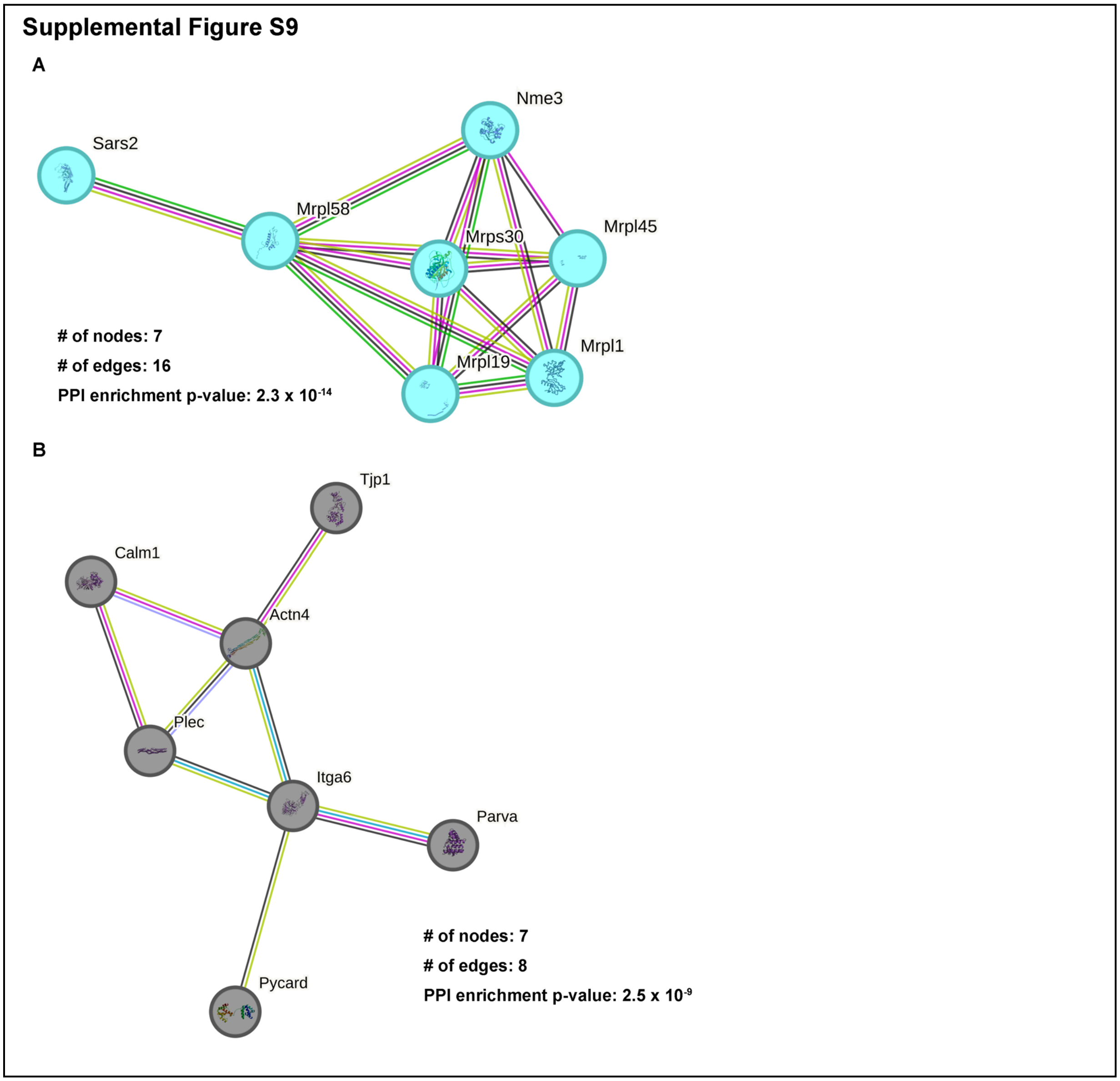

Supplemental Figure S9. **Protein–protein network analysis with k-mean clustering using the STRING database between *Calm1*$^{+/+}$ and *Calm1*$^{N98S/+}$ left ventricular samples.** Among the major networks identified in these significantly differentially expressed proteins from *Calm1*$^{+/+}$ vs. *Calm1*$^{N98S/+}$ LV samples, k-means clustering highlighted mitochondrial translation (A) and Type I hemidesmosome assembly (B) —essential for energy production and tissue integrity. PPI: protein-protein interaction.

Supplemental Figure S10

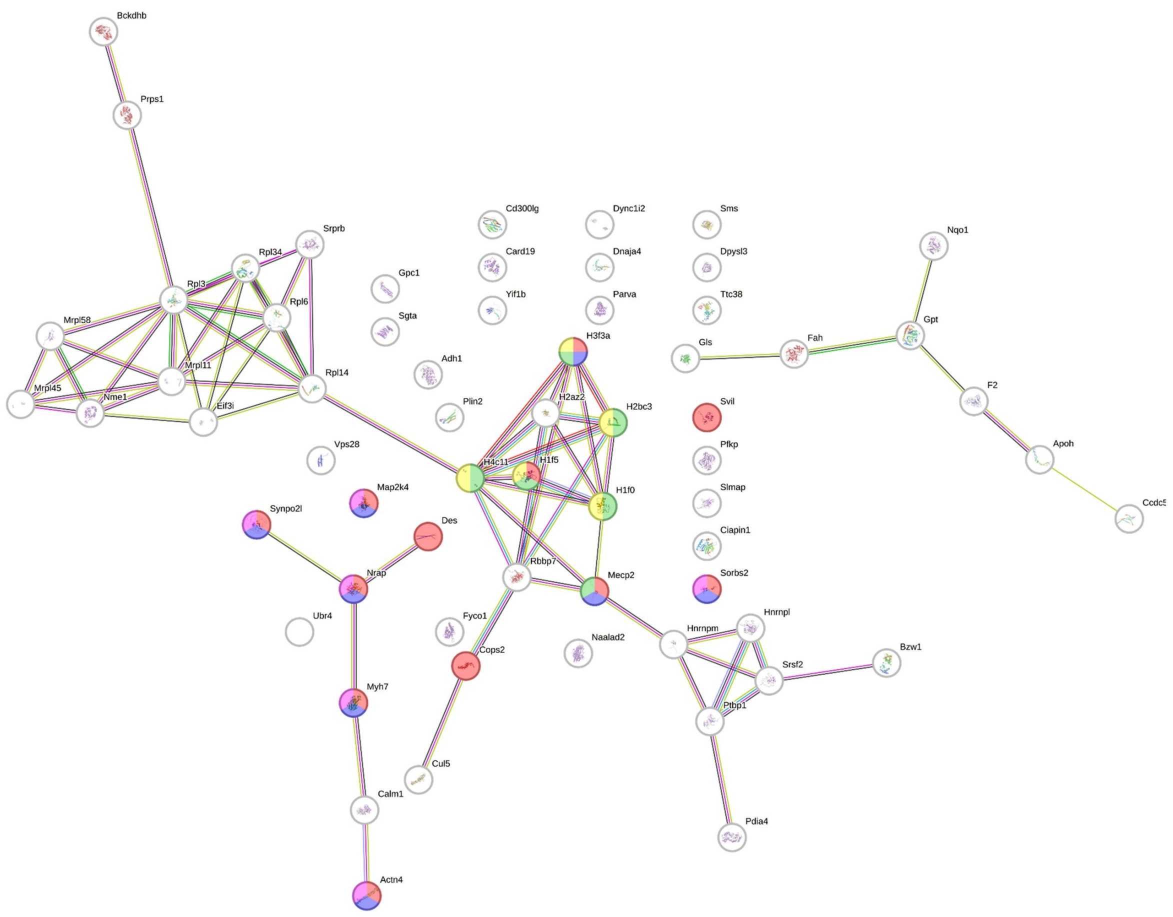

Color	Pathway name	Gene count in network	Strength	False discovery rate
(red)	Muscle structure development	12 of 535	0.88	0.00083
(blue)	Muscle cell differentiation	8 of 314	0.94	0.0268
(yellow)	Chromatin assembly	6 of 163	1.1	0.0396
(green)	Nucleosome assembly	5 of 102	1.22	0.0487
(pink)	Muscle cell development	6 of 182	1.05	0.0487

Supplemental Figure S10. S**TRING-based functional enrichment analysis of biological processes using the Gene Ontology database in *Calm1*$^{+/+}$ and *Calm1*$^{N98S/N98S}$ left ventricular samples.** The top 5 pathways enriched significantly differentially expressed proteins from *Calm1*$^{+/+}$ vs. *Calm1*$^{N98S/N98S}$ left ventricular samples were linked to muscle structure development, muscle cell differentiation, chromatin and nucleosome assembly, and muscle cell development, involving 72 interactions among 65 proteins. In the protein-protein interaction network, nodes represent proteins, while edges indicate interactions between them.

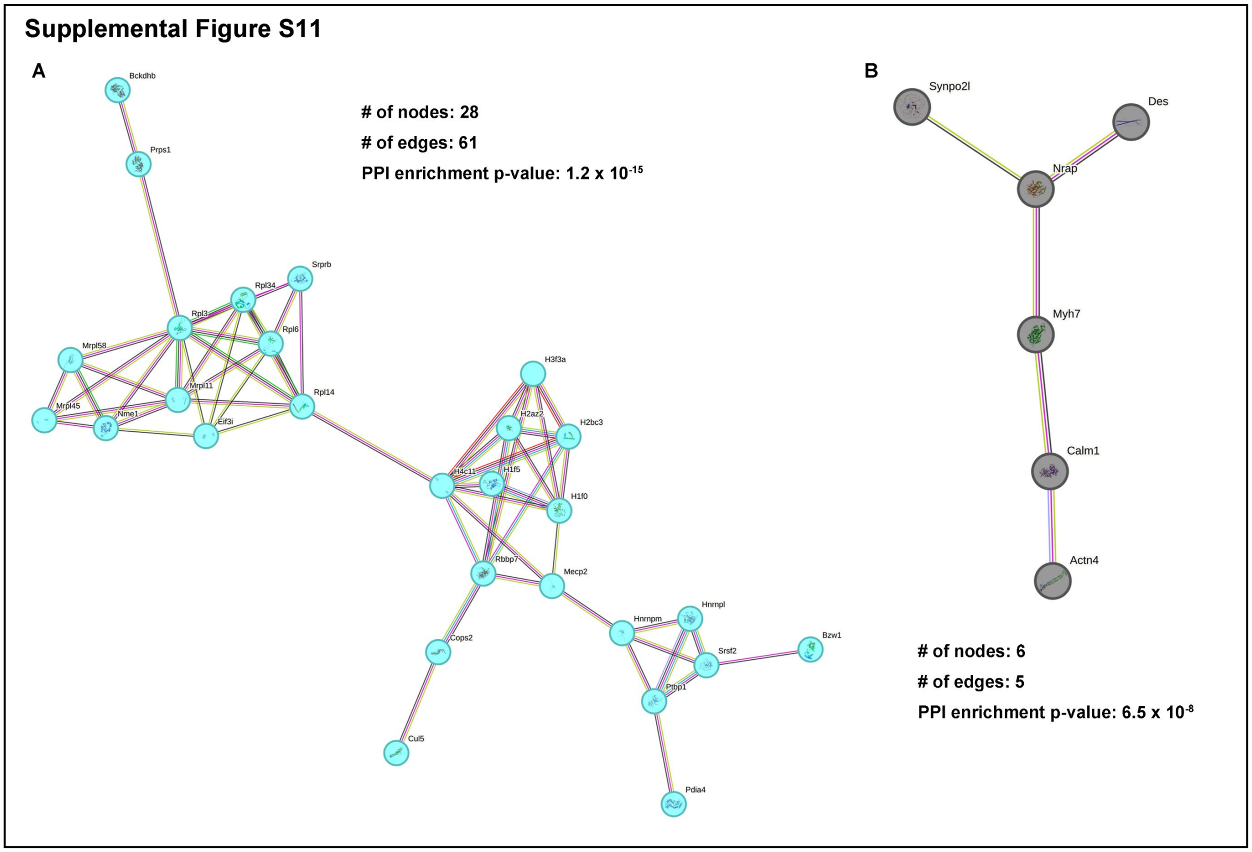

Supplemental Figure S11. STRING-based protein-protein interaction analysis with k-means clustering in *Calm1*$^{+/+}$ and *Calm1*$^{N98S/N98S}$ left ventricular samples. In the significantly differentially expressed proteins from *Calm1*$^{+/+}$ vs. *Calm1*$^{N98S/N98S}$ left ventricular samples, k-means clustering identified two major network clusters: the large ribosome subunit (A) and fascia adherens (B) —critical for protein synthesis and cardiac contraction. PPI: protein-protein interaction.

Supplemental Table S1.

Genotype frequencies among E18 embryos derived from *Calm1*$^{N98S/+}$ intercrosses.

	**Number of E18 Embryos**	**Percent**
**Calm1$^{+/+}$**	24	25
**Calm1$^{N98S/+}$**	51	54
**Calm1$^{N98S/N98S}$**	20	21
**Total**	95	100

E18 embryos were harvested from 11 *Calm1*$^{N98S/+}$ intercrosses. χ^2 analysis was used for genotypic ratios. Observed proportions are consistent with expected Mendelian inheritance (P = 0.65).

Supplemental Table S2.

RT-PCR - based estimates of absolute *Calm* gene transcript levels in atria and right ventricle

Calm1

**Tissue**	**Genotype**	***Calm1*** ΔC_T **(Avg. *Calm1*** C_T **– Avg. *Gapdh*** C_T**)**	***Calm1*** $\Delta\Delta C_T$ **(Avg.** ΔCT **– Avg.** $\Delta C_{T,\ Calm1,\ LV}$**)**	**Normalized *Calm1* amount relative to LV** $2^{-\Delta\Delta C_T}$	**Measured LV FPKM**	**Estimated FPKM (LV FPKM x** $2^{-\Delta\Delta C_T}$**)**
LV	$Calm1^{+/+}$	6.75 ± 0.38	0.00 ± 0.38	1.0 (0.77 – 1.30)	37.75	
LV	$Calm1^{N98S/+}$	7.02 ± 0.39	0.00 ± 0.39	1.0 (0.76 – 1.31)	34.76	
LV	$Calm1^{N98S/N98S}$	7.69 ± 0.25	0.00 ± 0.25	1.0 (0.84 – 1.19)	34.21	
RV	$Calm1^{+/+}$	6.90 ± 0.32	0.16 ± 0.32	0.90 (0.72 – 1.12)	37.75	33.88 (25.60 – 40.53)
RV	$Calm1^{N98S/+}$	7.44 ± 0.29	0.42 ± 0.29	0.75 (0.61 – 0.91)	34.76	25.98 (20.14 – 30.75)
RV	$Calm1^{N98S/N98S}$	7.99 ± 0.34	0.3 ± 0.34	0.81 (0.64 – 1.03)	34.21	27.79 (21.87 – 33.57)
Atria	$Calm1^{+/+}$	6.42 ± 0.26	-0.33 ± 0.26	1.26 (1.05 – 1.51)	37.75	47.45 (38.11 – 55.09)
Atria	$Calm1^{N98S/+}$	6.7 ± 0.51	-0.32 ± 0.51	1.25 (0.88 – 1.78)	34.76	43.40 (24.84 – 56.32)
Atria	$Calm1^{N98S/N98S}$	6.81 ± 0.26	-0.88 ± 0.26	1.84 (1.54 – 2.20)	34.21	62.96 (50.47 – 73.30)

Calm2

**Tissue**	**Genotype**	***Calm2*** ΔC_T **(Avg. *Calm2*** C_T **– Avg. *Gapdh*** C_T**)**	***Calm2*** $\Delta\Delta C_T$ **(Avg.** ΔCT **– Avg.** $\Delta C_{T,\ Calm2,\ LV}$**)**	**Normalized *Calm2* amount relative to LV** $2^{-\Delta\Delta C_T}$	**Measured LV FPKM**	**Estimated FPKM (LV FPKM x** $2^{-\Delta\Delta C_T}$**)**

LV	Calm1$^{+/+}$	11.03 ± 0.40	0.00 ± 0.40	1.0 (0.76 – 1.32)	58.04	
LV	Calm1$^{N98S/+}$	11.28 ± 0.19	0.00 ± 0.19	1.0 (0.87 – 1.14)	49.04	
LV	Calm1$^{N98S/N98S}$	11.58 ± 0.31	0.00 ± 0.31	1.0 (0.81 – 1.24)	53.23	
RV	Calm1$^{+/+}$	10.83 ± 00.29	-0.20 ± 0.29	1.15 (0.94 – 1.41)	58.04	66.88 (52.04 – 79.03)
RV	Calm1$^{N98S/+}$	11.15 ± 0.21	-0.13 ± 0.67	1.09 (0.69 – 1.74)	49.04	53.63 (45.06 – 61.02)
RV	Calm1$^{N98S/N98S}$	11.11 ± 0.41	-0.47 ± 0.41	1.39 (1.04 – 1.85)	53.23	73.82 (55.43 – 92.21)
Atria	Calm1$^{+/+}$	9.38 ± 0.17	-1.65 ± 0.17	3.13 (2.78 – 3.53)	58.04	181.92 (158.84 – 202.41)
Atria	Calm1$^{N98S/+}$	9.63 ± 0.64	-1.65 ± 0.64	3.14 (2.01 – 4.90)	49.04	153.85 (67.49 – 209.16)
Atria	Calm1$^{N98S/N98S}$	9.83 ± 0.29	-1.76 ± 0.29	3.38 (2.76 – 4.13)	53.23	179.82 (139.67 – 212.64)

Calm3

**Tissue**	**Genotype**	***Calm3* ΔC_T (Avg. *Calm3* C_T – Avg. *Gapdh* C_T)**	***Calm3* $\Delta\Delta C_T$ (Avg. ΔCT – Avg. $\Delta C_{T, Calm3, LV}$)**	**Normalized *Calm3* amount relative to LV $2^{-\Delta\Delta C_T}$**	**Measured LV FPKM**	**Estimated FPKM (LV FPKM x $2^{-\Delta\Delta C_T}$)**
LV	Calm1$^{+/+}$	10.84 ± 0.50	0.00 ± 0.50	1.0 (0.71–1.41)	44.21	
LV	Calm1$^{N98S/+}$	11.14 ± 0.38	0.00 ± 0.38	1.0 (0.77 – 1.30)	41.39	
LV	Calm1$^{N98S/N98S}$	11.68 ± 0.37	0.00 ± 0.37	1.0 (0.77 – 1.30)	35.74	
RV	Calm1$^{+/+}$	11.12 ± 0.31	0.28 ± 0.31	0.82 (0.66 – 1.02)	44.21	36.47 (27.66 – 43.55)

RV	Calm1$^{N98S/+}$	11.38 ± 0.37	0.25 ± 0.37	0.84 (0.65 – 1.09)	41.39	34.86 (24.53 – 42.83)
RV	Calm1$^{N98S/N98S}$	11.58 ± 0.38	-0.09 ± 0.38	1.07 (0.82 – 1.39)	35.74	38.15 (29.38 – 46.91)
Atria	Calm1$^{+/+}$	11.07 ± 0.23	0.24 ± 0.23	0.85 (0.73 – 0.99)	44.21	37.54 (31.16– 42.99)
Atria	Calm1$^{N98S/+}$	10.83 ± 0.41	-0.31 ± 0.14	1.24 (0.93 – 1.65)	41.39	51.23 (34.38 – 64.05)
Atria	Calm1$^{N98S/N98S}$	11.01 ± 0.28	-0.67 ± 0.28	1.59 (1.31 – 1.93)	35.74	56.84 (44.77– 66.80)

Values are mean [for Measured LV FPKM], mean ± SEM [for ΔC_T and $\Delta\Delta C_T$] and mean ($2^{-\Delta\Delta C_T+SEM}$ – $2^{-\Delta\Delta C_T-SEM}$) [for Normalized *Calm* relative to LV $2^{-\Delta\Delta C_T}$ and Estimated FPKM] from 8 *Calm1*$^{+/+}$, 7 *Calm1*$^{N98S/+}$ and 7 *Calm1*$^{N98S/N98S}$ animals. Mean fold-changes in expression of each *Calm* gene in the atria and right ventricle (RV) relative to their expression in left ventricle (LV) were calculated by $2^{-\Delta\Delta C_T}$, where $\Delta\Delta C_T$ (Atria) = average($\Delta C_{T,atria,Calm}$) - average($\Delta C_{T,LV,Calm}$) and $\Delta\Delta C_T$ (RV) = average($\Delta C_{T,RV,Calm}$) - average($\Delta C_{T,LV,Calm}$). The fold-change in *Calm* expression in the atria or RV relative to LV was then multiplied by the mean LV FPKM value for the corresponding *Calm* gene and genotype (LV FPKM x $2^{-\Delta\Delta C_T}$) to obtain estimates of absolute *Calm* gene transcript levels.

Supplemental Table S3.

Echocardiographic data in *Calm1*$^{+/+}$, *Calm1*$^{N98S/+}$ and *Calm1*$^{N98S/N98S}$ male littermate mice

Parameters	Calm1$^{+/+}$ (N=8)	Calm1$^{N98S/+}$ (N=7)	Calm1$^{N98S/N98S}$ (N=7)
IVSd (mm)	0.9±0.06	0.9±0.05	0.9±0.04
IVSs (mm)	1.5±0.06	1.4±0.05	1.2±0.03 $^{*,\#}$
LVIDd (mm)	3.5±0.09	3.6±0.18	3.7±0.09
LVIDs (mm)	2.0±0.07	2.1±0.17	2.7±0.15 $^{*,\#}$
PWd (mm)	0.9±0.06	0.9±0.05	0.9±0.06
PWs (mm)	1.4±0.05	1.3±0.07	1.0±0.06 $^{*,\#}$
EDV (μL)	52.2±3.33	57.0±6.28	56.8±3.27
ESV (μL)	13.3±1.26	16.2±3.25	27.9±3.7 $^{*,\#}$
EF (%)	78.4±0.87	75.4±3.66	55.4±5.06 $^{*,\#}$
FS (%)	42.9±1	41.2±2.49	26.4±2.36 $^{*,\#}$

Interventricular septal thickness (IVS), left ventricular internal dimension (LVID) and posterior wall thickness (PW) at diastole and systole (IVSd, IVSs, LVIDs, LVIDd, LVPWd and LVPWs, respectively); EDV, end-diastolic volume; ESV, end-systolic volume; EF, ejection fraction; FS, fractional shortening. For comparisons of list parameters, a linear model with independent term for genotype was used. Values are mean ± SEM. * $P \leq 0.0099$ compared to *Calm1*$^{N98S/+}$; # $P \leq 0.0014$ compared to *Calm1*$^{+/+}$; N, number of mice.

Supplemental Table S4.

Primers for Quantitative Real Time PCR

Gene	Forward Primer	Reverse Primer
Calm1	5′- TGTCCGTGGTGCCGTTACTC -3′	5′- CTGATCAGCCATGGTGCGAG -3′
Calm2	5′- GTGGAGCGAGCGAGTCGAG -3′	5′- TTTGAACTCTGCAATCTGCTCTTC -3′
Calm3	5′-AGTAACCTCGATCCCCGAGC -3′	5′- TGGTGCCATCTCCATCCTTG -3′
Gapdh	5′-GCCAAGGTCATCCATGACAAC -3′	5′-AGTGTAGCCCAAGATGCCCTT -3′

Supplemental Table S5.

Properties of non-labeled and heavy isotope-labeled target peptides for LC/MS-MS.

**Abbreviation**	**Peptide sequence**	**Precursor m/z**	**Charge state**	**Product ions used for PRM**	**RT (min)**
WT	DGNGYISAAELR	633.3097	2+	b4+, y5+, y6+, y7+, y8+, y9+	43.43
WT-heavy	DGNGYISAAELR	636.8182	2+	b4+, y5+, y6+, y7+, y8+, y9+	43.41
Mut	DGSGYISAAELR	619.8042	2+	b5+, y5+, y6+, y7+, y8+, y9+	43.38
Mut-heavy	DGSGYISAAELR	623.3128	2+	b5+, y5+, y6+, y7+, y8+, y9+	43.36

WT, wildtype; Mut, mutant; RT, retention time; PRM, parallel reaction monitoring

Supplemental Table S6.

List of gene abbreviations for Figure 8

RyR2	cardiac ryanodine receptor isoform 2
Atp2a2	sarcoplasmic-endoplasmic reticular Ca^{2+} ATPase 2
Cacna1c	calcium voltage-gated channel subunit alpha1 C
Cacna2d1	Calcium Voltage-Gated Channel Auxiliary Subunit Alpha2 delta 1
Camk2d	calcium/calmodulin dependent protein kinase II delta
Pln	Phospholamban
Ppp1ca	Protein phosphatase 1 catalytic subunit alpha
Ppp2ca	Protein phosphatase 2 catalytic subunit alpha
Ppp3ca	Protein phosphatase 3 catalytic subunit alpha
Slc8a1	Solute carrier family 8 member 1 (NCX1)
Prkaca	Protein kinase cAMP-activated catalytic subunit alpha
Casq2	Calsequestrin 2
Adcy5	Adenylyl cyclase 5
Adcy6	Adenylyl cyclase 6
Pde3a	Phosphodiesterase 3A
Pde3b	Phosphodiesterase 3B
Pde4a	Phosphodiesterase 4A
Pde4b	Phosphodiesterase 4B
Pde4c	Phosphodiesterase 4C

Adrb1	Adrenoceptor Beta 1
Adrb2	Adrenoceptor Beta 2
Tnnc1	Troponin C1
Tnnt2	Troponin T2
Tnni3	Troponin I3
Myl1	Myosin light chain 1
Myl2	Myosin light chain 2
Myl3	Myosin light chain 3
Myl4	Myosin light chain 4
Myl5	Myosin light chain 5
Myl6	Myosin light chain 6
Myl12b	Myosin light chain 12b
Mylk3	Myosin light chain kinase 3
Tpm1	Tropomyosin 1
Tpm2	Tropomyosin 2
Tpm3	Tropomyosin 3
Tpm4	Tropomyosin 4
Myh6	Myosin Heavy Chain 6
Myh7	Myosin Heavy Chain 7
Myh9	Myosin Heavy Chain 9
Myh10	Myosin Heavy Chain 10
Myh11	Myosin Heavy Chain 11
Myo1c	Myosin 1c
Myo18a	Myosin 18a

Mybpc3	Myosin Binding Protein C3
