# Supplementary material for: Enrichment of mutant calmodulin protein in a murine model of a human calmodulinopathy
Source: JCI Insight. 2025 Jul 24;10(17):e185524. doi: 10.1172/jci.insight.185524 (PMC12487685; doi:10.1172/jci.insight.185524)

Cardiac Enrichment of Mutant Calmodulin Protein in a Murine Model of a Human Calmodulinopathy

- Full gel images for cropped panels in Western blot and Restriction Fragment Length Polymorphism

- Figure 2A, Figure 4A&C, and Supplemental Figure S1

Figure 2A

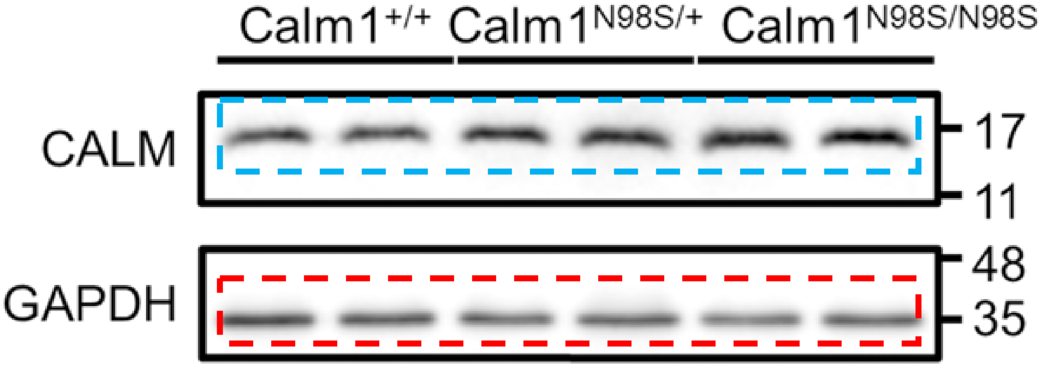

CaM/marker

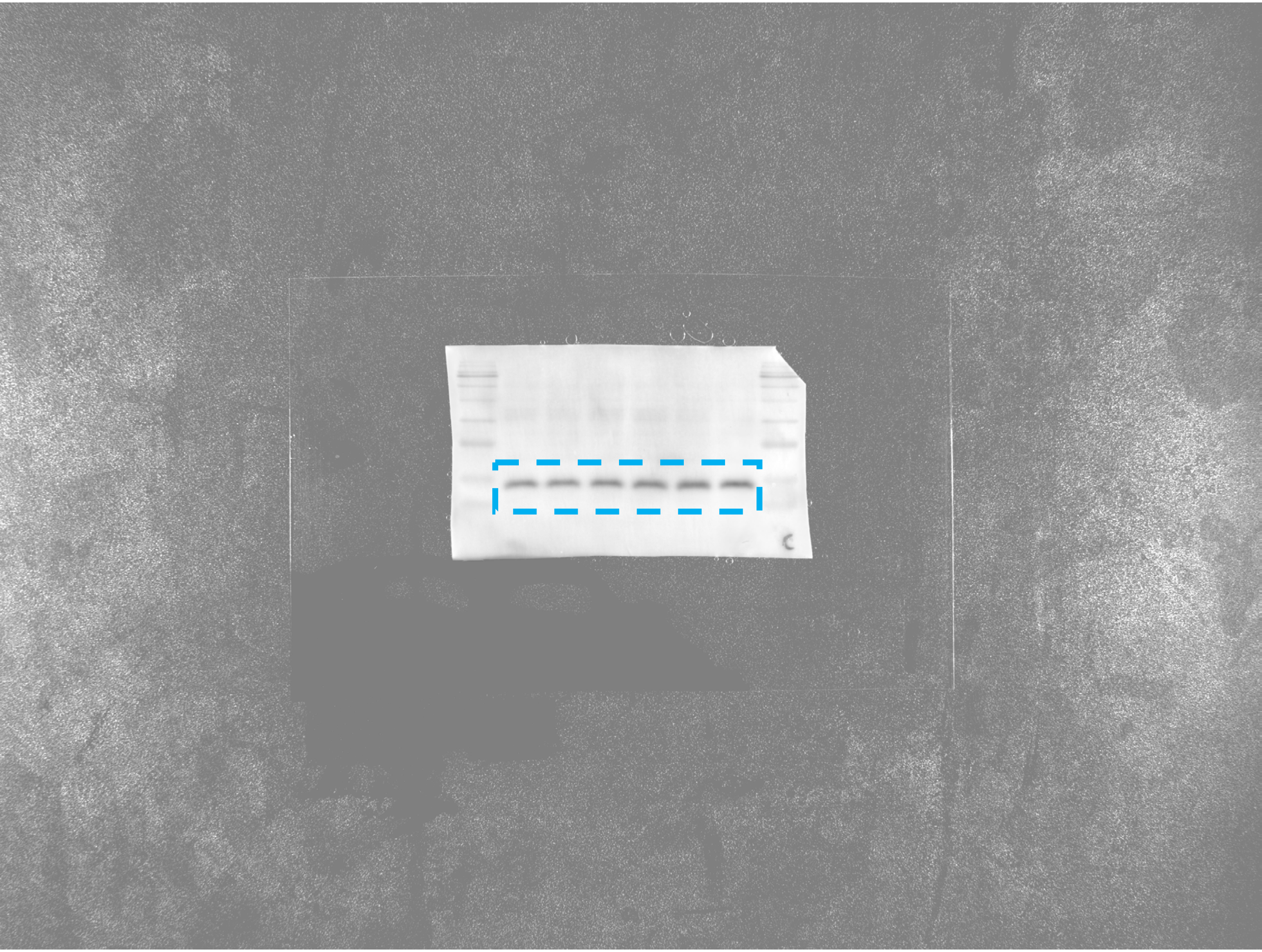

GAPDH/marker

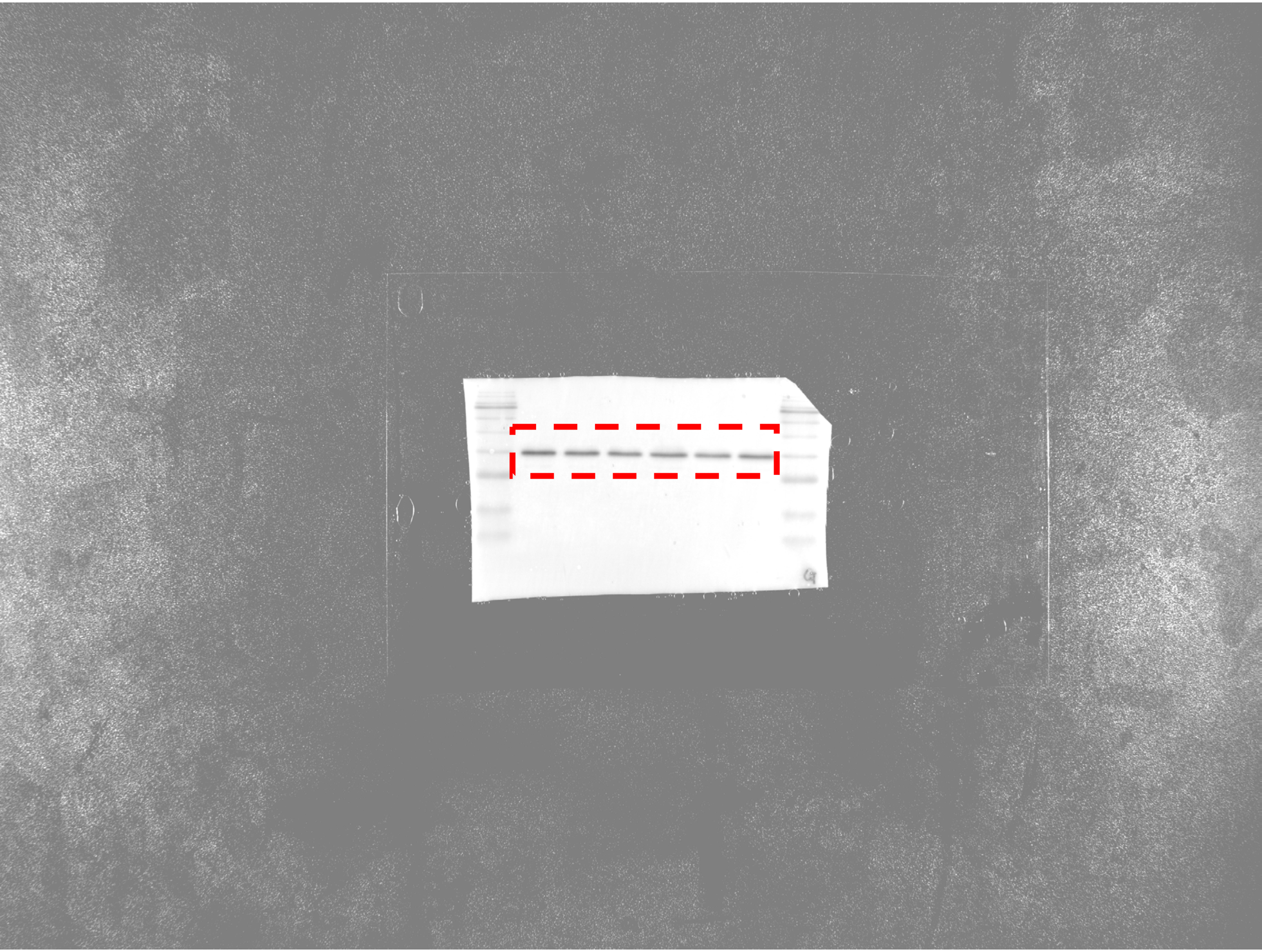

Figure 4A (Non-transfection and HA Calm-HA)

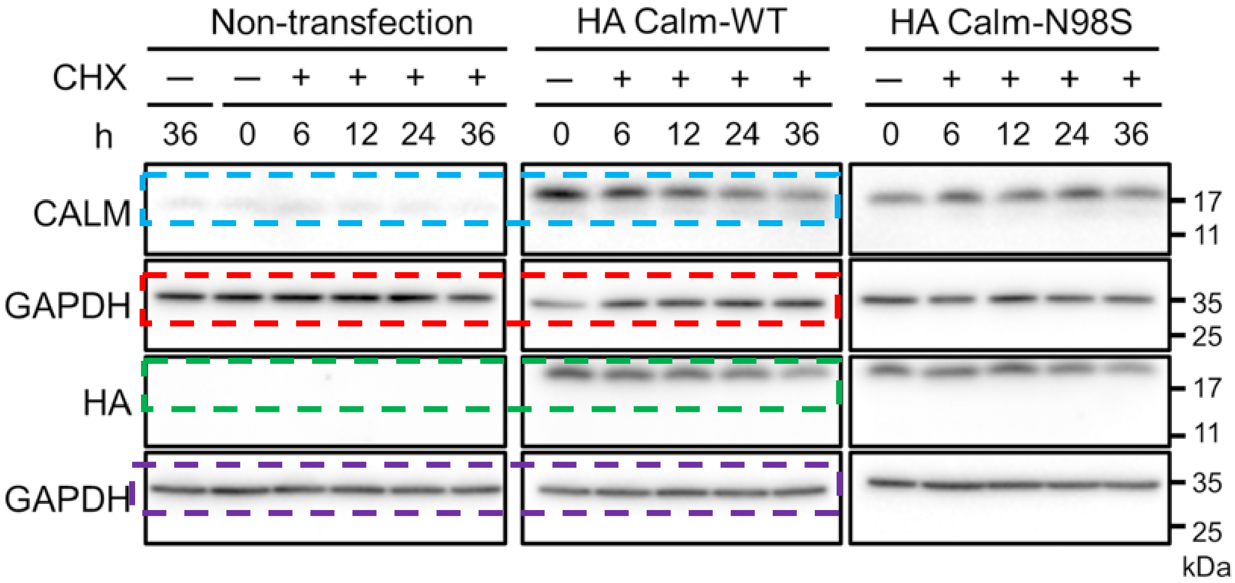

CaM/marker

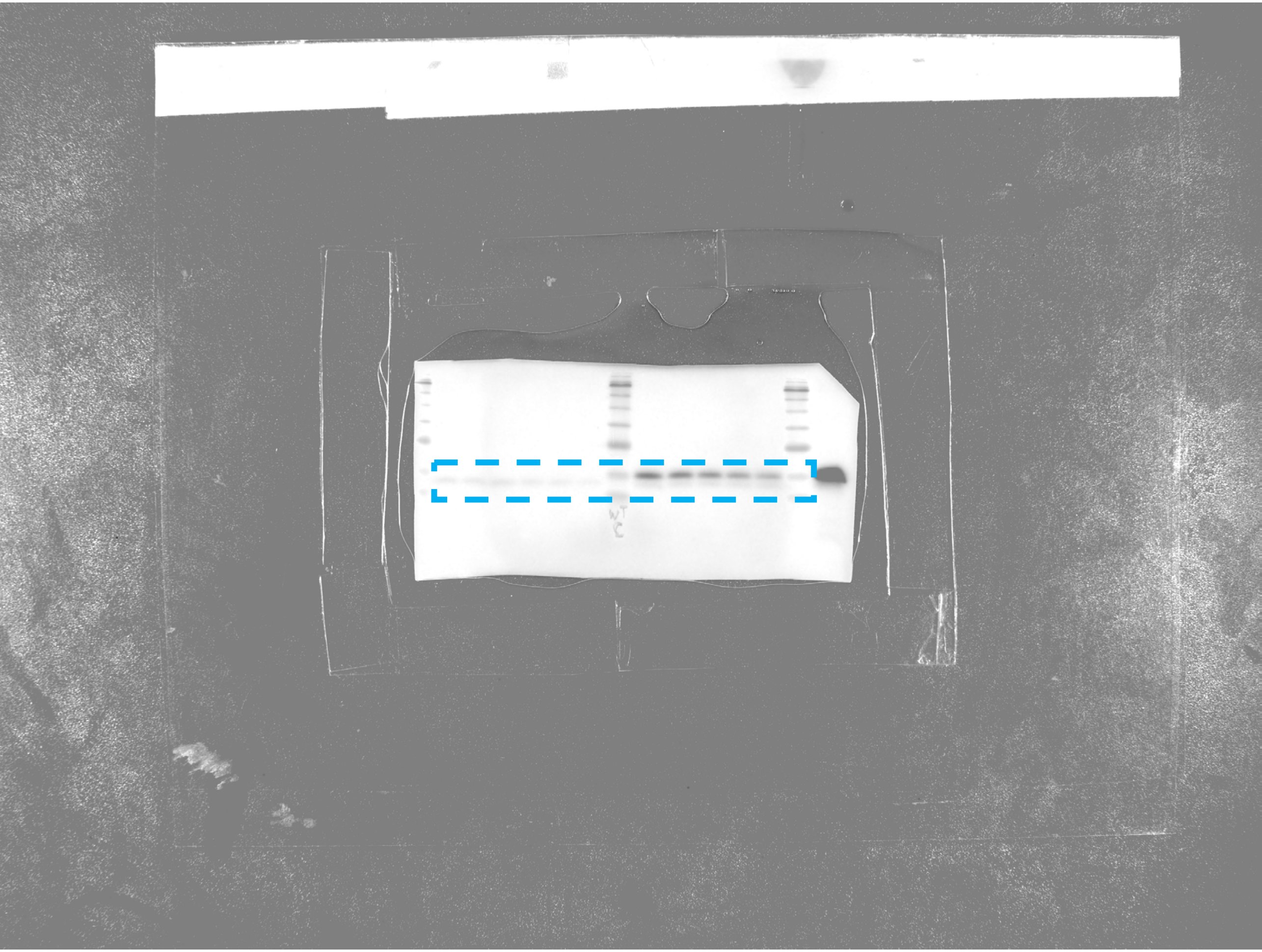

CaM-GAPDH/marker

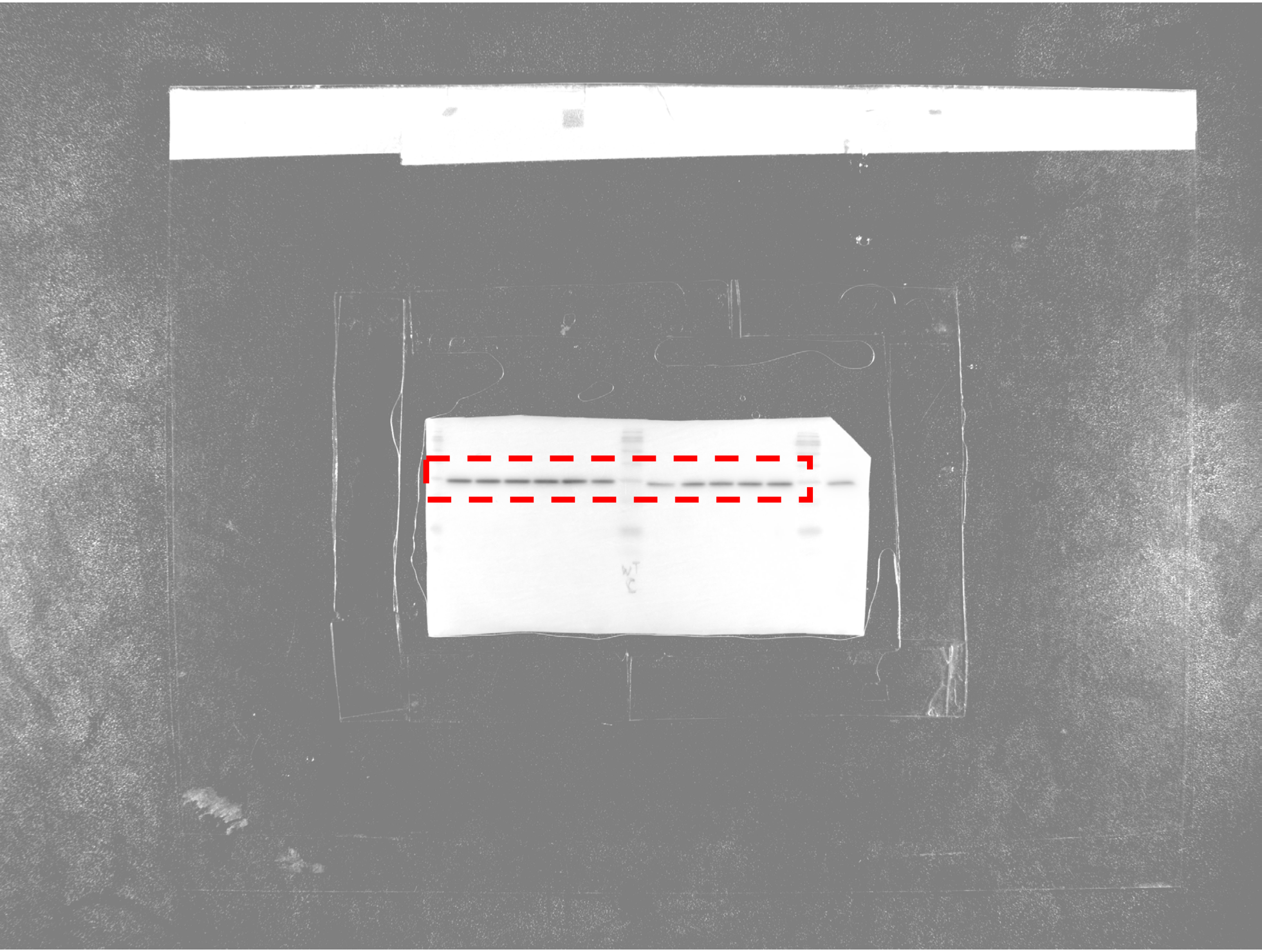

HA/marker

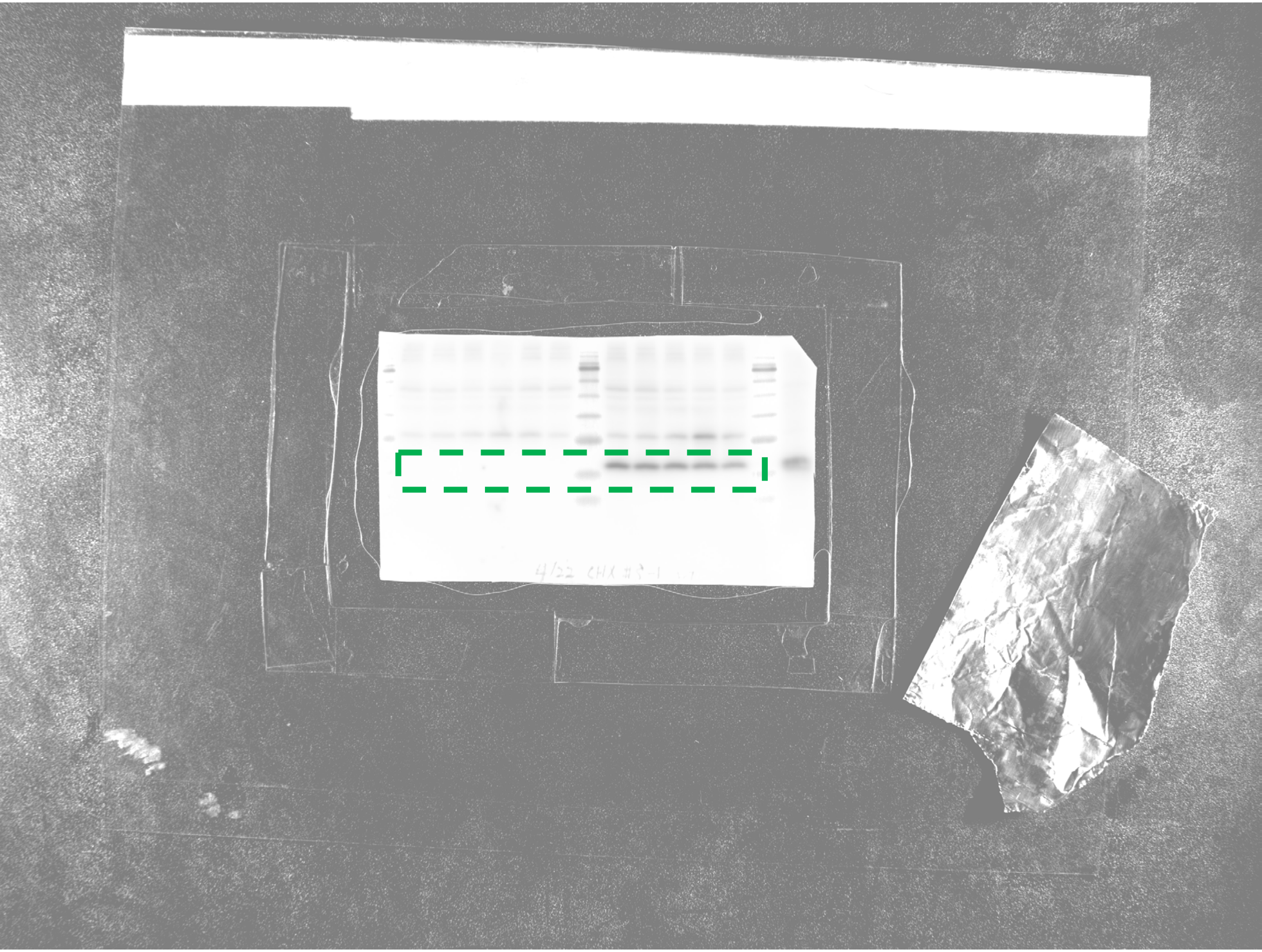

HA-GAPDH/marker

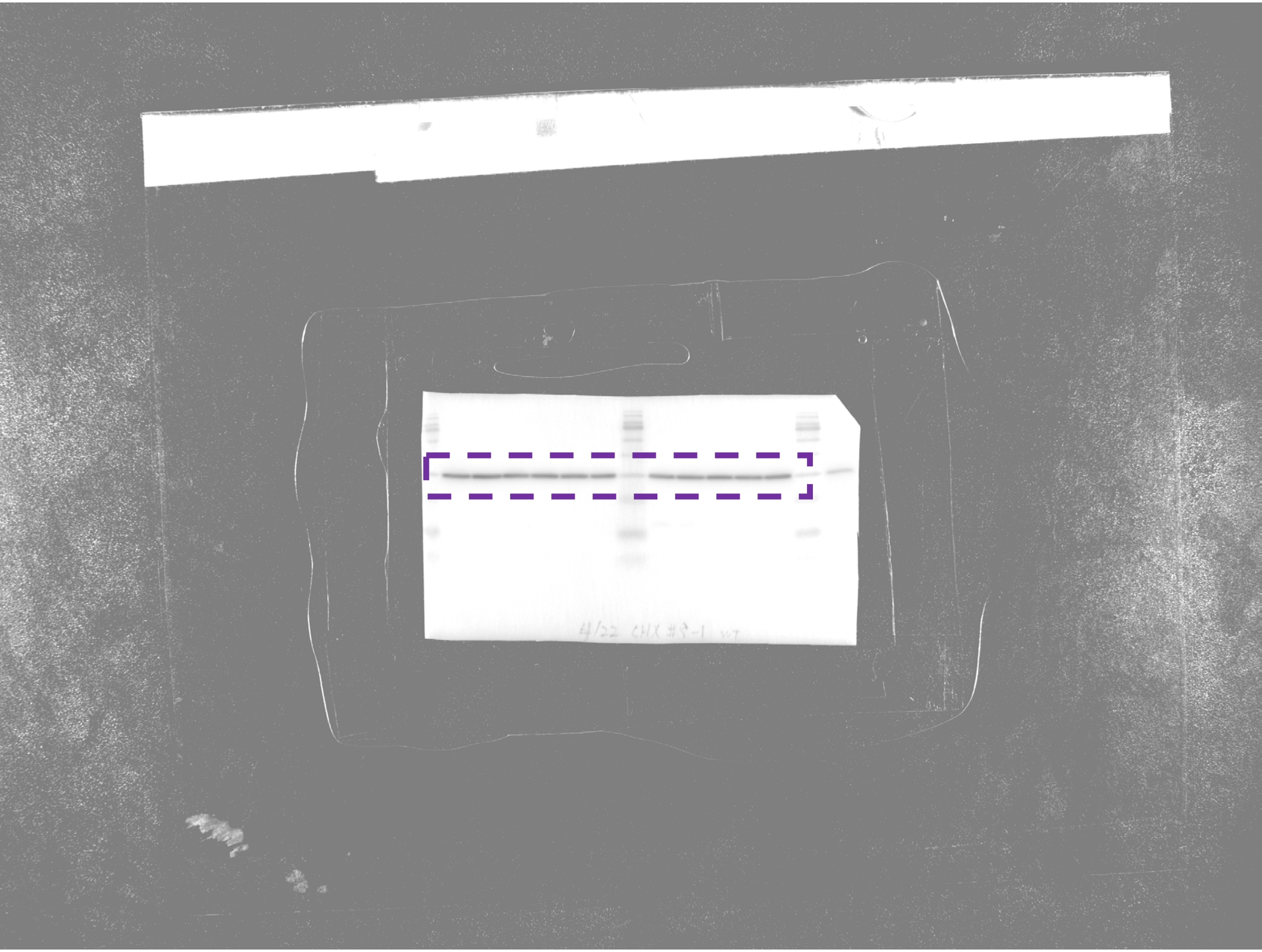

Figure 4A (HA Calm-N98S)

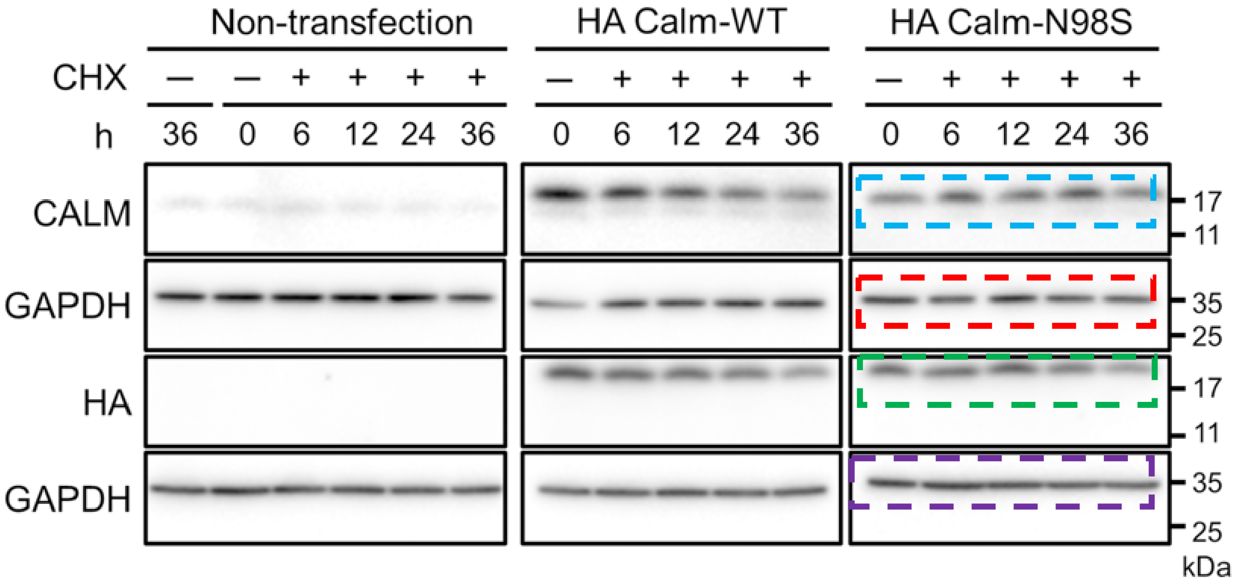

CaM/marker

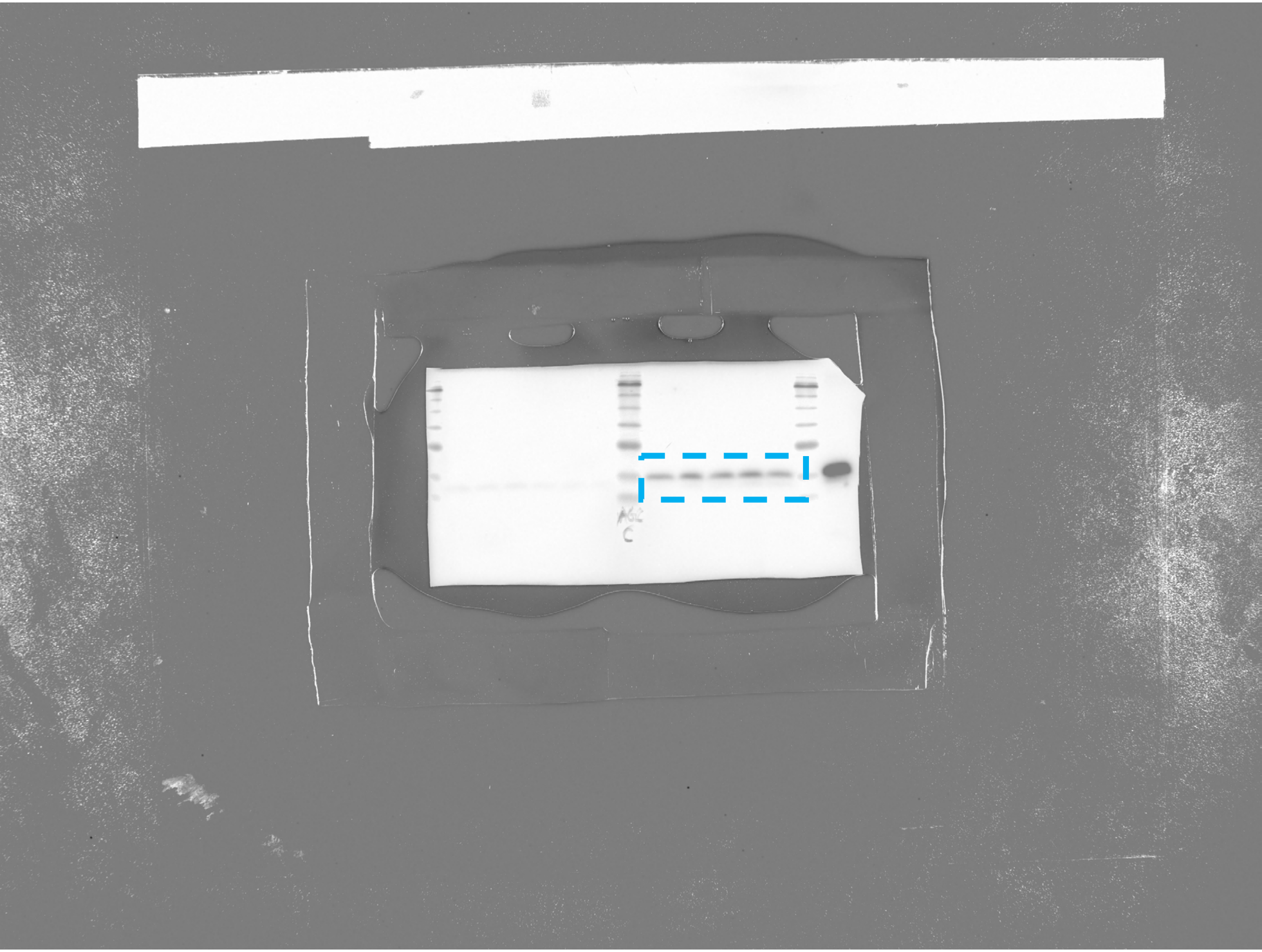

CaM-GAPDH/marker

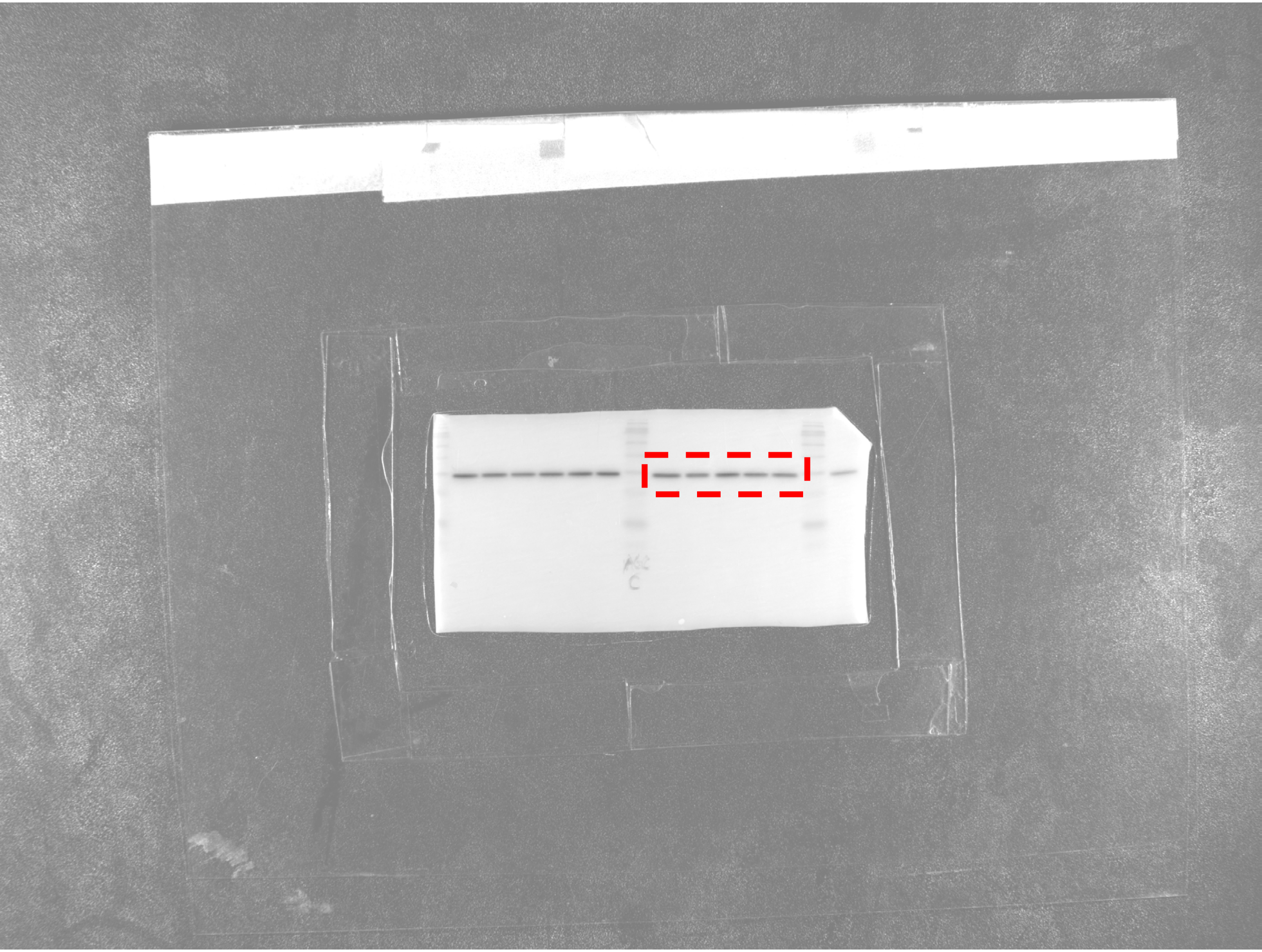

HA/marker

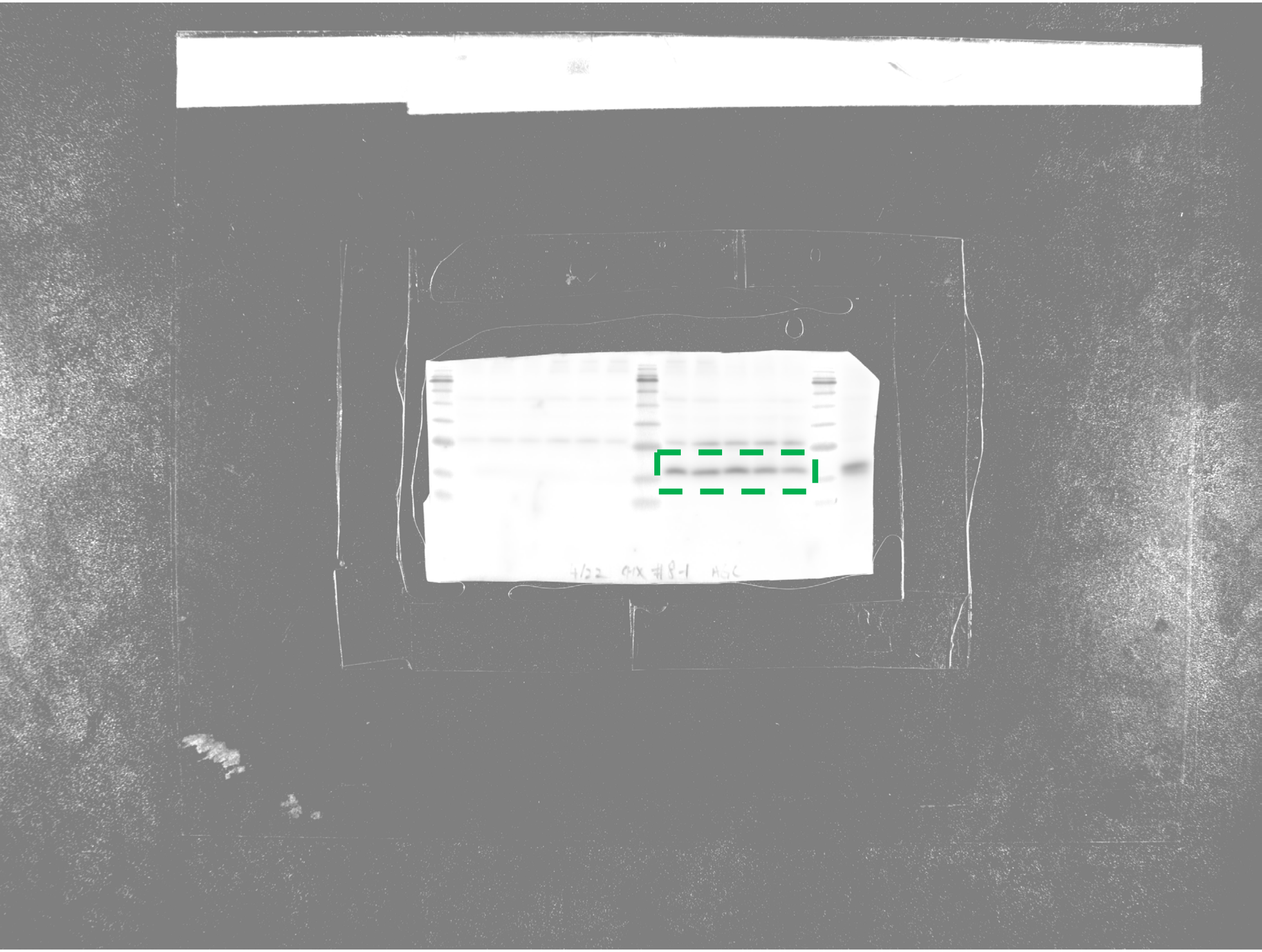

HA-GAPDH/marker

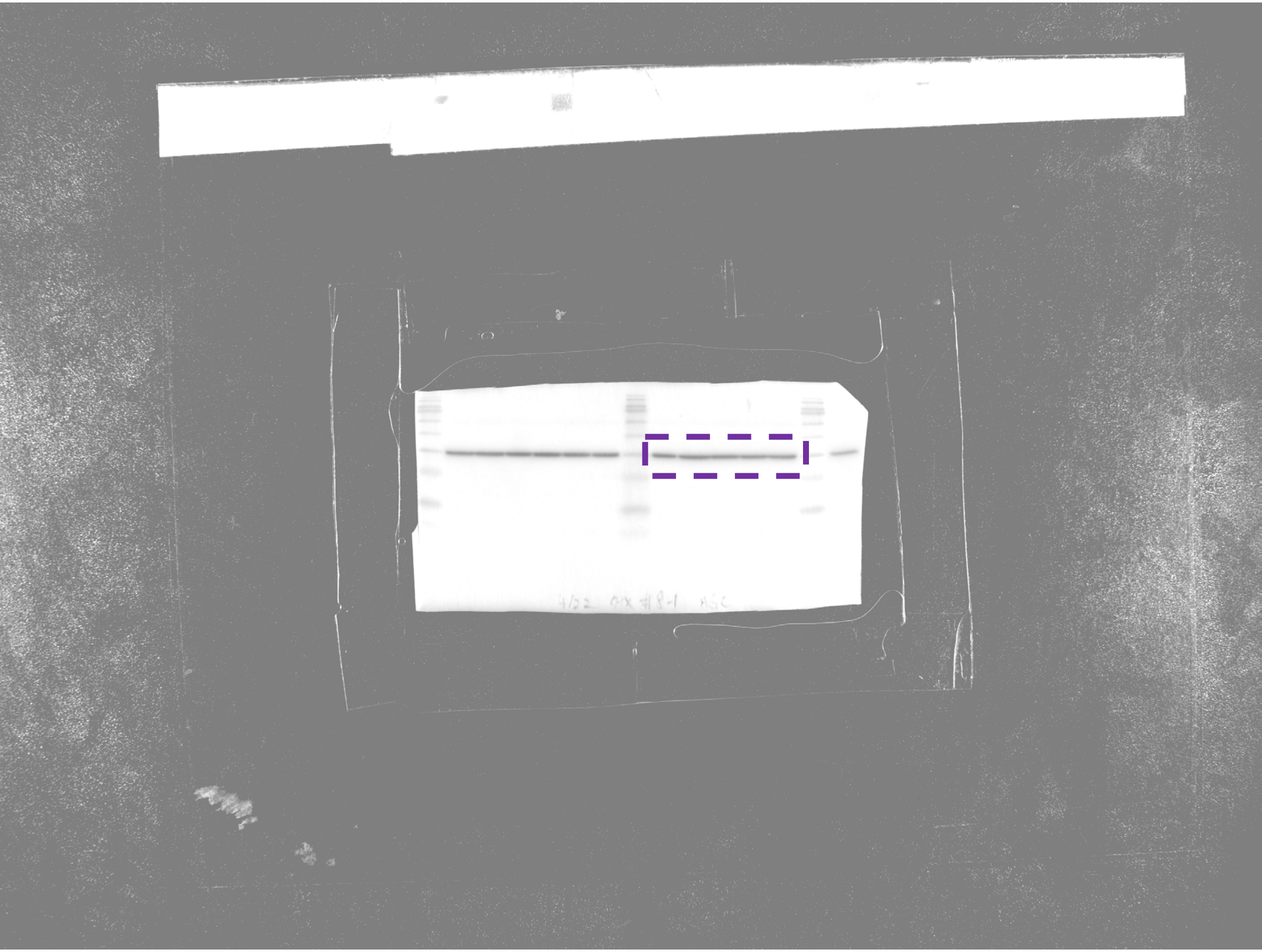

Figure 4C

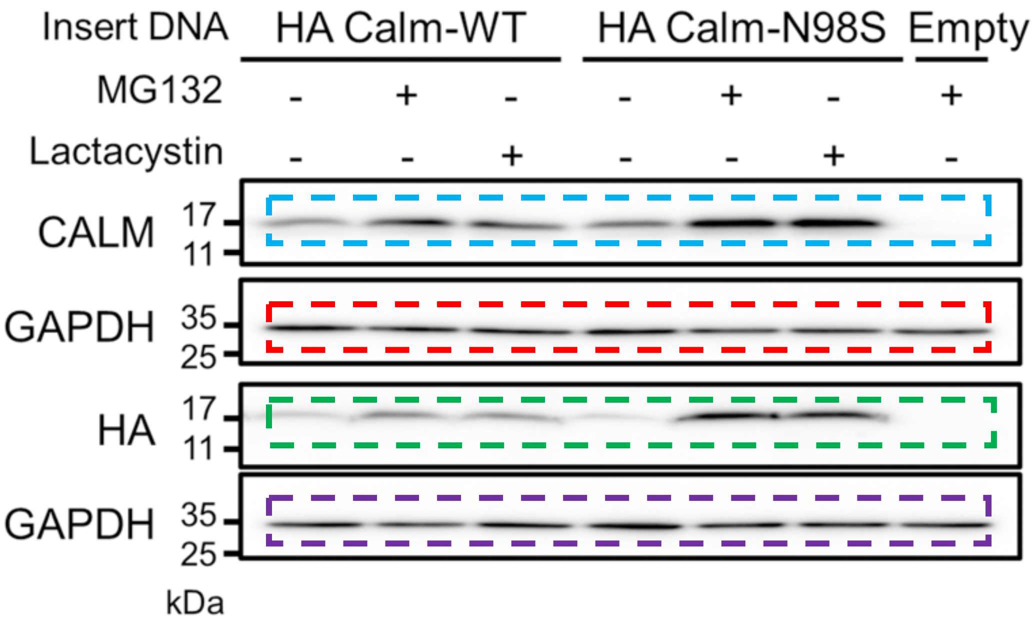

Insert DNA
HA Calm-WT
HA Calm-N98S
Empty
MG132
- + - - + - +
Lactacystin
- - + - - + -
CALM
17
11
GAPDH
35
25
HA
17
11
GAPDH
35
25
kDa

CaM/marker

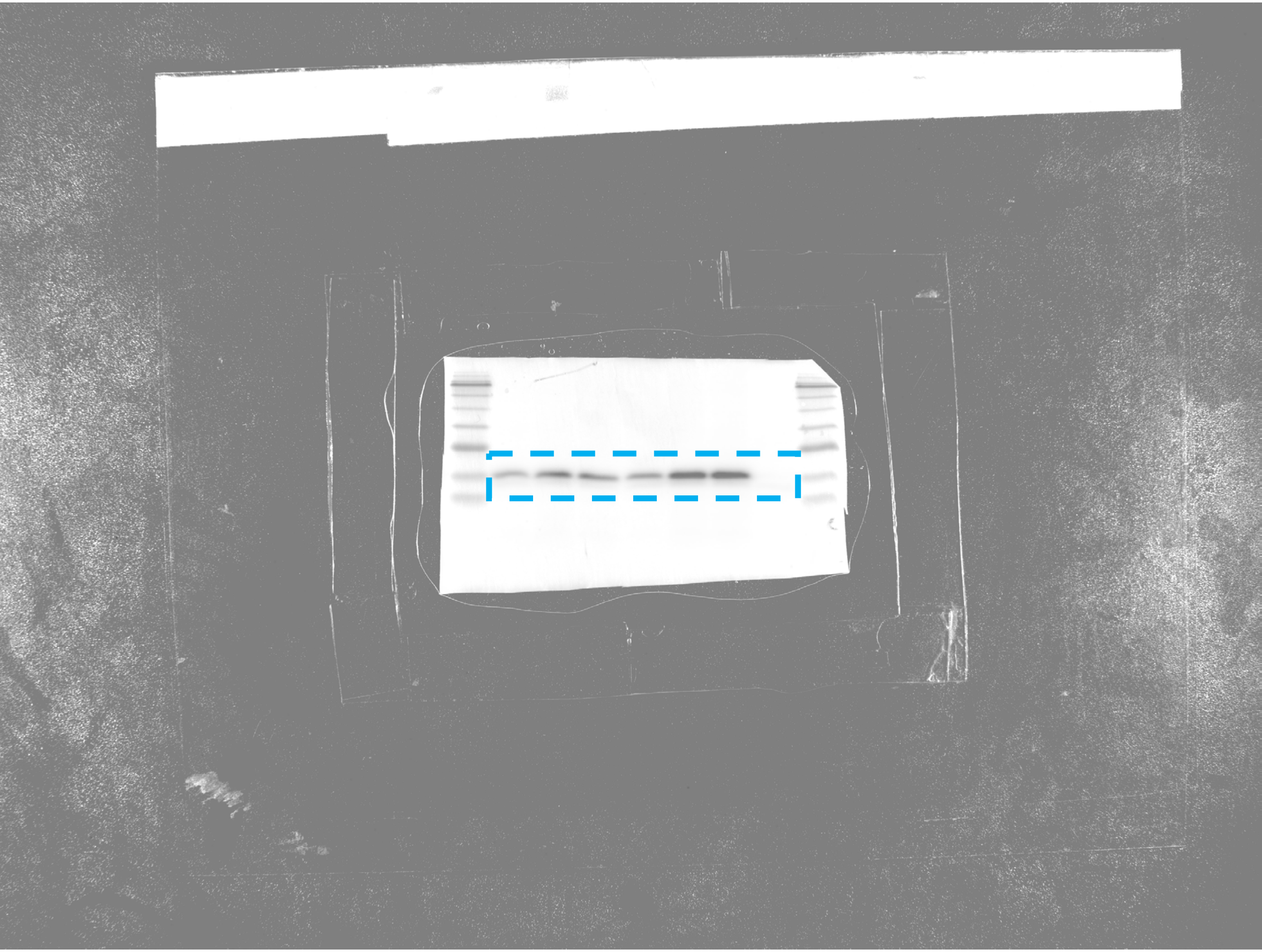

CaM-GAPDH/marker

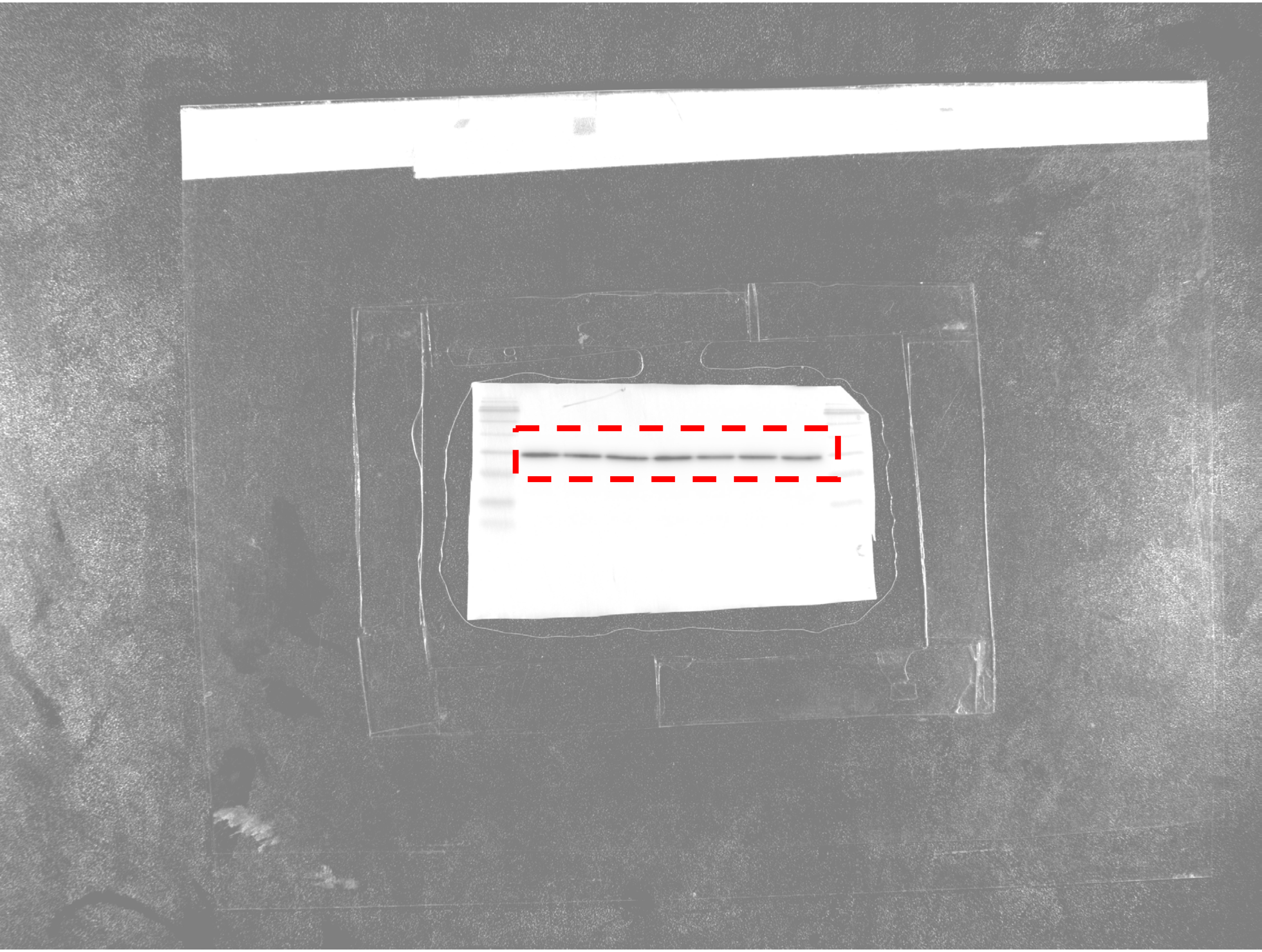

HA/marker

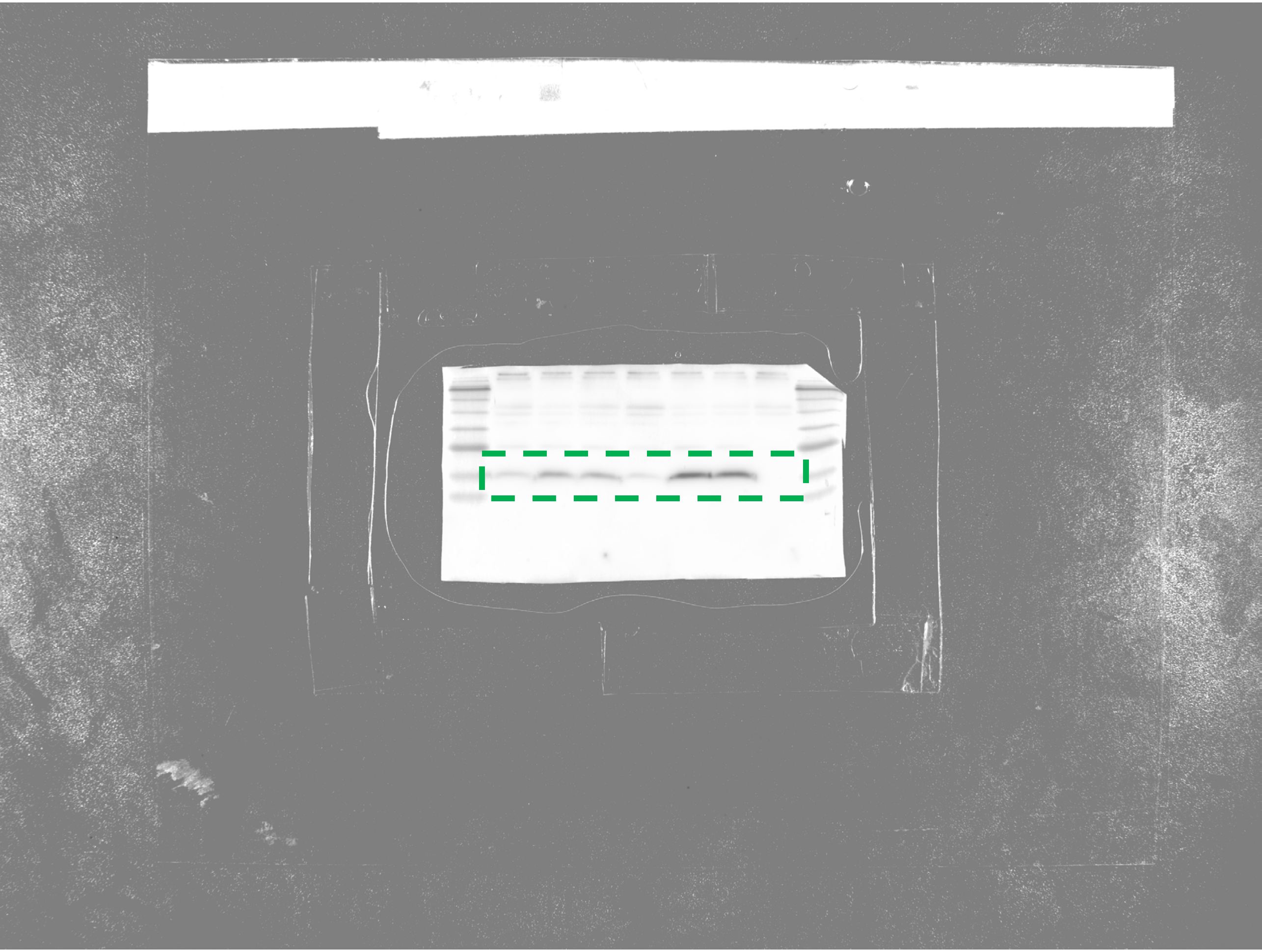

HA-GAPDH/marker

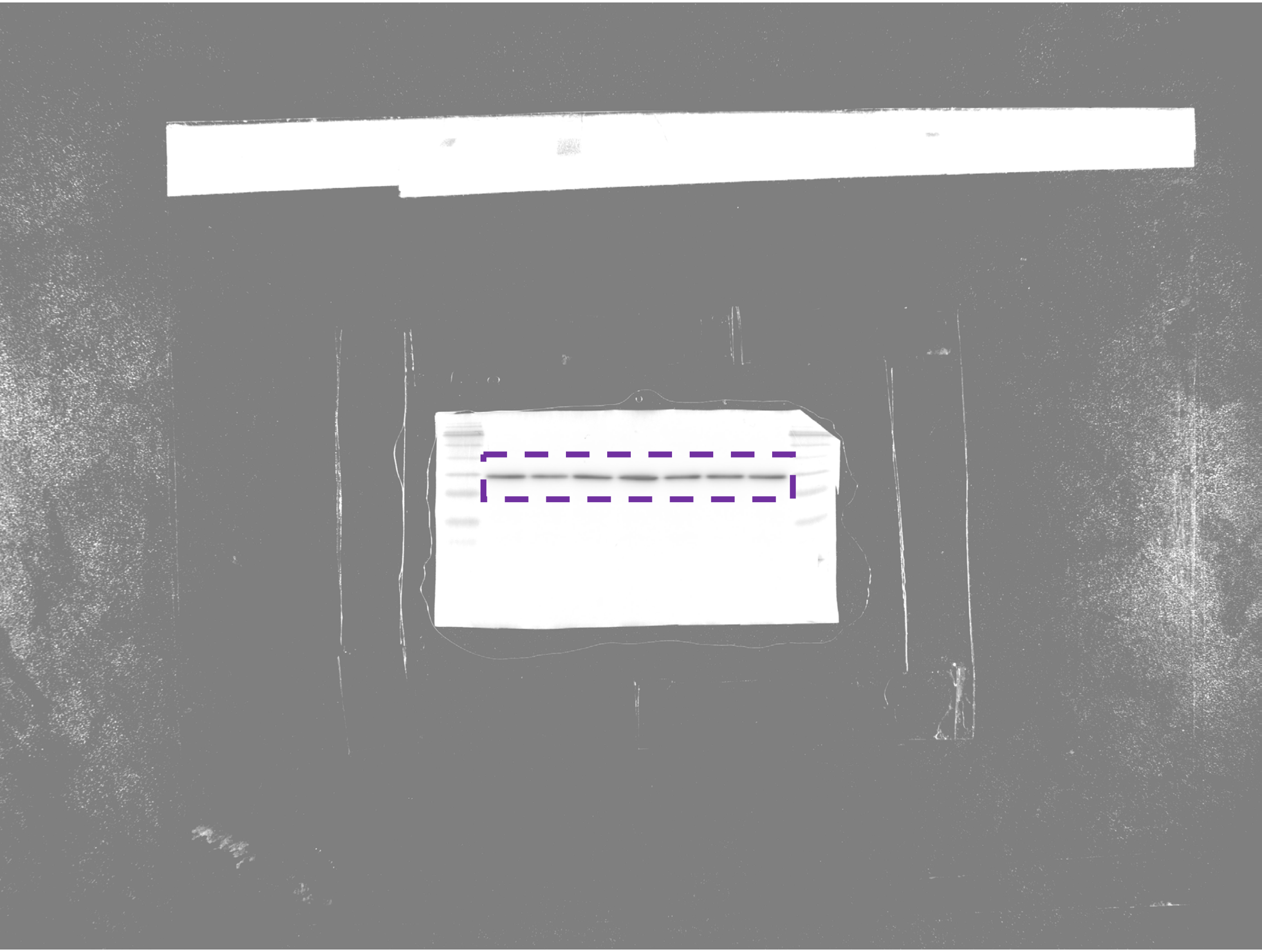

Supplemental Figure S1

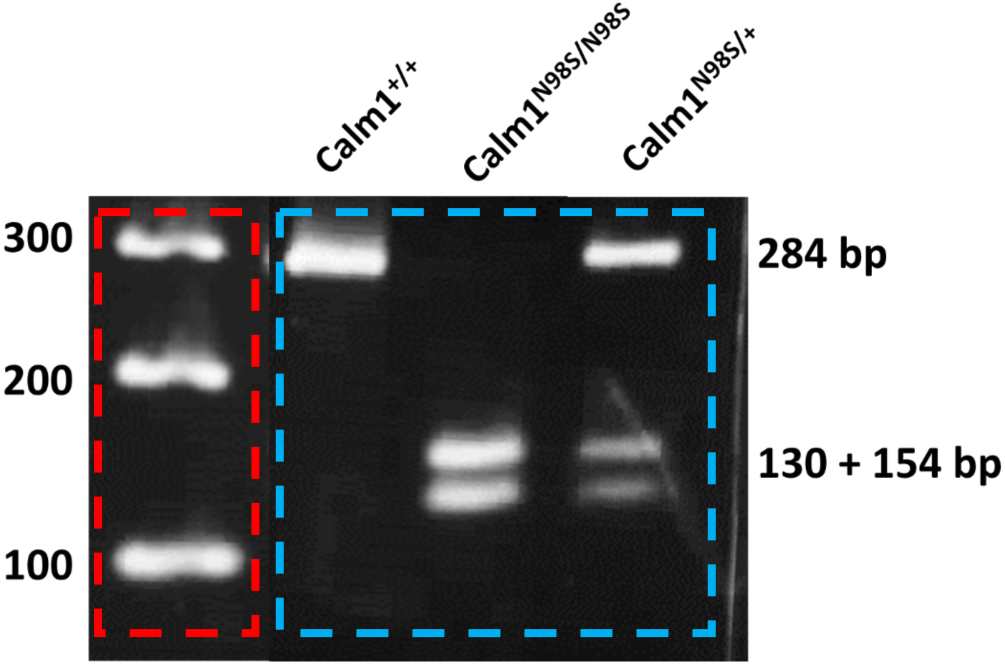

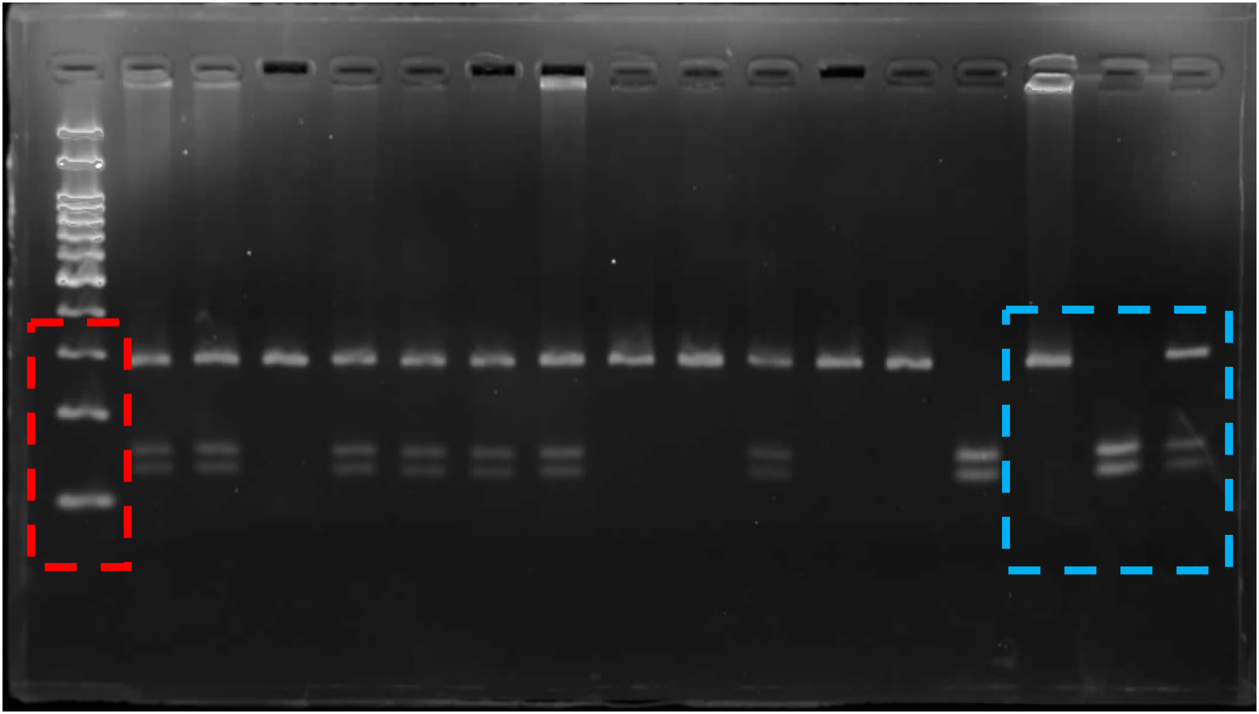

Supplement: Unedited blot and gel images [file jciinsight-10-185524-s009.pdf]
